# Bioactive lipid mediators in plasma are predictors of preeclampsia irrespective of aspirin therapy

**DOI:** 10.1016/j.jlr.2023.100377

**Published:** 2023-04-27

**Authors:** Daniel J. Stephenson, H. Patrick MacKnight, L. Alexis Hoeferlin, Sonya L. Washington, Chelsea Sawyers, Kellie J. Archer, Jerome F. Strauss, Scott W. Walsh, Charles E. Chalfant

**Affiliations:** 1 Division of Hematology & Oncology, Department of Medicine, University of Virginia, Charlottesville, VA, USA; 2 Department of Biochemistry and Molecular Biology, Virginia Commonwealth University (VCU), Richmond, VA, USA; 3 Department of Obstetrics and Gynecology, Virginia Commonwealth University, Richmond, VA, USA; 4 Virginia Institute for Psychiatric & Behavioral Genetics, Virginia Commonwealth University School of Medicine, Richmond, VA, USA; 5 Division of Biostatistics, The Ohio State University College of Public Health, Columbus, OH, USA; 6 Department of Cell Biology, University of Virginia, Charlottesville, VA, USA; 7 Program in Cancer Biology, University of Virginia Cancer Center, Charlottesville, VA, USA; 8 Research Service, Richmond Veterans Administration Medical Center, Richmond, VA, USA

**Keywords:** eicosanoids, sphingolipids, pregnancy, aspirin, ultra-high performance liquid chromatography electrospray ionization-MS/MS

## Abstract

There are few early biomarkers to identify pregnancies at risk of preeclampsia (PE) and abnormal placental function. In this cross-sectional study, we utilized targeted ultra-performance liquid chromatography-ESI MS/MS and a linear regression model to identify specific bioactive lipids that serve as early predictors of PE. Plasma samples were collected from 57 pregnant women prior to 24-weeks of gestation with outcomes of either PE (n = 26) or uncomplicated term pregnancies (n = 31), and the profiles of eicosanoids and sphingolipids were evaluated. Significant differences were revealed in the eicosanoid, (±)11,12 DHET, as well as multiple classes of sphingolipids; ceramides, ceramide-1-phosphate, sphingomyelin, and monohexosylceramides; all of which were associated with the subsequent development of PE regardless of aspirin therapy. Profiles of these bioactive lipids were found to vary based on self-designated race. Additional analyses demonstrated that PE patients can be stratified based on the lipid profile as to PE with a preterm birth linked to significant differences in the levels of 12-HETE, 15-HETE, and resolvin D1. Furthermore, subjects referred to a high-risk OB/GYN clinic had higher levels of 20-HETE, arachidonic acid, and Resolvin D1 versus subjects recruited from a routine, general OB/GYN clinic. Overall, this study shows that quantitative changes in plasma bioactive lipids detected by ultra-performance liquid chromatography-ESI-MS/MS can serve as an early predictor of PE and stratify pregnant people for PE type and risk.

Preeclampsia (PE) is a hypertensive disorder and a serious complication in pregnant women that affects 5–7% of all pregnancies in the United States. PE is diagnosed based on a constellation of symptoms and clinical features including arterial hypertension, proteinuria, edema, hepatic dysfunction, and hypercoagulation, which can be reflected by thrombocytopenia and abnormal serum hepatic enzyme levels. These clinical symptoms of PE begin after 20 weeks of pregnancy, and PE can be further subclassified into early-onset PE (manifesting before 34 weeks of pregnancy) and late-onset PE (manifesting at or later than 34 weeks of pregnancy). The disease is thought to be caused by placental dysfunction and only occurs in the presence of the placenta or placental tissue (1, 2, 3, 4, 5, 6, 7). PE is associated with life-threatening complications such as stroke, steatohepatitis, and renal failure (8), as well as an increased risk of maternal heart disease later in life (3, 9, 10, 11, 12, 13, 14, 15, 16, 17, 18). Hypertensive complications during pregnancy, particularly PE, rank second in the world among the causes of maternal mortality. Annually, 70,000 pregnant women die from PE and its complications worldwide (19).

Despite decades of research, the etiology of PE is not fully understood. A known link to causation of PE is altered levels of two bioactive lipids, thromboxane (TXB_2_) and prostacyclin (3, 20, 21), and the imbalance of the biological actions of these two lipid mediators is linked to the major clinical symptoms of PE such as hypertension, platelet aggregation, and reduced uteroplacental blood flow. Thromboxanes and prostacyclins are part of the bioactive lipid class of eicosanoids, which are oxidized derivatives of arachidonic acid (AA), EPA, and DHA (22). Besides thromboxanes and prostacyclins, subclasses of eicosanoids include prostaglandins, leukotrienes, and HETEs as well as omega 3 polyunsaturated fatty acid-derived lipid mediators (e.g., resolvins, maresins) (22). The synthesis of eicosanoids begins with the initial rate-limiting step, the release of AA, EPA, and DHA via the activity of a phospholipase A_2_ (15). The cyclooxygenase (COX) enzymes, COX-1 and COX-2, utilize these fatty acids to produce a variety of eicosanoids including prostacyclins and thromboxanes (22).

The COX enzymes are the targets of aspirin, and low-dose aspirin use in humans selectively inhibits platelet thromboxane synthesis without affecting endothelial prostacyclin synthesis (8, 19, 23, 24, 25). A key pathophysiologic role for thromboxanes in PE was disclosed in low-dose aspirin studies (10, 26, 27, 28, 29, 30, 31) starting with the first clinical trial of low-dose aspirin to prevent PE by Wallenburg *et al.*, reported in 1986 (29). The study rationale was based on the finding of increased thromboxane and decreased prostacyclin production by placentas of women with PE (3). A plethora of clinical trials followed with meta-analyses showing that in almost all trials of low-dose aspirin, the incidence of PE decreased (32, 33, 34). Low-dose aspirin (50–150 mg/day) is now the standard of care to prevent PE in at-risk women (30). It is effective in preventing PE (∼50%) when administered to high-risk individuals prior to 16 weeks of gestation (35).

Since aspirin therapy is effective in only 50% of patients, the scientific community has undertaken additional studies to examine circulating levels of eicosanoids and other lipids in pregnant women that potentially predict and/or possibly cause PE. In this regard, studies have shown that higher levels of 11,12-epoxyeicosatrienoic acid (EET), 5-HETE, 8-HETE, 12-HETE, and 15-HETE are present in the sera at 20 weeks of gestation in pregnancies complicated by PE (36). A significant elevation in triglycerides is present as early as 10 weeks of gestation in women who develop PE (37, 38, 39), and proinflammatory omega-6 polyunsaturated fatty acids (e.g., AA) are elevated, whereas anti-inflammatory omega-3 polyunsaturated fatty acids (e.g., EPA, DHA) are decreased compared to normal pregnancy (40, 41). Austdal *et al.* observed an increase in the content of the phosphatidylcholine species (PC_14:0/00_), VLDLs, and LDLs in serum samples from pregnant women with PE (42). Sphingolipids, a class of complex bioactive lipids, have also been shown to be dysregulated in PE. For example, studies found that in the first trimester, the maternal plasma ceramide (CER) species (de_18:1/20:0_ and de_18:1/14:0_ CERs) and SM species (SM de_16:0_ and SM de_18:1_) may be early biomarkers of PE (43). A study by Park *et al.* also showed SM dysregulation (very long-chain SM species) in the plasma of pregnant women linked to later PE development (44). In the same women with PE whose serum sphingosine-1-phosphate (S1P) was lower than in healthy controls, higher de_18:1/16:0_, de_18:1/18:0_, de_18:1/20:0_, and de_18:1/24:0_ CER concentrations were found in both serum and placental tissue. Additionally, a number of studies have demonstrated increased levels of S1P, sphingosine (So), total CER, and specific CER species linked to complicated pregnancies and PE examining plasma samples after 24 weeks (45, 46, 47). In contrast, Johnstone *et al.* showed no correlation between plasma S1P levels and PE development using stored blood samples from 95 women (14–24 weeks of gestation) at risk of developing PE (48).

Even with accumulating evidence that additional bioactive lipids are predictive of later PE development, there is no accepted method for predicting PE risk based on lipid analysis (2, 3, 13, 21, 37, 49, 50, 51). Unfortunately, the findings from studies noted above on lipid-based biomarkers conflict with other reports in the literature and also predate the standard use of aspirin in at-risk pregnancies. Furthermore, fundamental questions remain as follows: are there early lipid-based markers that predict subsequent onset of PE regardless of aspirin-therapy? Can lipid-based biomarkers distinguish PE development linked to preterm birth, as well as mild versus PE with severe features? Are there race-specific lipid alterations linked to PE development? The purpose of this cross-sectional study was to identify a lipid “fingerprint” that clarifies these understudied questions for more precision-based, tailored analyses of a patient’s risk for developing PE. Our study shows that specific eicosanoids and sphingolipids can be accurately measured in the plasma of pregnant women and serve as early biomarkers of the later development of PE with severe features, regardless of aspirin therapy, as well as stratify patients into PE with or without a preterm birth and racial-specific risk for PE development.

## Materials and methods

### Study subjects and ethical considerations

Whole blood (6 ml) was collected in K2 EDTA 10.8 mg tubes (BD Vacutainer) from uncomplicated term pregnancies (n = 31) and preeclamptic pregnancies (n = 26) from women (Table 1). The mean gestational age at plasma collection was 14.9 weeks for uncomplicated term pregnancies and 16.5 weeks for preeclamptic pregnancies at MCV Hospital, Virginia Commonwealth University Medical Center, Richmond, VA prior to the onset and diagnosis of PE. Subjects were recruited from our high-risk (14 uncomplicated term pregnancies and 22 preeclamptic pregnancies) and general (17 uncomplicated term pregnancies and 4 preeclamptic pregnancies) obstetrics clinics. Subjects from our high-risk clinic were prescribed low-dose aspirin (81 mg/d) according to the guidelines of the American College of Obstetrics and Gynecology, but compliance was not confirmed. Whole blood was processed to plasma within 2 h of acquisition, stored at −80°C in 0.75 ml aliquots, and analyzed for the levels of bioactive lipids within 2 weeks by ultra-performance liquid chromatography (UPLC) ESI-MS/MS. PE was later diagnosed by new onset hypertension (systolic blood pressure of ≥140 mm Hg and/or diastolic blood pressure ≥90 mm Hg) measured on two occasions at least 4 h apart and proteinuria (protein/creatinine ratio ≥0.3). All subjects gave informed consent, and the procedures followed were in accordance with institutional guidelines. This study was approved by the Office of Research Subjects Protection, Virginia Commonwealth University, Richmond, VA (HM20005160) and adhered to the Declaration of Helsinki principles.

**Table 1:** Subject demographics and clinical characteristics

Variable	NP n = 31 (54%)	PE n = 26 (46%)
Maternal age (years)	28.2 ± 5.2	30.3 ± 7.6
Prepregnancy BMI (kg/m^2^)	25.1 ± 5.2	29.4 ± 7.4
BMI at sample collection (kg/m^2^)	30.2 ± 5.1	34.3 ± 7.1
Systolic blood pressure (mmHg)	116.8 ± 14.8	150.9 ± 19.1∗∗∗∗
Diastolic blood pressure (mmHg)	72.9 ± 8.9	91.3 ± 14.1∗∗∗∗
Primigravida	5	3
Multigravida	24	23
Race		
White	7 (23%)	5 (19%)
Black	20 (65%)	13 (50%)
Hispanic	4 (12%)	8 (31%)
Delivery Method		
Vaginal	23 (79%)	14 (54%)
C-Section	6 (21%)	12 (46%)
Gestational age at sample (weeks)	14.89 ± 3.8	16.5 ± 5.2
Gestational age at birth (weeks)	39.0 ± 1.2	35.4 ± 4.8∗∗∗∗
Infant birth weight (grams)	3053.5 ± 428.1	2425.25 ± 1184.0∗∗
Smokers	12	7
Illicit drug use	4	1
BMI >30	4	12

A description of included patients are as follows, n = 31 normal pregnancies and n = 26 PE pregnancies prior to 24 weeks gestation. Unpaired Student's *t* test with Welch’s correction was performed for all groups. The blood pressures depicted were taken at the time of sample collection. All samples were collected prior to 24 weeks of gestation, processed within 2 h of collection, stored as aliquoted plasma samples at −80°C, and analyzed by UPLC ESI-MS/MS within 2 weeks of acquisition. Where applicable, the data are presented as means ± SD. Significance between uncomplicated term pregnancies and PE pregnancies is represented as ∗∗*P* < 0.01 and ∗∗∗∗*P* < 0.0001.

### LC/MS analyses

#### Analysis of eicosanoids by UPLC ESI-MS/MS

Eicosanoids were extracted and analyzed by UPLC ESI-MS/MS as previously described by us and others (52, 53, 54, 55, 56, 57, 58) (Table 2). Briefly, plasma (200 μl) was combined with 800 μl of LCMS water followed by the addition of an internal standard mixture comprised of 10% methanol (100 μl), glacial acetic acid (5 μl), and internal standard (20 μl) containing the following deuterated eicosanoids (1.5 pmol/μl, 30 pmol total) (All standards purchased from Cayman Chemicals): (*d*_4_) 6keto-prostaglandin F_1_α, (*d*_4_) prostaglandin F_2_α, (*d*_4_) prostaglandin E_2_, (*d*_4_) prostaglandin D_2_, (*d*_8_) 5-HETE, (*d*_8_) 12-HETE, (*d*_8_) 15-HETE, (*d*_6_) 20-HETE, (*d*_*11*_) 8,9 epoxyeicosatrienoic acid, (*d*_*8*_) 14,15 epoxyeicosatrienoic acid, (*d*_8_) AA, (*d*_5_) EPA, (*d*_5_) DHA, (*d*_4_) prostaglandin A2, (*d*_4_) leukotriene B4, (*d*_4_) leukotriene C4, (*d*_4_) leukotriene D4, (*d*_4_) leukotriene E4, (*d*_5_) 5(S),6(R)-lipoxin A4, (*d*_11_) 5-iPF2α-VI, (*d*_4_) 8-iso prostaglandin F2α, (*d*_11_) (±)14,15- dihydroxyeicosatrienoic acid (DHET), (*d*_11_) (±)8,9-DHET, (*d*_11_) (±)11,12-DHET, (*d*_4_) prostaglandin E1, (*d*_4_) thromboxane B2, (*d*_6_) dihomo gamma linoleic acid, (*d*_5_) resolvin D2, (*d*_5_) resolvin D1 (RvD1), (*d*_5_) maresin2, and (*d*_5_) resolvin D3. Samples and vial rinses (5% methanol; 2 ml) were applied to Strata-X solid phase extraction (SPE) columns (Phenomenex), previously washed with methanol (2 ml) and then dH_2_O (2 ml). Eicosanoids eluted with isopropanol (2 ml) were dried *in vacuo* and reconstituted in EtOH:dH_2_O (50:50;100 μl) prior to UPLC ESI-MS/MS analysis.

**Table 2:** Lipid classes and lipids analyzed via UPLC ESI-MS/MS

Lipid Category	Lipid Class	Tested Lipids			
**Sphingolipids**	**Ceramides**	Cer(de18:1/14:0)	Cer(de18:1/18:1)	Cer(de18:1/22:0)	Cer(de18:1/26:1)
		Cer(de18:1/16:0)	Cer(de18:1/18:0)	Cer(de18:1/24:1)	Cer(de18:1/26:0)
			Cer(de18:1/20:0)	Cer(de18:1/24:0)	
	**Ceramide - 1 - Phosphates**	C1P(de18:1/14:0)	C1P(de18:1/18:1)	C1P(de18:1/22:0)	C1P(de18:1/26:1)
		C1P(de18:1/16:0)	C1P(de18:1/18:0)	C1P(de18:1/24:1)	C1P(de18:1/26:0)
			C1P(de18:1/20:0)	C1P(de18:1/24:0)	
	**Long-chain bases**	de18:1 So	de18:1 So1P	de18:0 Sa	de18:0 Sa1P
	**Glycosphingolipids**	MonHex(de18:1/14:0)	MonHex(de18:1/18:1)	MonHex(de18:1/22:0)	MonHex(de18:1/26:1)
		MonHex(de18:1/16:0)	MonHex(de18:1/18:0)	MonHex(de18:1/24:1)	MonHex(de18:1/26:0)
			MonHex(de18:1/20:0)	MonHex(de18:1/24:0)	
	**Phosphosphingolipid**	SM(de18:1/14:0)	SM(de18:1/18:1)	SM(de18:1/22:0)	SM(de18:1/26:1)
		SM(de18:1/16:0)	SM(de18:1/18:0)	SM(de18:1/24:1)	SM(de18:1/26:0)
			SM(de18:1/20:0)	SM(de18:1/24:0)	
	**Fatty acid**	AA	DHA	EPA	DHGLA
**Fatty Acyls**	**Eicosanoids**	6keto-PGF1α		8,9-EET	(±) 14-15-DHET
		PGF_2_α		14,15-EET	(±) 8,9-DHET
		PGE_2_		PGA_2_	(±) 11,12-DHET
		PGD_2_		8-iso PGF_2_	TXB_2_
		5-HETE		RVD_1_	RVD_2_
		12-HETE		LTE4	RVD_3_
		15-HETE		LTB4	LXA4
		20-HETE		LTC4	5-iPF2α-VI
		PGE_1_		LTD4	Maresin 2

Lipid categories are subdivided into sphingolipids, fatty acyls, and eicosanoids, and further subdivided into respective lipid classes as shown.

Eicosanoids were separated using a Shimadzu Nexera X2 LC-30AD coupled to a SIL-30AC auto injector, coupled to a DGU-20A5R degassing unit in the following way. A 14 min, reversed phase LC method utilizing an Ascentis Express C18 column (150 mm × 2.1 mm, 2.7 μm) was used to separate the eicosanoids at a 0.5 ml/min flow rate at 40°C. The column was equilibrated with 100% solvent A [acetonitrile:water:formic acid (20:80:0.02, v/v/v)] for 5 min and then 10 μl of sample was injected. Hundred percent solvent A was used for the first 2 min of elution. Solvent B [acetonitrile:isopropanol:formic acid (20:80:0.02, v/v/v)] was increased in a linear gradient to 25% solvent B at 3 min, to 30% at 6 min, to 55% at 6.1 min, to 70% at 10 min, and to 100% at 10.10 min. Hundred percent solvent B was held constant until 13.0 min, where it was decreased to 0% solvent B and 100% solvent A from 13.0 min to 13.1 min. From 13.1 min to 14.0 min, solvent A was held constant at 100%.

Eicosanoids were analyzed via mass spectrometric means using an AB Sciex Triple Quad 5500 Mass Spectrometer. Q1 and Q3 were set to detect distinctive precursor and product ion pairs. Ions were fragmented in Q2 using N_2_ gas for collisionally induced dissociation. Analysis used multiple-reaction monitoring in negative-ion mode. Eicosanoids were monitored using precursor → product multiple reaction monitoring (MRM) pairs. The mass spectrometer parameters used were as follows: curtain gas, 20 psi; collisionally activated dissociation, medium; ion spray voltage, −4,500 V; temperature, 300°c; gas 1, 40 psi; gas 2, 60 psi; declustering potential, collision energy, and Cell Exit Potential vary per transition (supplemental Table S1).

#### Analysis of sphingolipids by UPLC ESI-MS/MS

Plasma (50 μl) was extracted using a modified Bligh Dyer Extraction and analyzed by UPLC ESI-MS/MS as previously described by us and others (52, 57, 59, 60, 61) (Table 2). Samples were spiked with 250 pmol of ceramide-1-phosphates (C1P), SM, CER, and monohexosyl ceramide (MonHex) (de18:1/12:0 species), and So, sphinganine, S1P, sphinganine-1-phosphate (d17:0 sphinganine/d17:1 So) as internal standards (Avanti Polar Lipids). Following addition of internal standards, MeOH:CHCl_3_ (2:1) was added to the plasma, and the mixture was sonicated to disperse plasma clumps. Samples were then incubated for 6 h at 48°C. Extracts were then centrifuged at 5,000 rpm for 20 min, transferred to a new glass tube, dried down, and reconstituted in methanol (500 μl) by sonicating. Extracts were again centrifuged at 5,000 rpm for 20 min and transferred to injection vials for mass spectrometric analysis.

Sphingolipids were separated using a Shimadzu Nexera X2 LC-30AD coupled to a SIL-30AC auto injector, coupled to a DGU-20A5R degassing unit in the following way. An 8 min, reversed phase LC method utilizing an Ascentis Express C18 column (5 cm x 2.1 mm, 2.7 μm) was used to separate the eicosanoids at a 0.5 ml/min flow rate at 60°C. The column was equilibrated with 100% solvent A [methanol:water:formic acid (58:44:1, v/v/v) with 5 mM ammonium formate] for 5 min and then 10 μl of sample was injected. Hundred percent solvent A was used for the first 0.5 min of elution. Solvent B [methanol:formic acid (99:1, v/v) with 5 mM ammonium formate] was increased in a linear gradient to 100% solvent B from 0.5 min to 3.5 min. Solvent B was held constant at 100% from 3.5 min to 6 min. From 6 min to 6.1 min, solvent B was reduced to 0%, and solvent A returned to 100%. Solvent A was held constant at 100% from 6.1 min to 8 min.

Sphingolipids were analyzed via mass spectrometric means using an AB Sciex Triple Quad 5500 Mass Spectrometer. Q1 and Q3 were set to detect distinctive precursor and product ion pairs. Ions were fragmented in Q2 using N2 gas for collisionally induced dissociation. Analysis used MRM in positive-ion mode. Sphingolipids were monitored using precursor → product MRM pairs. The mass spectrometer parameters used were as follows: curtain gas, 30 psi; collisionally activated dissociation, medium; ion spray voltage, 5,500 V; temperature, 500°c; gas 1, 60 psi; gas 2, 40 psi; declustering potential, collision energy, and Cell Exit Potential vary per transition (supplemental Table S2).

### Statistical analysis

Raw data generated via UPLC ESI-MS/MS were evaluated and plotted using the R statistical computing program [R version 4.1.0 (2021-05-18) -- "Camp Pontanezen"]. Subject data that had a value ≤0.0001 for any specific lipid were excluded due to being below the level of detection via the UPLC ESI-MS/MS method used. For violin plots portrayed in the figures, raw data were log transformed to promote normality and differences between the two groups and then examined using Students *t* test with Welch’s correction. These data were analyzed for proper data distribution by the Shapiro-Wilk Test. Some of the group data failed this test (designated by a $ in Figures and Tables), and thus, nontransformed data were also analyzed by the Wilcoxon Sum Rank Test. For depicted heat maps, data were scaled and then plotted. All data are reported as mean ± SD, and a *P* value < 0.05 was considered statistically significant in both statistical analyses employed. Only those bioactive lipid mediators found statistically significant in a group comparison by both methods are discussed in the results section.

## Results

### Characteristics of the study subjects

Demographic data for 31 normal pregnant and 26 preeclamptic subjects are presented in Table 1. Maternal age and BMI were matched, and the PE group showed the significant elevation in systolic and diastolic blood pressures that accompany the disease, as well as proteinuria. Additional characteristics of the study subjects are summarized as follows: women of self-designated European ancestry (Caucasian/White) constituting 21% (12 of 57) of the subjects, women of self-designated African-descent (Black) constituted 58% (33 of 57) of the subjects, and women of self-designated Hispanic origin constituted 21% (12 of 57) of the subjects. Furthermore, BMI >30, smoking status, and illicit drug use was determined and reported in Table 1. Women with a high BMI constituted 28.1% (16 of 57) of the subjects, but they contributed 46% of the cases of PE. Women with a history of smoking constituted 33.3% (19 of 57) of the subjects and they represented 26.9% of the cases of PE. Women with a history of illicit drug use made up 8.8% of the subjects (5 of 57) but 3.8% of the cases of PE (1 of 26). Of the PE cases, 12% or 46.1% of our PE subjects had a preterm birth (birth occurred before 37 weeks), and 14% or 53.9% of our PE subjects had a term birth (birth occurred at or after 37 weeks). Of the PE cases, 18 of 26 subjects (69.2%) had PE with severe features, and 8 of 26 subjects (30.8%) were classified as mild PE. Of the pregnancies, 36 of the 57 subjects were recruited from high-risk OB/GYN clinic at Virginia Commonwealth University Medical Center and were prescribed low-dose aspirin therapy (81mg/d).

### Eicosanoids and sphingolipids are early predictors of severe PE

**Fig. 1:**
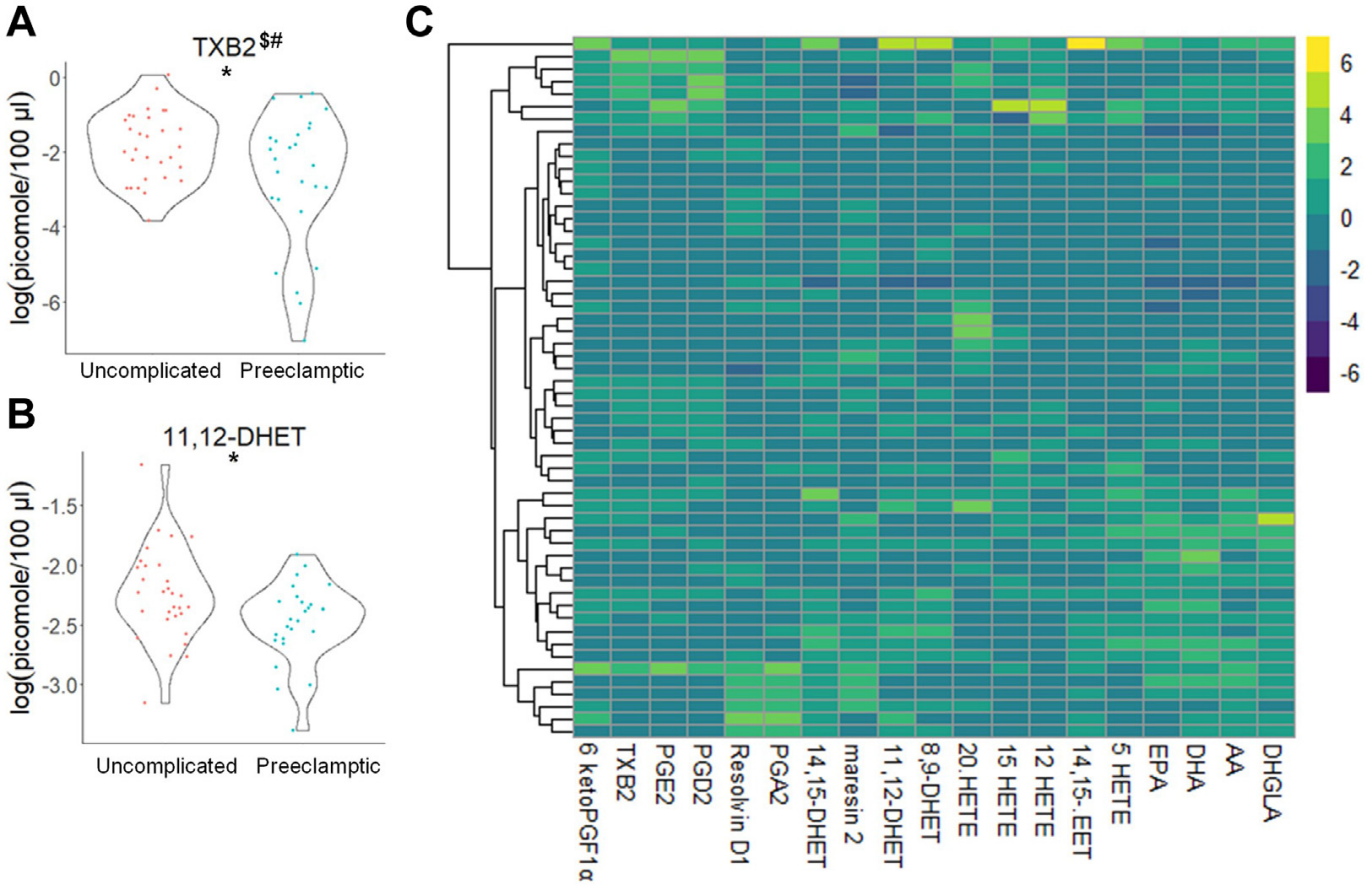
Distinct eicosanoids show significant differences in the plasma from uncomplicated term pregnant patients versus total PE pregnancies. A and B: Eicosanoid species that occurred at significantly different levels when comparing plasma from patients with normal, uncomplicated pregnancies versus patients later diagnosed with PE using UPLC ESI-MS/MS as the detection method. Samples were analyzed by UPLC ESI-MS/MS within two weeks of acquisition. C: Heat map of all eicosanoid species that were detected via UPLC ESI-MS/MS in plasma (depicted as fold change). Samples were compared using unpaired students *t* test with Welch’s correction. Data are means ± SD depicted as violin plots, ∗*P* < 0.05. The log-transformed data failing the Shapiro-Wilk Test are designated with a $. Nontransformed data were also analyzed by the Wilcoxon Sum Rank Test. Bioactive lipid mediators not found to be significantly different by the Wilcoxon Sum Rank Test are designated with a #. PE, preeclampsia; UPLC ultra-performance liquid chromatography.

**Fig. 2:**
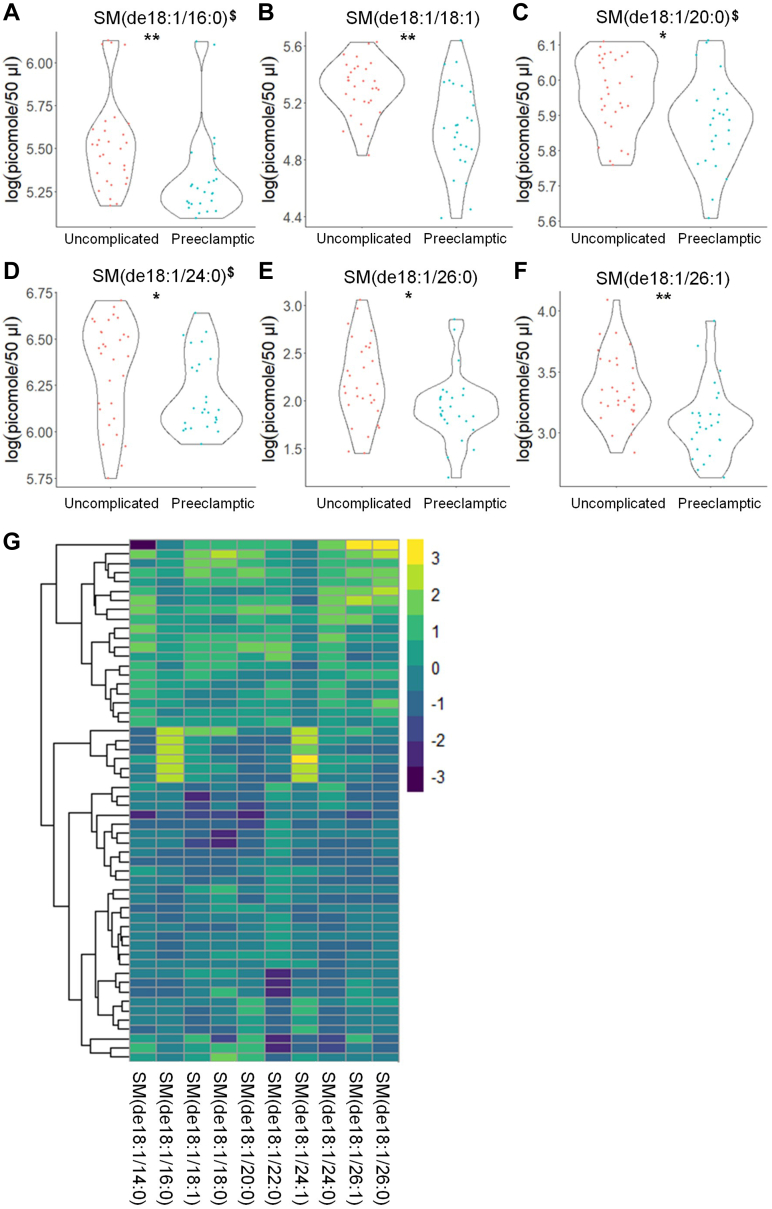
Sphingomyelin levels show significant differences in the plasma from uncomplicated term pregnant patients versus total PE pregnancies. A–F: Sphingomyelin species that occurred at significantly different levels when comparing plasma from patients with normal, uncomplicated pregnancies versus patients later diagnosed with PE using UPLC ESI-MS/MS as the detection method. Samples were analyzed by UPLC ESI-MS/MS within two weeks of acquisition. G: Heat map of all sphingomyelin species that were detected via UPLC ESI-MS/MS in plasma (depicted as fold change). Samples were compared using unpaired students *t* test with Welch’s correction. Data shown are means ± SD depicted as violin plots, ∗*P* < 0.05, ∗∗*P* < 0.01. The log-transformed data failing the Shapiro-Wilk Test are designated with a $. PE, preeclampsia; UPLC ultra-performance liquid chromatography.

**Fig. 3:**
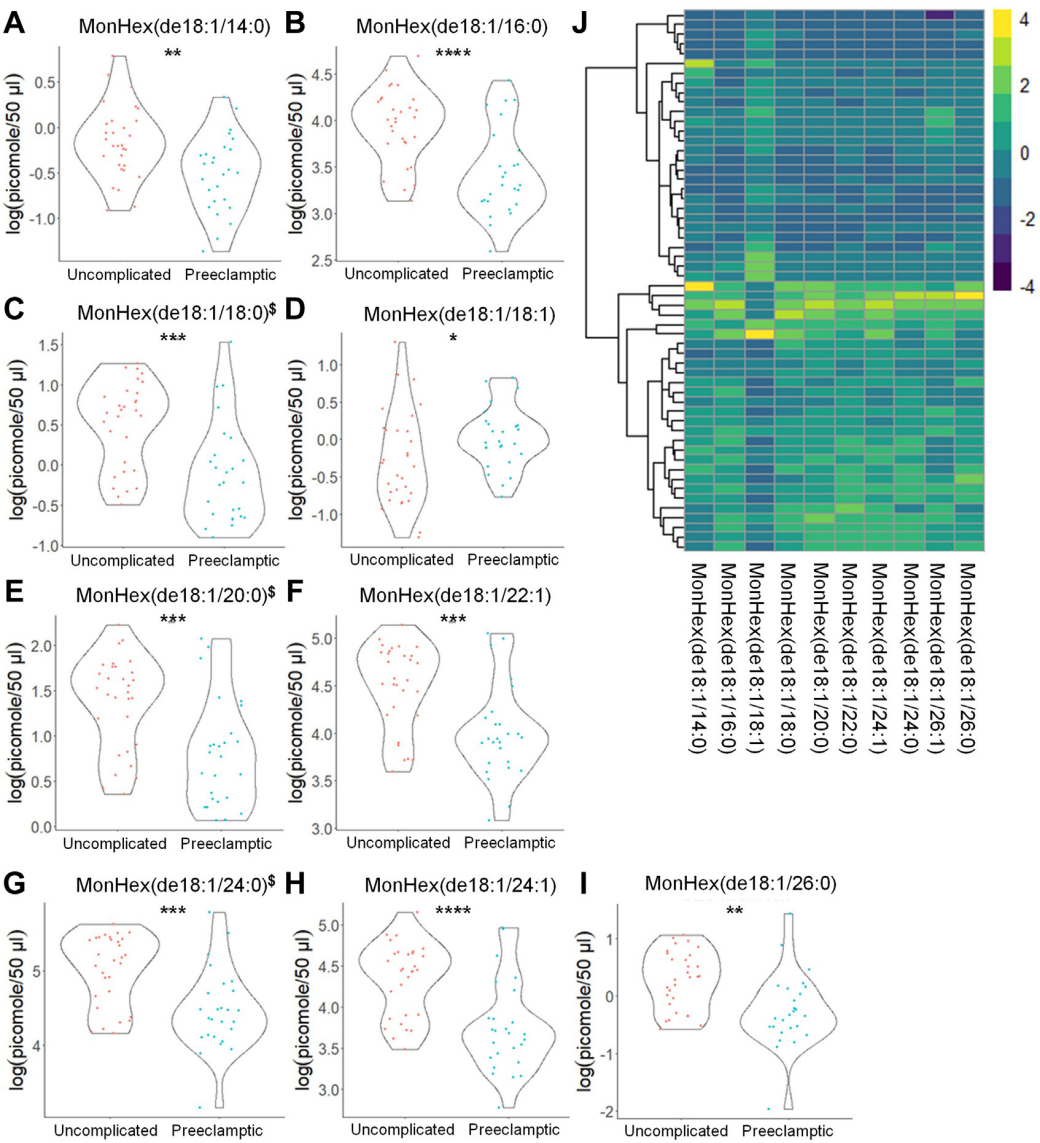
MonHex species show significant differences in the plasma from uncomplicated term pregnant patients versus total PE pregnancies. A–I: MonHex species that occurred at significantly different levels when comparing plasma from patients with normal, uncomplicated pregnancies versus patients later diagnosed with PE using UPLC ESI-MS/MS as the detection method. Samples were analyzed by UPLC ESI-MS/MS within two weeks of acquisition. J: Heat map of all MonHex species that were detected via UPLC ESI-MS/MS in plasma (depicted as fold change). Samples were compared using unpaired students *t* test with Welch’s correction. Data shown are means ± SD depicted as violin plots, ∗*P* < 0.05, ∗∗*P* < 0.01, ∗∗∗*P* < 0.001, ∗∗∗∗*P* < 0.0001. The log-transformed data failing the Shapiro-Wilk Test are designated with a $. MonHex, monohexosylceramides; PE, preeclampsia; UPLC ultra-performance liquid chromatography.

**Fig. 4:**
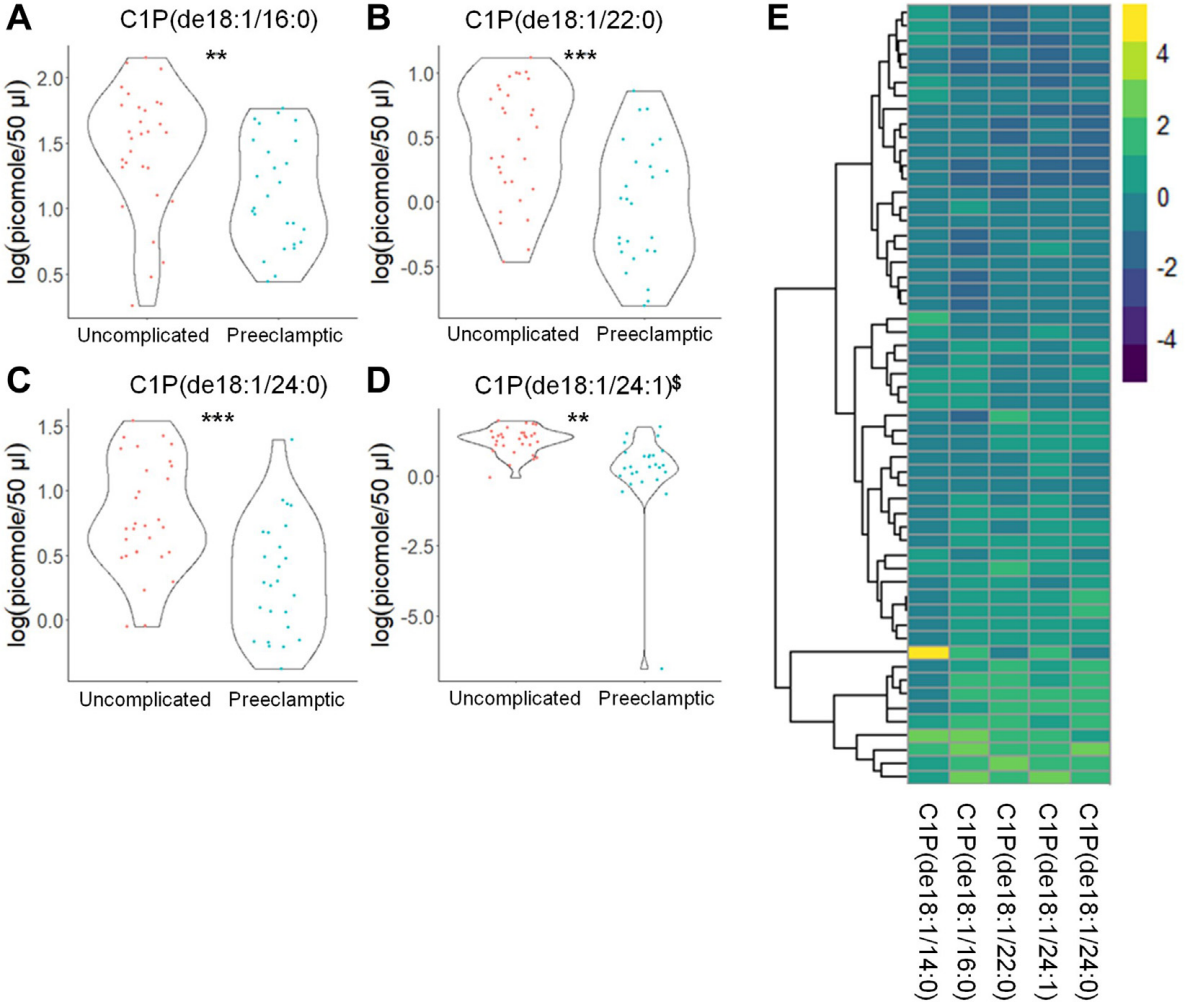
C1P levels show significant differences in the plasma from uncomplicated term pregnant patients versus total PE pregnancies. A–D: C1P species that occurred at significantly different levels when comparing plasma from patients with normal, uncomplicated pregnancies versus patients later diagnosed with PE using UPLC ESI-MS/MS as the detection method. Samples were analyzed by UPLC ESI-MS/MS within two weeks of acquisition. E: Heat map of all C1P species that were detected via UPLC ESI-MS/MS in plasma (depicted as fold change). Samples were compared using unpaired students *t* test with Welch’s correction. Data shown are means ± SD depicted as violin plots, ∗∗*P* < 0.01, ∗∗∗*P* < 0.001. The log-transformed data failing the Shapiro-Wilk Test are designated with a $. C1P, ceramide-1-phosphate; PE, preeclampsia; UPLC ultra-performance liquid chromatography.

**Fig. 5:**
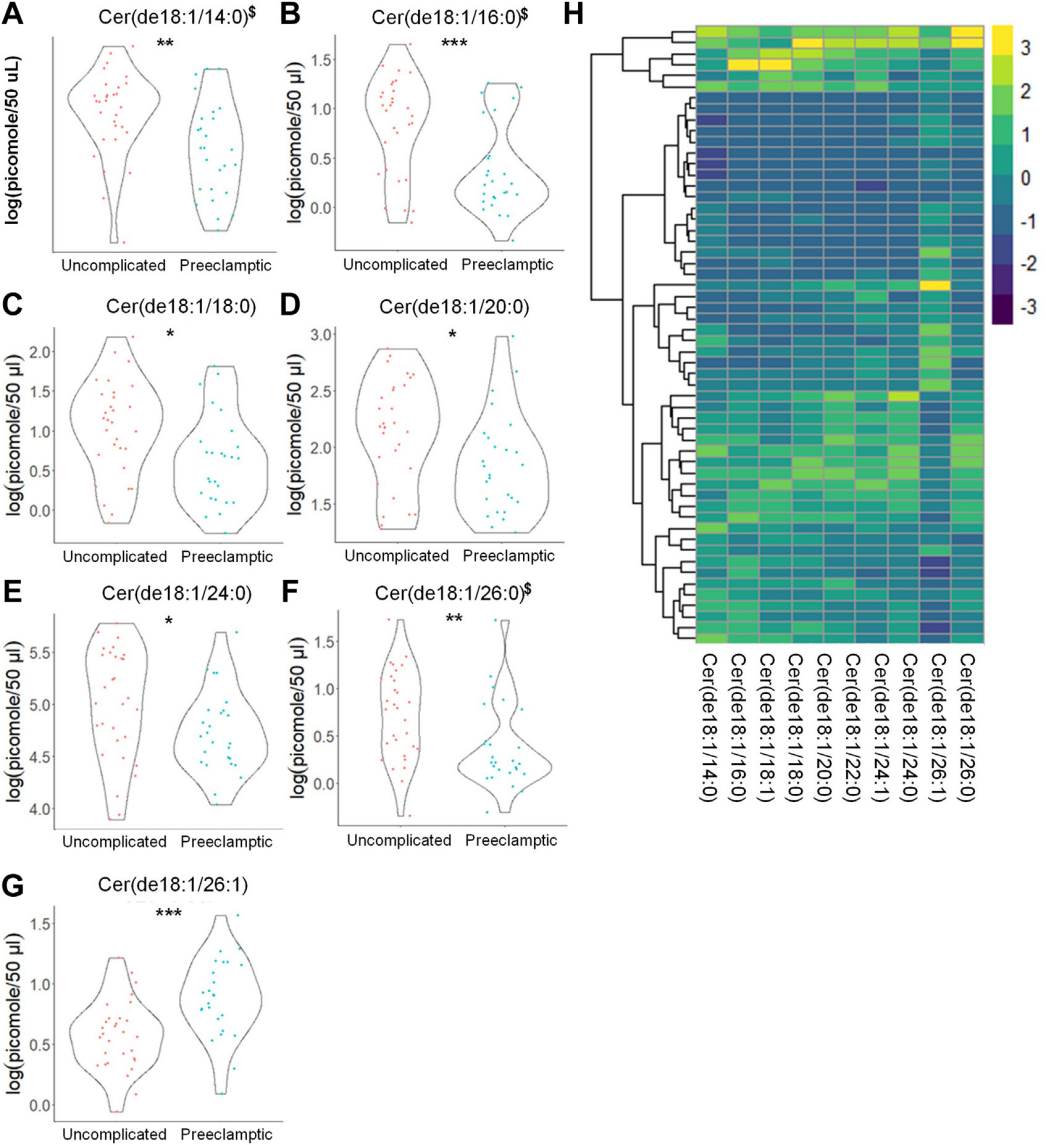
Ceramide species show significant differences in the plasma from uncomplicated term pregnant patients versus total PE pregnancies. A–G: Ceramide species that occurred at significantly different levels when comparing plasma from patients with normal, uncomplicated pregnancies versus patients later diagnosed with PE using UPLC ESI-MS/MS as the detection method. Samples were analyzed by UPLC ESI-MS/MS within two weeks of acquisition. H: Heat map of all ceramide species that were detected via UPLC ESI-MS/MS in plasma (depicted as fold change). Samples were compared using unpaired students *t* test with Welch’s correction. Data shown are means ± SD depicted as violin plots, ∗*P* < 0.05, ∗∗*P* < 0.01, ∗∗∗*P* < 0.001. The log-transformed data failing the Shapiro-Wilk Test are designated with a $. PE, preeclampsia; UPLC ultra-performance liquid chromatography.

Targeted lipidomic analysis revealed significant differences by two different statistical analyses in the eicosanoid, (±)11,12 DHET (decreased), prior to 24 weeks of gestation, which were associated with the later development of PE (31 uncomplicated, non-PE pregnancies vs. 26 PE pregnancies) (Fig. 1) (Tables 3 and 4) (supplemental Table S3). Targeted lipidomic analysis also demonstrated significant differences in CERs, SMs, C1P, and MonHex of the following chain lengths: CER: de_18:1/14:0_, de_18:1/16:0_, de_18:1/18:0_, de_18:1/20:0_, de_18:1/24:0_, de_18:1/26:0_ (all decreased), and de_18:1/26:1_ (increased); C1P (all decreased): de_18:1/16:0,_ de_18:1/22:0_, de_18:1/24:0_, and de_18:1/24:1_; SM (all decreased): de_18:1/16:0_, de_18:1/18:1_, de_18:1/20:0_, de_18:1/24:0_, de_18:1/26:0_, and de_18:1/26:1_; MonHex (all decreased, but de_18:1/18:1_): de_18:1/14:0,_ de_18:1/16:0_, de_18:1/18:0_, de_18:1/18:1,_ de_18:1/20:0_, de_18:1/22:0_, de_18:1/24:0_, de_18:1/24:1_, and de_18:1/26:0_ prior to 24 weeks of gestation, which were associated with the later development of PE (Fig. 2, Fig. 3, Fig. 4, Fig. 5) (Tables 5 and 6) (supplemental Table S4). These changes in plasma lipid levels were mainly indicative of PE with severe features as mild PE patients (8 total PE patients) showed only a modest decrease in PGA_2_ and one SM species (de_18:1/16:0_ SM) (data not shown). Each lipid was determined to be an independent predictor of future PE onset with a *P* ≤ 0.05 using two separate statistical analyses. Therefore, these data show that the levels of specific eicosanoids and sphingolipids are significantly different at early gestational times between subjects with normal pregnancies and subjects subsequently presenting with PE with severe features.

**Table 3:** Raw eicosanoid profile observed in subjects with uncomplicated term and PE pregnancies

Analyte	Uncomplicated (n = 31) (pmol/100 μl Plasma)	PE (n = 26) (pmol/100 μl Plasma)
6-Keto PGF1α	0.008 ± 0.004	0.007 ± 0.002
TXB_2_	0.231 ± 0.219	0.169 ± 0.186
PGE_2_	0.027 ± 0.025	0.018 ± 0.013
PGD_2_	0.030 ± 0.025	0.037 ± 0.034
Resolvin D1	0.126 ± 0.015	0.131 ± 0.015
PGA_2_	0.232 ± 0.024	0.232 ± 0.020
(±)14,15 DHET	0.109 ± 0.042	0.092 ± 0.029
Maresin 2	0.009 ± 0.004	0.010 ± 0.005
(±)11,12 DHET	0.115 ± 0.050	0.088 ± 0.026
(±) 8,9 DHET	0.030 ± 0.014	0.025 ± 0.007
20-HETE	9.396 ± 4.891	9.394 ± 3.168
15-HETE	0.121 ± 0.084	0.079 ± 0.032
12-HETE	0.682 ± 0.932	0.405 ± 0.447
(±)14,15 EET	0.033 ± 0.045	0.023 ± 0.012
5-HETE	0.174 ± 0.123	0.131 ± 0.078
EPA	15.347 ± 6.879	15.419 ± 9.066
DHA	121.261 ± 46.190	134.094 ± 61.682
AA	473.159 ± 217.916	469.597 ± 223.417
DHGLA	80.793 ± 36.344	84.143 ± 59.805

Raw eicosanoid levels observed in plasma from patients with uncomplicated pregnancies versus patients later diagnosed with PE. Samples were taken prior to 24 weeks gestation and analyzed by UPLC ESI-M/S within 2 weeks of acquisition. The data presented are means ± SD in pmol lipid/100 μl plasma.

**Table 4:** Log-normalized eicosanoid profile observed in subjects with uncomplicated term and PE pregnancies

Analyte	Uncomplicated (n = 31) log(pmol/100 μl Plasma)	PE (n = 26) log(pmol/100 μl Plasma)
6-Keto PGF1α	−4.978 ± 0.489	−5.072 ± 0.396
TXB_2_	−1.852 ± 0.918	−2.717 ± 1.801**∗**^**$#**^
PGE_2_	−4.016 ± 1.083	−4.285 ± 0.781
PGD_2_	−3.874 ± 0.932	−3.737 ± 1.026
Resolvin D1	−2.077 ± 0.107^$^	−2.041 ± 0.11
PGA_2_	−1.465 ± 0.098	−1.464 ± 0.082
(±)14,15 DHET	−2.272 ± 0.337^$^	−2.438 ± 0.362
Maresin 2	−4.813 ± 0.417	−4.757 ± 0.524
(±)11,12 DHET	−2.243 ± 0.387	−2.476 ± 0.329**∗**
(±) 8,9 DHET	−3.581 ± 0.39	−3.718 ± 0.317
20-HETE	2.133 ± 0.445^$^	2.187 ± 0.33
15-HETE	−2.399 ± 1.034	−2.616 ± 0.395
12-HETE	−0.984 ± 1.074	−1.456 ± 1.11
(±)14,15 EET	−3.732 ± 0.676	−3.868 ± 0.488
5-HETE	−1.973 ± 0.672	−2.186 ± 0.555
EPA	2.609 ± 0.538	2.524 ± 0.713
DHA	4.719 ± 0.415	4.785 ± 0.502
AA	6.056 ± 0.466	6.026 ± 0.531
DHGLA	4.281 ± 0.498	4.204 ± 0.693

Log-normalized eicosanoid levels observed in patients with uncomplicated pregnancies versus patients later diagnosed with PE. Samples were taken prior to 24 weeks gestation and analyzed by UPLC ESI-M/S within 2 weeks of acquisition. Raw data were log transformed to promote normality and differences between the two groups and then compared using unpaired students *t* test with Welch’s correction. The data presented are in means ± SD in log(pmol lipid/100 μl plasma). Significance is represented as ∗*P* < 0.05. The log-transformed data failing the Shapiro-Wilk Test are designated with a $. Nontransformed data were also analyzed by the Wilcoxon Sum Rank Test. Bioactive lipid mediators not found to be significantly different by the Wilcoxon Sum Rank Test are designated with a #.

**Table 5:** Raw sphingolipid profile observed in subjects with uncomplicated term and PE pregnancies

Analyte	Uncomplicated (n = 31) (pmol/50 μl Plasma)	PE (n = 26) (pmol/50 μl Plasma)
Cer(de18:1/14:0)	0.711 ± 0.223	0.530 ± 0.216
Cer(de18:1/16:0)	2.569 ± 1.080	1.622 ± 0.831
Cer(de18:1/18:0)	3.305 ± 1.891	2.203 ± 1.419
Cer(de18:1/20:0)	9.335 ± 3.983	7.007 ± 3.666
Cer(de18:1/22:0)	63.652 ± 29.611	47.675 ± 22.264
Cer(de18:1/24:1)	47.217 ± 16.042	42.753 ± 19.247
Cer(de18:1/24:0)	168.034 ± 77.278	121.972 ± 52.833
Cer(de18:1/26:1)	1.839 ± 0.536	2.530 ± 0.789
Cer(de18:1/26:0)	2.275 ± 1.063	1.624 ± 0.987
C1P(de18:1/14:0)	0.921 ± 0.391	0.652 ± 0.269
C1P(de18:1/16:0)	55.621 ± 20.155	34.717 ± 18.148
C1P(de18:1/22:0)	0.951 ± 0.726	1.107 ± 0.467
C1P(de18:1/24:1)	1.876 ± 0.868	1.115 ± 0.929
C1P(de18:1/24:0)	4.421 ± 1.942	2.681 ± 1.850
SM(de18:1/14:0)	97.654 ± 36.269	61.833 ± 34.830
SM(de18:1/16:0)	83.263 ± 33.211	49.406 ± 32.552
SM(de18:1/18:1)	165.409 ± 64.728	100.689 ± 63.499
SM(de18:1/18:0)	1.466 ± 0.318	1.302 ± 0.466
SM(de18:1/20:0)	1.485 ± 0.691	0.957 ± 0.786
SM(de18:1/22:0)	287.493 ± 35.351	275.209 ± 32.059
SM(de18:1/24:1)	262.125 ± 81.104	215.547 ± 72.705
SM(de18:1/24:0)	205.154 ± 36.712	160.882 ± 49.253
SM(de18:1/26:1)	302.781 ± 47.680	280.039 ± 51.468
SM(de18:1/26:0)	390.526 ± 39.575	360.336 ± 44.657
MonHex(de18:1/14:0)	539.834 ± 91.772	531.642 ± 74.826
MonHex(de18:1/16:0)	546.530 ± 115.308	500.887 ± 110.969
MonHex(de18:1/18:1)	584.941 ± 144.885	503.640 ± 107.065
MonHex(de18:1/18:0)	30.257 ± 9.112	23.247 ± 7.976
MonHex(de18:1/20:0)	9.851 ± 4.287	7.401 ± 3.048
MonHex(de18:1/22:0)	0.233 ± 0.148	0.176 ± 0.052
MonHex(de18:1/24:1)	4.691 ± 1.869	3.312 ± 1.336
MonHex(de18:1/24:0)	1.748 ± 0.705	1.086 ± 0.521
MonHex(de18:1/26:1)	3.798 ± 1.502	1.780 ± 1.282
MonHex(de18:1/26:0)	2.432 ± 1.014	1.540 ± 0.734
de18:1 So	1.459 ± 1.302	1.229 ± 0.746
de18:1 So1P	44.116 ± 17.025	41.853 ± 12.541
de18:0 Sa1P	7.212 ± 3.123	6.780 ± 2.274

Raw sphingolipid levels observed in plasma from patients with uncomplicated pregnancies versus patients later diagnosed with PE. Samples were taken prior to 24 weeks gestation and analyzed by UPLC ESI-M/S within 2 weeks of acquisition. The data presented are means ± SD in pmol lipid/50 μl plasma.

**Table 6:** Log-normalized sphingolipid profile observed in subjects with uncomplicated term and PE pregnancies

Analyte	Uncomplicated (n = 31) log(pmol/50 μl Plasma)	PE (n = 26) log(pmol/50 μl Plasma)
Cer(de18:1/14:0)	−0.4 ± 0.37^$^	−0.715 ± 0.408∗∗
Cer(de18:1/16:0)	0.838 ± 0.493^$^	0.375 ± 0.452**∗∗∗**^**$**^
Cer(de18:1/18:0)	1.029 ± 0.605	0.622 ± 0.567**∗**
Cer(de18:1/20:0)	2.132 ± 0.476	1.843 ± 0.44**∗**
Cer(de18:1/22:0)	4.023 ± 0.547	3.778 ± 0.404
Cer(de18:1/24:1)	3.788 ± 0.387	3.664 ± 0.428
Cer(de18:1/24:0)	5 ± 0.535	4.726 ± 0.389**∗**
Cer(de18:1/26:1)	0.57 ± 0.285	0.879 ± 0.324**∗∗∗**
Cer(de18:1/26:0)	0.715 ± 0.475	0.369 ± 0.446**∗∗**^**$**^
C1P(de18:1/14:0)	−1.592 ± 0.5	−1.773 ± 0.265
C1P(de18:1/16:0)	1.45 ± 0.476	1.116 ± 0.413**∗∗**
C1P(de18:1/22:0)	0.466 ± 0.453	−0.028 ± 0.479**∗∗∗**
C1P(de18:1/24:1)	1.242 ± 0.464	0.145 ± 1.552**∗∗**
C1P(de18:1/24:0)	0.801 ± 0.432	0.334 ± 0.444**∗∗∗**^**$**^
SM(de18:1/14:0)	5.653 ± 0.135	5.611 ± 0.117
SM(de18:1/16:0)	5.529 ± 0.273^$^	5.334 ± 0.261**∗∗**^**$**^
SM(de18:1/18:1)	5.307 ± 0.19	5.034 ± 0.316**∗∗**
SM(de18:1/18:0)	5.7 ± 0.171	5.618 ± 0.189^#^
SM(de18:1/20:0)	5.962 ± 0.106^$^	5.879 ± 0.126**∗**
SM(de18:1/22:0)	6.275 ± 0.193	6.265 ± 0.159
SM(de18:1/24:1)	6.284 ± 0.193	6.197 ± 0.193^#^
SM(de18:1/24:0)	6.337 ± 0.275^$^	6.201 ± 0.203**∗**^**$**^
SM(de18:1/26:1)	3.369 ± 0.282	3.099 ± 0.3**∗∗**
SM(de18:1/26:0)	2.199 ± 0.427	1.935 ± 0.358**∗**
MonHex(de18:1/14:0)	−0.162 ± 0.399	−0.51 ± 0.417**∗∗**
MonHex(de18:1/16:0)	3.949 ± 0.39	3.433 ± 0.47**∗∗∗∗**
MonHex(de18:1/18:1)	−0.264 ± 0.634	0.021 ± 0.407**∗**
MonHex(de18:1/18:0)	0.501 ± 0.542^$^	−0.117 ± 0.628**∗∗∗**^**$**^
MonHex(de18:1/20:0)	1.372 ± 0.513^$^	0.804 ± 0.583**∗∗∗**^**$**^
MonHex(de18:1/22:0)	4.497 ± 0.444	3.999 ± 0.489**∗∗∗**
MonHex(de18:1/24:1)	4.334 ± 0.441	3.74 ± 0.543**∗∗∗∗**
MonHex(de18:1/24:0)	5.014 ± 0.468^$^	4.462 ± 0.538^#^
MonHex(de18:1/26:1)	0.359 ± 0.227	0.192 ± 0.411
MonHex(de18:1/26:0)	0.279 ± 0.502	−0.258 ± 0.637**∗∗**
de18:1 So	0.068 ± 0.79	0.053 ± 0.55
de18:1 So1P	3.709 ± 0.411	3.69 ± 0.309
de18:0 Sa1P	1.863 ± 0.527^$^	1.852 ± 0.374

Log-normalized sphingolipid levels observed in patients with uncomplicated pregnancies versus patients later diagnosed with PE. Samples were taken prior to 24 weeks gestation and analyzed by UPLC ESI-M/S within 2 weeks of acquisition. Samples were compared using unpaired students *t* test with Welch’s correction. The data presented are in means ± SD in log(pmol lipid/50 μl plasma). Significance is represented as ∗ *P* < 0.05; ∗∗*P* < 0.01; ∗∗∗*P* < 0.001; ∗∗∗∗*P* < 0.0001. The log-transformed data failing the Shapiro-Wilk Test are designated with a $. Nontransformed data were also analyzed by the Wilcoxon Sum Rank Test. Bioactive lipid mediators not found to be significantly different by the Wilcoxon Sum Rank Test are designated with a #.

### Eicosanoids stratify PE patients into preterm and term births

**Fig. 6:**
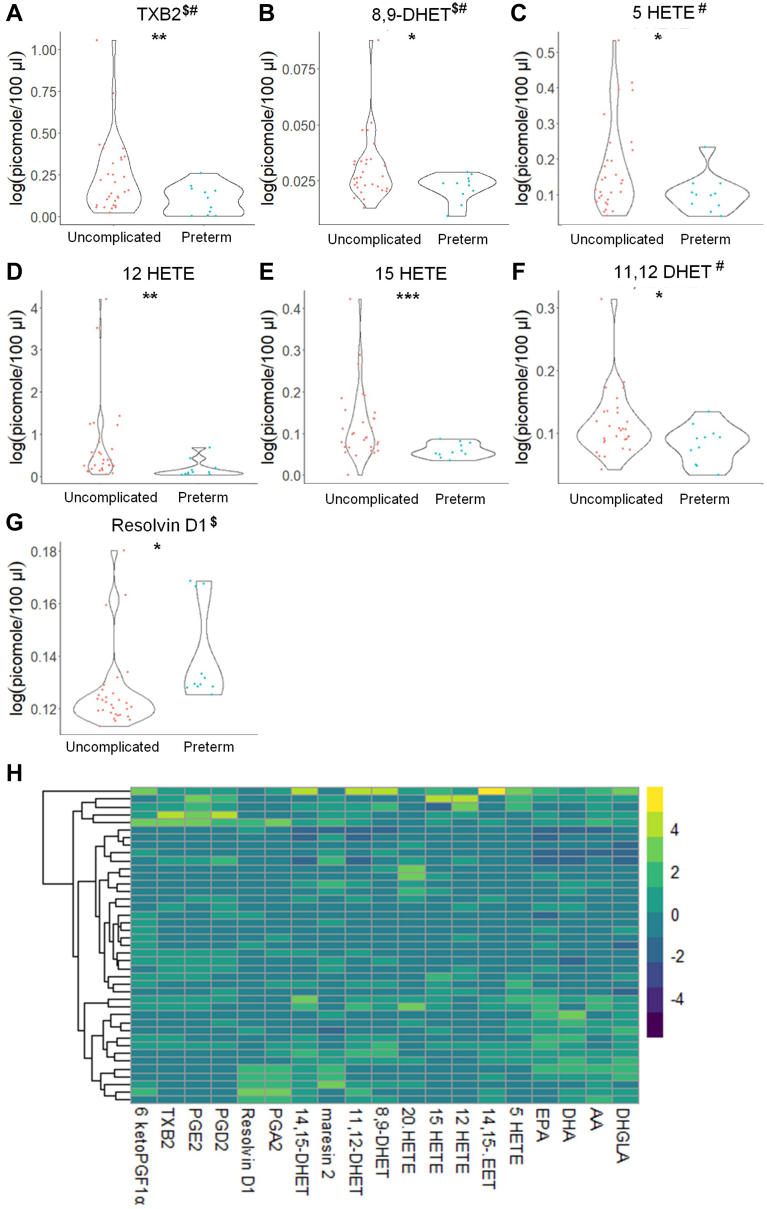
Distinct eicosanoids show significant differences in the plasma from uncomplicated term pregnant patients versus PE pregnancies with a preterm birth. A–G: Eicosanoid species that occurred at significantly different levels when comparing plasma from uncomplicated term pregnant patients versus PE pregnancies with a preterm birth using UPLC ESI-MS/MS as the detection method. Samples were analyzed by UPLC ESI-MS/MS within two weeks of acquisition. H: Heat map of all eicosanoid species that were detected via UPLC ESI-MS/MS in plasma (fold change is depicted). Samples were compared using unpaired students *t* test with Welch’s correction. Data shown are means ± SD depicted as violin plots, ∗*P* < 0.05, ∗∗*P* < 0.01, ∗∗∗*P* < 0.001. The log-transformed data failing the Shapiro-Wilk Test are designated with a $. Nontransformed data were also analyzed by the Wilcoxon Sum Rank Test. Bioactive lipid mediators not found to be significantly different by the Wilcoxon Sum Rank Test are designated with a #. PE, preeclampsia; UPLC ultra-performance liquid chromatography.

To determine whether our observed changes in bioactive lipids were consistent regardless of preterm birth, we compared control, uncomplicated pregnancies (31 patients) versus PE patients with preterm (birth occurred prior to 37 weeks) or term births. In contrast to the nonstratified PE comparisons, patients later diagnosed with PE, but also had a preterm birth, showed decreased plasma levels of 12-HETE and 15-HETE with RvD1 levels observed to be increased (Fig. 6; supplemental Table S5). In PE subjects with a term birth (≥37 weeks), no significant differences were observed for eicosanoids (data not shown and supplemental Table S6). In regard to sphingolipids, both PE groups demonstrated similar reductions in various sphingolipid species of SM, CER, C1P, and MonHex as observed in the nonstratified comparison (supplemental Figs. S1–S8; supplemental Tables S7–S8), but a few significant differences were found between the two groups. For example, PE patients with a preterm birth showed a decrease in the levels of de_18:1/C14:0_ CER, this was not observed in the PE patients with a term birth. Regardless of the gestational age at delivery, de_18:1/26:1_ CER was increased in patients subsequently diagnosed with PE. These data demonstrate that the levels of specific eicosanoids and sphingolipids can stratify PE patients based on whether the birth will be preterm or term, but de_18:1/26:1_ CER is a common marker of future PE development regardless of birth subtype.

### Eicosanoid differences are linked to PE development in specific, self-determined racial backgrounds

To determine whether the plasma levels of bioactive lipids were associated with self-reported racial backgrounds, we compared control and PE pregnancies of the same self-determined racial background. For women of self-designated African-descent (Black), the lipid profile of PE patients was similar to the nonracially stratified data, although TXB_2_ levels were now significantly lower in PE pregnancies and (±)11,12 DHET as well as several sphingolipid species were no longer significantly different between the groups (supplemental Figs. S9–S13; supplemental Tables S9 and S10). Decreased levels of 12-HETE, 15-HETE, EPA, and DHA in the plasma of self-designated Caucasian patients were observed in contrast to the nonstratified analyses. Furthermore, a number of key sphingolipid-based markers of PE development in the plasma were no longer significantly different such as hexosylceramides and CERs observed in the nonracially stratified analyses (supplemental Figs. S14–S16; supplemental Tables S11 and S12). Patients of self-designated Hispanic background demonstrated a significant and novel increase in PGA_2_ levels linked to future PE development that was not observed for patients of self-designated Caucasian or Black ancestry (supplemental Fig. S17; supplemental Table S13). Patients of Hispanic origin also did not have differences in the plasma levels of CERs as well as many species of other sphingolipid classes in regard to later PE diagnosis (supplemental Figs. S18–S20; supplemental Table S14). Interestingly, Hispanic patients also showed a substantially higher rate of PE development (75%) compared to self-designated Caucasian (41.6%) and Black (39.4%) subjects. These data demonstrate that the levels of specific eicosanoids and sphingolipid species are, in some cases, predictive of later PE development and linked to self-determined ancestry.

### Eicosanoids and sphingolipids differ in high-risk pregnant patients

**Fig. 7:**
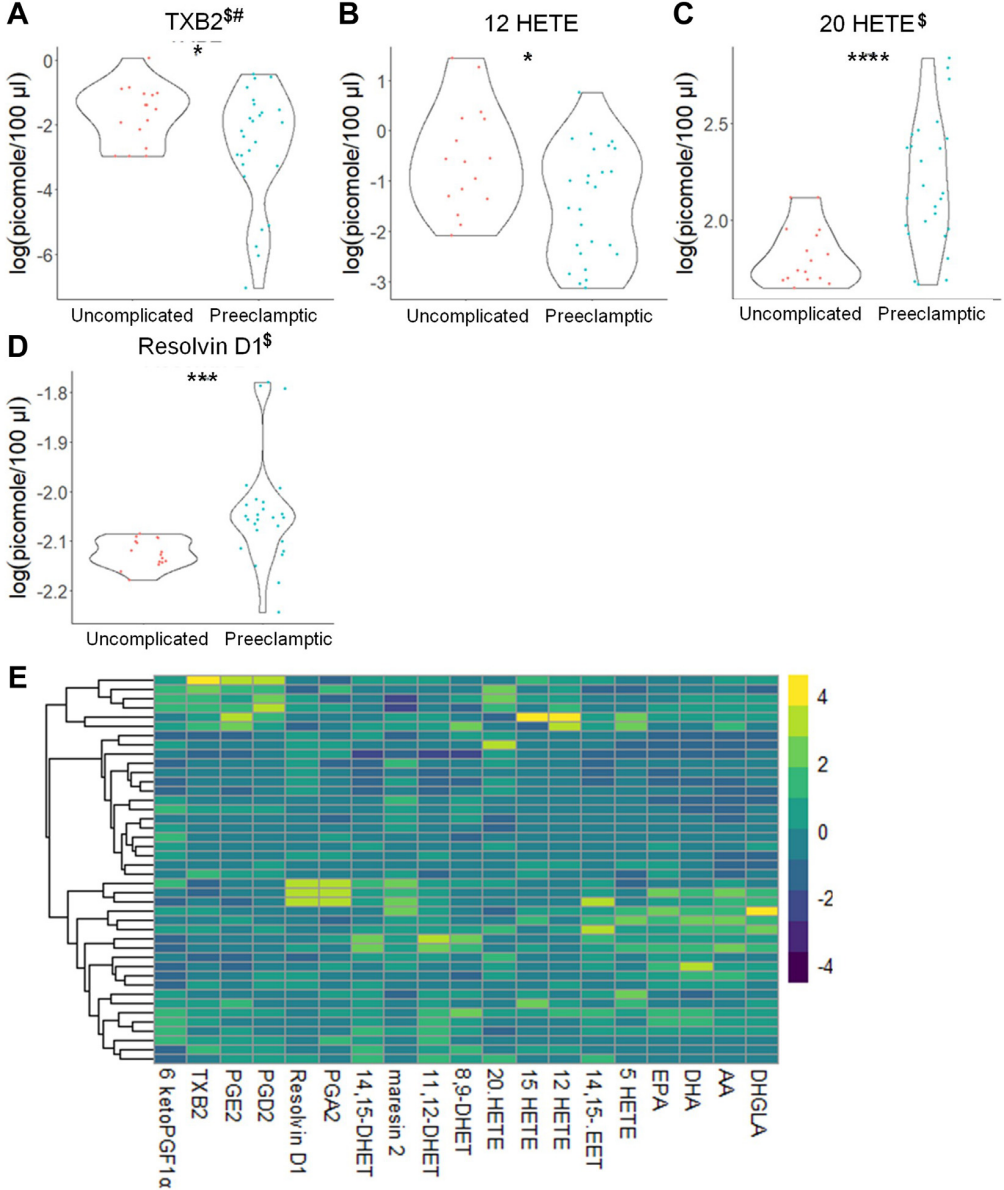
Distinct eicosanoids show significant differences in the plasma from uncomplicated term pregnant patients recruited from the general OB/GYN clinic versus total PE pregnant patients. A–D: Eicosanoid species that occurred at significantly different levels when comparing plasma from uncomplicated term pregnant patients recruited from the general OB/GYN clinic versus total PE pregnant patients using UPLC ESI-MS/MS as the detection method. Samples were analyzed by UPLC ESI-MS/MS within two weeks of acquisition. E: Heat map of all eicosanoid species that were detected via UPLC ESI-MS/MS in plasma (depicted as fold change). Samples were compared using unpaired students *t* test with Welch’s correction. Data shown are means ± SD depicted as violin plots, ∗*P* < 0.05, ∗∗∗*P* < 0.001, ∗∗∗∗*P* < 0.0001. The log-transformed data failing the Shapiro-Wilk Test are designated with a $. Nontransformed data were also analyzed by the Wilcoxon Sum Rank Test. Bioactive lipid mediators not found to be significantly different by the Wilcoxon Sum Rank Test are designated with a #. PE, preeclampsia; UPLC ultra-performance liquid chromatography.

**Fig. 8:**
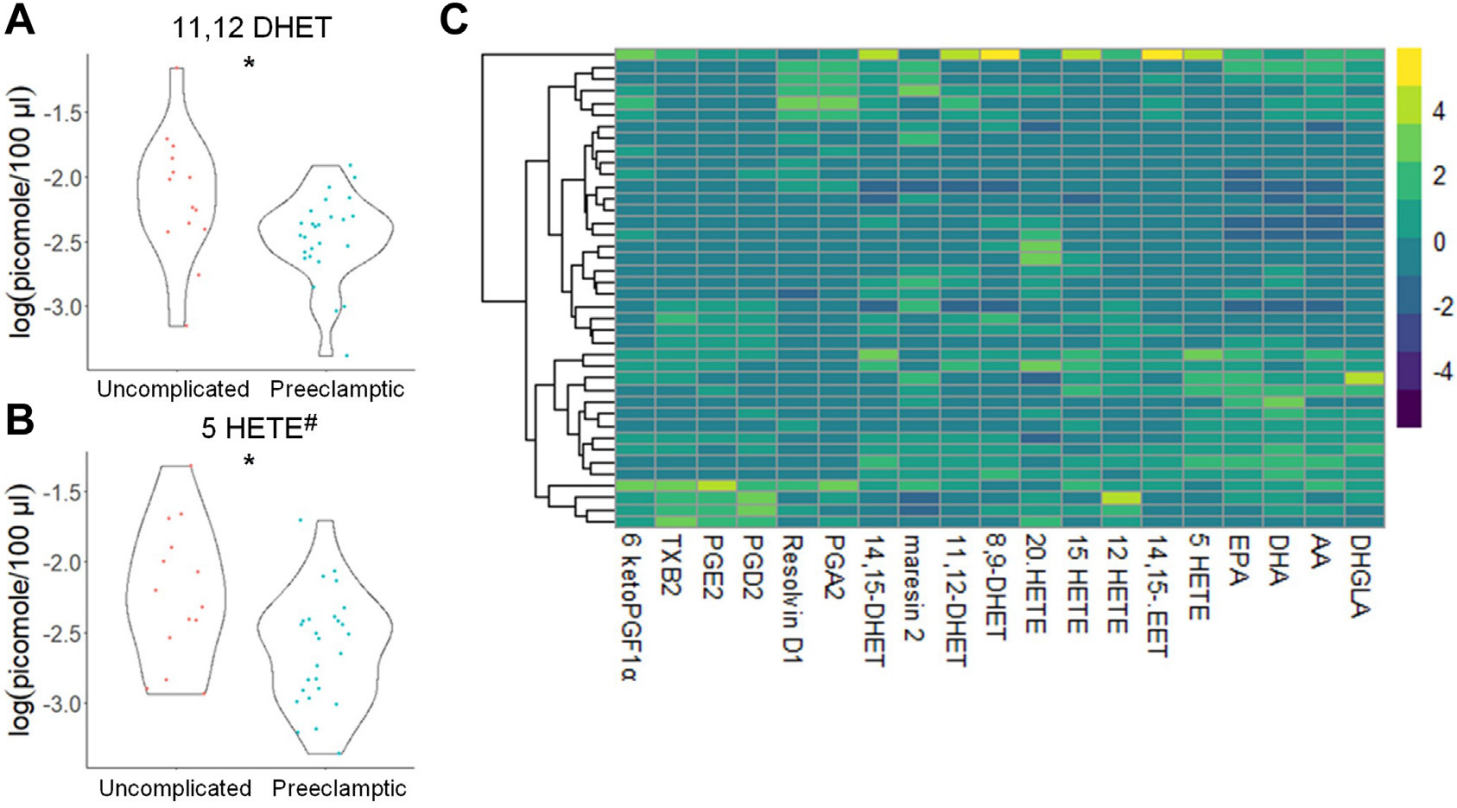
Distinct eicosanoids show significant differences in the plasma from uncomplicated term pregnant patients referred to high-risk OB/GYN clinic versus total PE pregnant patients. A and B: Eicosanoid species that occurred at significantly different levels when comparing plasma from uncomplicated term pregnant patients referred to high-risk OB/GYN clinic versus total PE pregnant patients using UPLC ESI-MS/MS as the detection method. Samples were analyzed by UPLC ESI-MS/MS within two weeks of acquisition. C: Heat map of all eicosanoid species that were detected via UPLC ESI-MS/MS in plasma (depicted as fold change). Samples were compared using unpaired students *t* test with Welch’s correction. Data shown are means ± SD depicted as violin plots, ∗*P* < 0.05. The log-transformed data failing the Shapiro-Wilk Test are designated with a $. Nontransformed data were also analyzed by the Wilcoxon Sum Rank Test. Bioactive lipid mediators not found to be significantly different by the Wilcoxon Sum Rank Test are designated with a #. PE, preeclampsia; UPLC ultra-performance liquid chromatography.

**Fig. 9:**
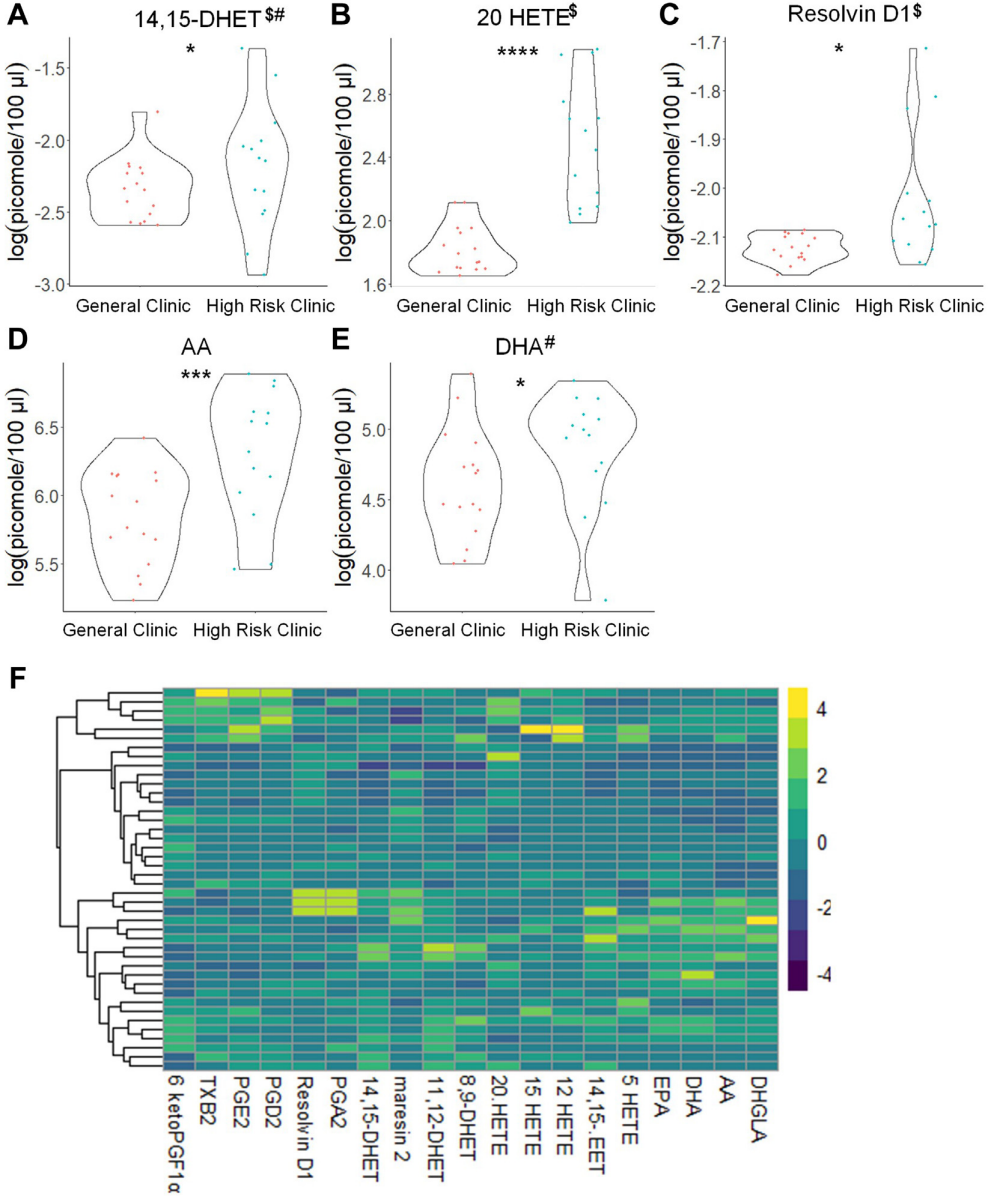
Distinct eicosanoids show significant differences in the plasma from uncomplicated term pregnant patients recruited from general OB/GYN clinic versus uncomplicated term pregnant patients referred to high-risk OB/GYN clinic. A–E: Eicosanoid species that occurred at significantly different levels when comparing plasma from normal term pregnant patients recruited from General OB/GYN clinic versus normal term pregnant patients referred to high-risk OB/GYN clinic using UPLC ESI-MS/MS as the detection method. Samples were analyzed by UPLC ESI-MS/MS within two weeks of acquisition. F: Heat map of all eicosanoid species that were detected via UPLC ESI-MS/MS in plasma (depicted as fold change). Samples were compared using unpaired students *t* test with Welch’s correction. Data shown are means ± SD depicted as violin plots, ∗*P* < 0.05, ∗∗∗*P* < 0.001, ∗∗∗∗*P* < 0.0001. The log-transformed data failing the Shapiro-Wilk Test are designated with a $. Nontransformed data were also analyzed by the Wilcoxon Sum Rank Test. Bioactive lipid mediators not found to be significantly different by the Wilcoxon Sum Rank Test are designated with a #. UPLC ultra-performance liquid chromatography.

Because approximately half of our study subjects were recruited from a high-risk clinic, we were able to examine whether the plasma levels of bioactive lipids can stratify patients at higher risk for PE development. In this regard, subjects with normal pregnancies from the general OB/GYN clinic (18 patients, aspirin was not prescribed) were initially compared to combined PE patients (26 patients). In contrast to the nonstratified findings, significant increases in 20-HETE and RvD1 and significant decreases in 12-HETE were observed (Fig. 7; supplemental Table S15). When comparing normal pregnancies from patients referred to high-risk OB/GYN clinic (14 patients, aspirin-prescribed) versus all patients with future PE diagnosis (26 patients), significant differences in eicosanoids were again observed in line with our nonstratified findings. Specifically, significant decreases in (±)11,12-DHET plasma levels were observed (Fig. 8; supplemental Table S16). Regardless of the general or high-risk clinic stratification, several SM, MonHex, and C1P species were observed to be decreased in the plasma of subjects who later developed PE, which was consistent with the combined, nonstratified study (supplemental Figs. S21–S28; supplemental Tables S17 and S18). Regardless, some sphingolipid changes were not observed when stratifying patients in this manner such as the decrease in de_18:1/14:0_ CER, de_18:1/26:1_ CER, and increase in de_18:1/18:1_ MonHex, which were only observed when comparing general OB/GYN clinic subjects with uncomplicated pregnancies to all subjects who later developed PE (supplemental Figs. S22, S24, S26 and S28; supplemental Tables S17 and S18). Because of these differences observed when stratifying patients based on clinic referral due to pregnancy risk factors, uncomplicated pregnancies from general OB/GYN clinic were compared to uncomplicated pregnancies from high-risk clinic. Significant increases in 20-HETE, RvD1, and AA were observed in patients referred to high-risk clinic with uncomplicated term pregnancies (Fig. 9; supplemental Table S19). A similar profile for sphingolipids was observed in patients referred to high-risk clinic as observed in patients who later developed PE such as increased levels of de_18:1/18:1_ MonHex between the two uncomplicated term, non-PE pregnancy groups (supplemental Figs. S29–S32; supplemental Table S20). These findings reveal that pregnancies with risk factors for complications already display indicators of modified plasma levels of specific bioactive lipids linked to later PE development. Furthermore, specific lipid-based biomarkers linked to PE development are many times independent of clinic/risk-factor stratification (e.g., de_18:1/26:1_ CER, de_18:1/18:1_ MonHex).

## Discussion

### Stratification of PE risk using plasma lipid profiles

The goal of our study was to identify lipid biomarkers for the early prediction of PE regardless of aspirin therapy as aspirin is only effective in 50% of patients at risk for PE. Most of our subjects were recruited from a high-risk clinic and were prescribed preventive aspirin therapy (84.6% of PE cases and 45.1% of normal pregnancies). Notably, we did not observe increases in TXB_2_ as expected before aspirin treatment. In contrast, we found no significant differences in plasma TXB_2_ levels as well as substantial variability. We attribute both the high variability and lack of increased TXB_2_ plasma levels associated with later PE development to the initiation of aspirin treatment, as well as variation in aspirin therapy compliance. This conclusion is supported by our findings that TXB_2_ levels were not associated with PE development when comparing uncomplicated term pregnancies from high-risk clinic (all prescribed aspirin) to total PE patients (84.6% of patients prescribed aspirin). One limitation to note, we did not have a measure of compliance in women prescribed aspirin, a reported efficacy factor in previous studies (62). Based on previous studies, compliance in our study could be as low as 50%. Regardless, this study provides the first insight into aspirin-independent lipid signatures for later PE development. For example, one additional AA-derived eicosanoid was decreased in subjects who ultimately developed PE, (±)11,12 DHET. There are few reports on relationships between (±)11,12 DHET and PE, but one study reported that DHETs were decreased in the urine of preeclamptic women (63), which is consistent with our findings in plasma. The DHET precursors, EETs, are strongly linked to cardiovascular and kidney disorders, as well as neutrophil function. Hydrolysis of EETs by soluble epoxide hydrolase is a mechanism to metabolize these lipid mediators to DHETs, and in relation to PE, methylation of the soluble epoxide hydrolase gene (*EPHX2*) promotor or polymorphisms in this gene are associated with PE development. Hence, the reduced levels of (±)11,12 DHET early in pregnancy may be indicative of an inability of the placenta to convert EETs to DHETs leading to elevated EETs in the placenta. These elevated EETs may inadvertently enhance neutrophil function and chemoattraction over time, which could promote neutrophil aggregation and a chronic sterile inflammatory response as observed in the spiral arteries of PE patients and in maternal subcutaneous and omental vessels in which neutrophils extensively infiltrate causing inflammation (64, 65). Interestingly, EETs, in general, are cardioprotective and anti-inflammatory (66), and thus, limiting conversion of EETs to DHETs may also be a response to maternal or placental inflammation in an attempt by the body to “stave off” PE development. Regardless, the link between *EPHX2* gene dysfunction and PE, as well as the lower DHET levels observed in this study and by others, suggest that DHET levels are a plausible pathophysiologic indicator of later PE development.

Significant differences in many sphingolipid species in the plasma of patients who develop PE were also observed in our study. These findings are both congruent and in contrast to recent studies where mass spectrometric analysis was utilized to create a plasma sphingolipid panel. One example is a study analyzing plasma from seven normal and seven PE patients. Although the study was not well powered statistically, the authors concluded that the first trimester maternal plasma sphingolipids (de_18:1/14:0_ CER (decreased), de_18:1/16:0_ SM (increased), and de_18:1/18:0_ SM (increased)) could serve as early biomarkers for PE development (43). Our findings differ with respect to SM, but concur with the results for de_18:1/14:0_ CER, a CER synthase 6 product (67), suggesting that this sphingolipid is a potential early biomarker for later development of PE. Indeed, CER synthase 6 has been linked to cellular stress responses and early embryonic development, which suggests that suppression of this enzyme is linked to dysregulated placental function (68). Consistent with our study is a recent report by Johnstone *et al.* demonstrating that S1P and So plasma levels were not different prior to PE (48). In contrast to our findings, Ilgisonis *et al.* demonstrated that de_18:1/20:0_ CER was increased in plasma as early as the first trimester in pregnant women who develop PE (69). In the same group of patients with PE whose serum S1P was lower than in healthy controls, higher de_18:1/16:0_, de_18:1/18:0_, de_18:1/20:0_, and de_18:1/24:0_ CER concentrations were found in both serum and placental tissue (70). We also previously showed significant increases in several sphingolipids: de_18:1/18:0_ CER, de_18:1/18:0_ SM, S1P, and sphinganine-1-phosphate in the placenta of women with PE (62). With the exceptions of de_18:1/26:1_ CER and de_18:1/18:1_ MonHexs, we observed decreases in most of the sphingolipid species regardless of the onset of PE or high-risk pregnancy factors. The increase in de_18:1/26:1_ CER was novel in comparison to previously reported studies. The increase in this CER species in the plasma was independent of aspirin therapy and stratification between PE with a preterm birth. de_18:1/26:1_ CER is synthesized by an understudied CER synthase, CER synthase 3. There are few studies dealing with this species of CER and CER synthase 3, and future research needs to determine the pathophysiological relevance of the elevated levels in PE development. The observed decrease in plasma SM levels early in pregnancy linked to later PE development is intriguing and possibly important regarding the pathophysiology of PE, as decreases in SM are observed in septic patients, usually linked to initial phases of an uncontrolled, hyperimmune/hyperinflammatory response induced by infection (71). PE has also been linked to a sterile hyperimmune response, and the reduced level of this SM may reflect an enhanced immune response linked to the development of placental dysfunction (72, 73). Of note, we cannot reconcile many of the differences between our study and other studies in regard to the plasma levels of sphingolipids and early gestational ages in pregnancies that develop PE. Whereas some similarities exist, such as the decrease in de_18:1/C14:0_ CER, most of our findings are opposite to these published studies. Some discrepancies may be explained by differences in biomatrices (e.g., serum vs. plasma) and gestational age. For other early gestational age studies of PE utilizing plasma as the biomatrix, only the prescription of aspirin was a major variable, although differences in diet, geographical location, and socioeconomic status cannot be ruled out as confounding variables. Furthermore, the levels of most sphingolipid species were significantly reduced in patients with normal pregnancies recruited from a high-risk clinic. Many of the reported studies on blood, plasma, and serum levels of sphingolipids in PE do not state the venue at which subjects were recruited (e.g., high-risk clinic vs. a routine clinic setting), which may also explain the differences in some of our findings compared to those of others.

### Stratification of PE patients

Additional analyses were undertaken for subjects stratified into patients later diagnosed with PE with a preterm or term birth. Patients later diagnosed with PE with a preterm showed reductions in 12-HETE and 15-HETE as well as increased levels of RvD1. Of note, these findings are in many cases in opposition to reports showing that levels of these eicosanoids are increased or not significantly modulated in PE patients (74), but as discussed above, differences in ancestry, gestational age, aspirin use, recruitment site, biomatrices analyzed, and sample processing may explain the disparities between these studies and ours. Indeed, in pregnancies complicated by PE, a significant increase in 15-HETE was shown in placental tissues and umbilical arteries when compared to a control group, but these data were acquired after birth had occurred (late gestational age). This observed increase in 15-HETE biosynthetic enzymes is logical as in late-term PE pregnancies, an increased constriction of human umbilical artery rings has been demonstrated to be dependent on 15-HETE levels. In turn, Yuan *et al.* reported that in PE complicated pregnancies, placental 15-HETE is produced in excess (75). Our study suggests a longitudinal dysregulation of specific eicosanoids like 15-HETE with levels being initially lower at early gestational time periods, but increasing in patients later diagnosed with PE with a preterm birth, possibly due to increased levels of biosynthetic enzymes in the placenta (76).

12-HETE, an eicosanoid linked to vasoconstriction, was previously reported to be increased in placenta and sera from PE patients in contrast to our findings (62, 76, 77). Additionally, HETEs, including 12-HETE, in women with PE with severe features were not significantly higher in ex vivo placental studies from our laboratories than for placentas from women with normal pregnancies, who had risk factors for PE and were prescribed aspirin (62). Our study is congruent with a study showing lower concentrations of 12-HETE in women with PE than the control group in relation to the placental trophoblast cells (36). The variability between these studies as to 12-HETE may be explained by the women receiving aspirin in our study, as these patients already had conditions associated with inflammation and oxidative stress that put them at risk of PE development. Eicosanoids also partition differently in specific biomatrices, and the processing of sera may induce 12-HETE biosynthesis as well as the biosynthesis of other eicosanoids during the clotting process. These findings may be important in regard to blood cell and platelet function but may not be indicative of eicosanoid levels in the circulation, possibly explaining the differences between studies. Furthermore, the gestational age was significantly different compared to our study, which may also explain why our findings for 12-HETE contrast with others (78). Future studies should take into account both the clinical parameters and the sampling and analysis parameters. Indeed, standardization of the type and timing of sample processing as well as storage time and the time to analysis would greatly facilitate comparisons across studies.

In this study, early increases in the anti-inflammatory lipid mediator, RvD1, were linked to later PE development, specifically in patients later diagnosed with PE with a preterm birth. This finding is in line with a recent longitudinal study by Perucci and coworkers that showed increased levels of RvD1 in early gestation (before 20 weeks). In contrast, pregnant women with PE had lower RvD1 levels at 30–34 weeks than those in normotensive pregnant women suggesting that RvD1 levels are modulated in a multiphasic fashion during gestation, becoming dysregulated as PE evolves. As with our findings with DHETs linked to possible increases in placental EETs, we surmise that the increased levels of RvD1 are an attempt by the body to reduce systemic inflammation associated with PE development, and increased levels of RvD1 were observed in the plasma levels from patients at risk for pregnancy complications regardless of PE development suggesting a plausible systemic inflammation in these patients. Furthermore, RvD1 levels have been noted to be higher in septic patients with lower survival, opposite to the hypothesized expectation of a decrease, since resolvins are linked to the resolution of a hyper-immune response (79). Therefore, RvD1 may be an early marker for an active sterile immune response in pregnant women who subsequently develop PE with an associated preterm birth (80).

Patients later diagnosed with PE with a preterm birth showed increased levels of de_18:1/26:1_ CER and de_18:1/18:1_ MonHex along with decreased levels of de_18:1/C14:0_ CER, but these differences were not observed in patients later diagnosed with PE with a term birth. Congruent with Johnstone *et al.*, our study did not find a correlation between S1P and So in regard to preterm PE and term PE (48). Overall, our study shows that multiple bioactive lipids are strong early predictors of later diagnoses of PE with an associated preterm birth with plasma increases of the sphingolipid, de_18:1/26:1_ CER, serving as a general biomarker of PE development in most cases, regardless of PE subtype or the risk of a patient developing pregnancy complications.

### Ancestry-specific variation in plasma lipid profiles in PE

Our study provided one of the first examinations of race-specific, lipid-based biomarkers for PE. Specifically, we found self-identified subjects of Western European descent (Caucasian) that developed PE showed significant decreases in the anti-inflammatory omega-3 polyunsaturated fatty acid, EPA, and DHA. Decreases in EPA and DHA levels have also been observed in the plasma from PE patients of Asian descent as well as associated with preterm births in Danish women (81, 82). The percent of Caucasian subjects in the Danish study was significantly higher than in our study, which may explain why these differences were only observed in our study when applying racial stratification. Indeed, other studies examining free fatty acids and eicosanoids in PE did not always present subject racial data, and specificity for a decrease in EPA plasma levels in PE patients of Western European and Asian descent could explain why a decrease in EPA levels was not observed in our race-combined studies and also not in self-designated subjects of Hispanics or African descent. Subjects of self-designated Hispanic origin showed significant and specific increases in PGA_2_. In contrast, we did not find specific differences in the plasma levels of subjects of self-designated African descent in comparison to the nonstratified analyses. This may simply be due to >50% of our patients being self-designated African descent and suggest that our findings are more indicative of early biomarkers for PE for patients predominantly of African ancestry. Indeed, many of the sphingolipid-based biomarkers of PE were not detected in patients of European and Hispanic descent, and racially disparate findings may explain some of the differences between the findings on specific bioactive lipids in this study linked to PE versus other reported studies. On the other hand, these differences between self-designated races may also be due to the lower statistical power in this study for the Western European and Hispanic patients.

From a causation standpoint, the decrease of anti-inflammatory EPA and DHA levels in Caucasian subjects developing PE is logical due to links between PE development and inflammation as well as high omega-3 fatty acid diets reported to reduce the incidence of severe PE (25). On the other hand, the increased levels of PGA_2_ observed for subjects of Hispanic ancestry represents a conundrum as PGA_2_ is a hypotensive agent. Hence, increased levels of PGA_2_ would be expected to repress PE development. As with RvD1, we surmise that early in gestation, these eicosanoids are modulated in an attempt by the body to prevent placental dysfunction. Although our study does support the conclusions that there are significant differences in the plasma levels of specific lipid mediators linked to PE development between self-determined ancestry, there are limitations in our study that should be noted. For example, we did not characterize our study subjects with ancestry-informative genetic markers. Additionally, our study did not take into account regional diet, urban versus rural environment, and other social determinants of health, all of which could affect levels of lipid-based biomarkers in the plasma.

### Novel observations

One novel finding from this study is that several species of C1P were significantly decreased in PE. C1P has not been proposed as a marker for PE or other human disease states. Our C1P findings potentially have pathophysiologic significance. C1P has been linked to inflammatory diseases through specific association and activation of group IVA cytosolic phospholipase A_2_ (cPLA_2_α), a key gene in the establishment and maintenance of pregnancy (83, 84, 85, 86, 87, 88). Thus, an early decrease in C1P in PE patients may be linked to the observed decreases in eicosanoids (57, 83, 84, 85, 86, 87, 88) in PE patients. Furthermore, C1P regulation of inflammation is complex as C1P can reduce the levels of inflammatory cytokines like TNFα by suppression of TNFα processing and maturation via inhibition of tumor necrosis factorα converting enzyme. Thus, the reduced levels of C1P may be indicative of increases in proinflammatory TNFα. Higher circulating levels of TNFα have been reported in PE patients (89).

Another novel finding is that 20-HETE levels are increased in patients recruited from a high-risk clinic. Additionally, when only normal pregnancies in subjects recruited from a general OB/GYN clinic are compared to patients who later develop PE, the plasma levels of 20-HETE were significantly increased. Both our combined, nonstratified findings and a cross-sectional study conducted by Jiang *et al.* (63) demonstrated no significant difference in plasma 20-HETE levels. However, our findings using this patient-risk stratification are consistent with a study showing that 20-HETE levels were higher in umbilical cord blood of PE patients (63). A study by Plenty *et al.* (74) also showed increased 20-HETE production in microsomes isolated from the placenta of PE women compared to the control group. Our study suggests that to observe increase in 20-HETE in the plasma requires stratifying patients into at-risk pregnancies. The observed increase in 20-HETE in at-risk pregnancies may have physiological and pathophysiological relevance. 20-HETE constricts blood vessels, including uterine arteries, and promotes the development of hypertension (90). Studies by Llinás *et al.* suggest involvement of 20-HETE in the induction of renal vasoconstriction, chronic uterine perfusion pressure abnormalities, and hypertension in pregnant rats (91). Additionally, administration of the 20-HETE inhibitor, HET0016, reduced vasoconstriction and improved uterine artery resistance in rat models (92). Thus, our study, when combined with these reports, implies that increased levels of 20-HETE arising from placental dysfunction may be linked to the development of PE.

### Summary

Although metabolic biomarkers for PE are not yet widely used in clinical practice, this study and the existing literature shows that metabolomics can potentially become a clinical tool for predicting and diagnosing PE, as well as clarifying the etiology and pathogenesis of the disease (69, 93). Our study shows that specific eicosanoids and sphingolipids can be accurately measured in the plasma of pregnant women and serve as early markers of the later development of PE with severe features regardless of patients being prescribed aspirin therapy (e.g., de_18:1/26:1_ CER). Due to the statistical power of this study and employment of multiple statistical analyses, new knowledge as to the stratification of patients into later PE diagnosis with preterm or term birth was also obtained, suggesting specific lipid signatures should be examined for these different subclasses of PE. Furthermore, our study serves as a foundation to reexamine past reports and for future studies to determine the effect of ancestry-linked differences in lipid-based biomarkers for precision-based analyses of later development of PE. Lastly, our study suggests that additional bioactive lipids may be therapeutic targets for prevention of pregnancy-related complications (e.g., 20-HETE).

## Data availability

All data are contained within the article.

## Supplemental data

This article contains supplemental data.

## Conflict of interest

All authors of this article declare that they have no competing financial interests.
