# Supplementary material for: Bioactive lipid mediators in plasma are predictors of preeclampsia irrespective of aspirin therapy
Source: J Lipid Res. 2023 Apr 27;64(6):100377. doi: 10.1016/j.jlr.2023.100377 (PMC10230265; doi:10.1016/j.jlr.2023.100377)
Supplement: Supplemental Tables S1–S20 and Figures S1–S32 [file mmc1.docx]

**SUPPLEMENTAL DATA**

**Bioactive lipid mediators in plasma are predictors of preeclampsia irrespective of aspirin therapy**

Daniel J. Stephenson at el

| **Analyte ID** | **Q1 Mass (Da)** | **Q2 Mass (Da)** | **DP (volt)** | **EP (volt)** | **CE (volt)** | **CXP (volt)** |
| --- | --- | --- | --- | --- | --- | --- |
| **6keto PGF1α-d4** | **373.2** | **167.0** | **-80** | **-14** | **-33** | **-15** |
| **6-keto PGF1α** | **369.2** | **163.0** | **-80** | **-14** | **-33** | **-15** |
| **8-iso PGF2α-d4** | **357.4** | **197.1** | **-150** | **-13** | **-33** | **-14** |
| **8-iso PGF2α** | **353.4** | **193.0** | **-130** | **-13** | **-31** | **-11** |
| **TXB2-d4** | **373.2** | **173.0** | **-80** | **-13** | **-23** | **-15** |
| **TXB2** | **369.2** | **169.0** | **-80** | **-13** | **-25** | **-15** |
| **5-iPF2α-VI-d11** | **364.2** | **115.0** | **-90** | **-12** | **-28** | **-20** |
| **5-iPF2α-VI** | **353.2** | **114.9** | **-90** | **-12** | **-28** | **-16** |
| **PGE2-d9** | **360.2** | **280.3** | **-80** | **-13** | **-24** | **-12** |
| **PGE2** | **351.2** | **271.2** | **-80** | **-13** | **-25** | **-12** |
| **PGF2α-d9** | **362.2** | **193.0** | **-70** | **-10** | **-35** | **-18** |
| **PGF2α** | **353.2** | **193.0** | **-70** | **-10** | **-31** | **-18** |
| **PGD2-d9** | **360.2** | **280.3** | **-80** | **-10** | **-24** | **-12** |
| **PGD2** | **351.2** | **271.2** | **-80** | **-10** | **-23** | **-12** |
| **RvD3-d5** | **380.2** | **147.0** | **-68** | **-10** | **-24** | **-11** |
| **RvD3** | **375.2** | **147.0** | **-68** | **-10** | **-24** | **-11** |
| **RvD2-d5** | **380.2** | **141.0** | **-80** | **-12** | **-21** | **-10** |
| **RvD2** | **375.2** | **141.0** | **-80** | **-12** | **-22** | **-10** |
| **PGE1-d4** | **357.2** | **239.0** | **-105** | **-13** | **-20** | **-19** |
| **PGE1** | **353.2** | **235.0** | **-105** | **-13** | **-19** | **-19** |
| **RvD1-d5** | **380.2** | **141.0** | **-70** | **-13** | **-20** | **-10** |
| **RvD1** | **375.2** | **141.0** | **-70** | **-13** | **-22** | **-10** |
| **Lipoxin A4-d5** | **356.3** | **114.9** | **-90** | **-14** | **-21** | **-20** |
| **Lipoxin A4** | **351.3** | **114.8** | **-90** | **-13** | **-20** | **-15** |
| **PGA2-d4** | **337.2** | **275.3** | **-80** | **-14** | **-19** | **-12** |
| **PGA2** | **333.2** | **271.2** | **-80** | **-14** | **-19** | **-12** |
| **LTD4-d5** | **500.3** | **177.0** | **-105** | **-10** | **-24** | **-16** |
| **LTD4** | **495.3** | **176.9** | **-105** | **-10** | **-19** | **-14** |
| **LTC4-d5** | **629.3** | **272.1** | **-50** | **-13** | **-30** | **-13** |
| **LTC4** | **624.3** | **272.1** | **-60** | **-13** | **-30** | **-13** |
| **LTE4-d5** | **443.2** | **338.3** | **-80** | **-10** | **-24** | **-15** |
| **LTE4** | **438.2** | **333.1** | **-80** | **-10** | **-23** | **-15** |
| **LTB4-d4** | **339.2** | **197.0** | **-95** | **-14** | **-21** | **-16** |
| **LTB4** | **335.2** | **195.0** | **-95** | **-14** | **-22** | **-16** |
| **Maresin 2-d5** | **364.2** | **221.1** | **-65** | **-14** | **-16** | **-11** |
| **Maresin 2** | **359.2** | **221.1** | **-65** | **-14** | **-16** | **-11** |
| **(±)14,15-DHET-d11** | **348.2** | **207.0** | **-110** | **-14** | **-24** | **-14** |
| **(±)14,15-DHET** | **337.2** | **207.0** | **-110** | **-14** | **-26** | **-14** |
| **15-deoxy-Δ12,14-PGJ2-d4** | **319.2** | **275.3** | **-100** | **-9** | **-22** | **-10** |
| **15-deoxy-Δ12,14-PGJ2** | **315.2** | **271.2** | **-100** | **-9** | **-21** | **-10** |
| **(±)11,12-DHET-d11** | **348.2** | **167.0** | **-85** | **-12** | **-25** | **-14** |
| **(±)11,12-DHET** | **337.2** | **167.0** | **-85** | **-12** | **-24** | **-14** |
| **(±)8,9-DHET-d11** | **348.2** | **185.2** | **-93** | **-9** | **-23** | **-13** |
| **(±)8,9-DHET** | **337.2** | **185.2** | **-95** | **-10** | **-20** | **-13** |
| **20-HETE-d6** | **325.2** | **281.3** | **-85** | **-13** | **-21** | **-12** |
| **20-HETE** | **319.2** | **275.2** | **-85** | **-13** | **-21** | **-12** |
| **15 HETE-d8** | **327.2** | **226.0** | **-116** | **-13** | **-16** | **-16** |
| **15 HETE** | **319.2** | **219.0** | **-116** | **-13** | **-19** | **-16** |
| **12 HETE-d8** | **327.2** | **184.1** | **-90** | **-13** | **-20** | **-16** |
| **12 HETE** | **319.2** | **178.9** | **-90** | **-13** | **-19** | **-16** |
| **(±)14(15)-EET-d11** | **330.2** | **219.1** | **-90** | **-13** | **-15** | **-15** |
| **(±)14(15)-EET** | **319.2** | **219.1** | **-90** | **-13** | **-15** | **-15** |
| **5 HETE d8** | **327.2** | **116.0** | **-90** | **-13** | **-18** | **-10** |
| **5 HETE** | **319.2** | **115.0** | **-90** | **-13** | **-20** | **-10** |
| **(±)8(9)-EET-d11** | **330.2** | **123.0** | **-90** | **-12** | **-18** | **-11** |
| **(±)8(9)-EET** | **319.2** | **123.0** | **-90** | **-12** | **-18** | **-11** |
| **EPA d5** | **306.2** | **262.3** | **-86** | **-10** | **-15** | **-18** |
| **EPA** | **301.2** | **257.1** | **-86** | **-10** | **-17** | **-18** |
| **DHA-d5** | **332.2** | **288.3** | **-95** | **-12** | **-14** | **-12** |
| **DHA** | **327.2** | **283.2** | **-95** | **-12** | **-19** | **-12** |
| **AA-d8** | **311.2** | **267.3** | **-150** | **-13** | **-18** | **-16** |
| **AA** | **303.2** | **259.2** | **-150** | **-13** | **-17** | **-14** |
| **DHGLA-d6** | **311.2** | **267.2** | **-105** | **-14** | **-20** | **-13** |
| **DHGLA** | **305.2** | **261.2** | **-90** | **-10** | **-43** | **-16** |

**Supplemental Table 1. Mass spectrometric parameters for eicosanoids analyzed by UPLC ESI-MS/MS.** The table depicts the eicosanoids and deuterated standards analyzed in the UPLC ESI-MS/MS protocol. The mass spectrometric parameters utilized for each noted lipid species are provided.

| **Analyte ID** | **Q1 Mass (Da)** | **Q2 Mass (Da)** | **DP (volt)** | **EP (volt)** | **CE (volt)** | **CXP (volt)** |
| --- | --- | --- | --- | --- | --- | --- |
| **d17:1 So** | **286.4** | **268.3** | **120** | **10** | **15** | **10** |
| **d17:0 Sa** | **288.4** | **270.4** | **120** | **10** | **21** | **10** |
| **d18:1 So** | **300.5** | **282.3** | **120** | **10** | **21** | **10** |
| **d18:0 Sa** | **302.5** | **284.3** | **120** | **10** | **21** | **10** |
| **d17:1 So1P** | **366.4** | **250.4** | **120** | **10** | **23** | **10** |
| **d17:0 Sa1P** | **368.4** | **252.4** | **120** | **10** | **23** | **10** |
| **d18:1 So1P** | **380.4** | **264.4** | **120** | **10** | **25** | **10** |
| **d18:0 Sa1P** | **382.4** | **266.4** | **120** | **10** | **25** | **10** |
| **Cer(de18:1/12:0)** | **482.6** | **264.4** | **80** | **10** | **41** | **10** |
| **Cer(de18:1/14:0)** | **510.7** | **264.4** | **80** | **10** | **43.5** | **10** |
| **Cer(de18:1/16:0)** | **538.7** | **264.4** | **80** | **10** | **46** | **10** |
| **Cer(de18:1/18:1)** | **564.7** | **264.4** | **80** | **10** | **48.5** | **10** |
| **Cer(de18:1/18:0)** | **566.7** | **264.4** | **80** | **10** | **48.5** | **10** |
| **Cer(de18:1/20:0)** | **594.7** | **264.4** | **80** | **10** | **51** | **10** |
| **Cer(de18:1/22:0)** | **622.8** | **264.4** | **80** | **10** | **53.5** | **10** |
| **Cer(de18:1/24:1)** | **648.9** | **264.4** | **80** | **10** | **56** | **10** |
| **Cer(de18:1/24:0)** | **650.9** | **264.4** | **80** | **10** | **56** | **10** |
| **Cer(de18:1/26:1)** | **676.9** | **264.4** | **80** | **10** | **58.5** | **10** |
| **Cer(de18:1/26:0)** | **678.9** | **264.4** | **80** | **10** | **58.5** | **10** |
| **C1P(de18:1/12:0)** | **562.4** | **264.4** | **80** | **10** | **41** | **10** |
| **C1P(de18:1/14:0)** | **590.4** | **264.4** | **80** | **10** | **43.5** | **10** |
| **C1P(de18:1/16:0)** | **618.5** | **264.4** | **80** | **10** | **46** | **10** |
| **C1P(de18:1/18:1)** | **644.5** | **264.4** | **80** | **10** | **48.5** | **10** |
| **C1P(de18:1/18:0)** | **646.5** | **264.4** | **80** | **10** | **48.5** | **10** |
| **C1P(de18:1/20:0)** | **674.4** | **264.4** | **80** | **10** | **51** | **10** |
| **C1P(de18:1/22:0)** | **702.7** | **264.4** | **80** | **10** | **53.5** | **10** |
| **C1P(de18:1/24:1)** | **728.6** | **264.4** | **80** | **10** | **56** | **10** |
| **C1P(de18:1/24:0)** | **730.6** | **264.4** | **80** | **10** | **56** | **10** |
| **C1P(de18:1/26:1)** | **756.7** | **264.4** | **80** | **10** | **58.5** | **10** |
| **C1P(de18:1/26:0)** | **758.7** | **264.4** | **80** | **10** | **58.5** | **10** |
| **MonHex(de18:1/12:0)** | **644.6** | **264.4** | **80** | **10** | **41** | **10** |
| **MonHex(de18:1/14:0)** | **672.6** | **264.4** | **80** | **10** | **43.5** | **10** |
| **MonHex(de18:1/16:0)** | **700.7** | **264.4** | **80** | **10** | **46** | **10** |
| **MonHex(de18:1/18:1)** | **726.7** | **264.4** | **80** | **10** | **48.5** | **10** |
| **MonHex(de18:1/18:0)** | **728.7** | **264.4** | **80** | **10** | **48.5** | **10** |
| **MonHex(de18:1/20:0)** | **756.7** | **264.4** | **80** | **10** | **51** | **10** |
| **MonHex(de18:1/22:0)** | **784.8** | **264.4** | **80** | **10** | **53.5** | **10** |
| **MonHex(de18:1/24:1)** | **810.9** | **264.4** | **80** | **10** | **56** | **10** |
| **MonHex(de18:1/24:0)** | **812.9** | **264.4** | **80** | **10** | **56** | **10** |
| **MonHex(de18:1/26:1)** | **838.9** | **264.4** | **80** | **10** | **58.5** | **10** |
| **MonHex(de18:1/26:0)** | **840.9** | **264.4** | **80** | **10** | **58.5** | **10** |
| **SM(de18:1/12:0)** | **647.7** | **184.4** | **80** | **10** | **41** | **10** |
| **SM(de18:1/14:0)** | **675.7** | **184.4** | **80** | **10** | **43.5** | **10** |
| **SM(de18:1/16:0)** | **703.8** | **184.4** | **80** | **10** | **46** | **10** |
| **SM(de18:1/18:1)** | **729.8** | **184.4** | **80** | **10** | **48.5** | **10** |
| **SM(de18:1/18:0)** | **731.8** | **184.4** | **80** | **10** | **48.5** | **10** |
| **SM(de18:1/20:0)** | **759.9** | **184.4** | **80** | **10** | **51** | **10** |
| **SM(de18:1/22:0)** | **787.9** | **184.4** | **80** | **10** | **53.5** | **10** |
| **SM(de18:1/24:1)** | **813.9** | **184.4** | **80** | **10** | **56** | **10** |
| **SM(de18:1/24:0)** | **815.9** | **184.4** | **80** | **10** | **56** | **10** |
| **SM(de18:1/26:1)** | **841.9** | **184.4** | **80** | **10** | **58.5** | **10** |
| **SM(de18:1/26:0)** | **843.9** | **184.4** | **80** | **10** | **58.5** | **10** |

**Supplemental Table 2. Mass spectrometric parameters for sphingolipids analyzed by UPLC ESI-MS/MS.** The table depicts the sphingolipid species analyzed in the UPLC ESI-MS/MS protocol. The mass spectrometric parameters utilized for each noted sphingolipid species are provided.

| **Analyte** |  | **Normal (n=31)** | | | |  |  | **PE (n=26)** | | |
| --- | --- | --- | --- | --- | --- | --- | --- | --- | --- | --- |
|  |  | **pmol/100 µL Plasma** | | | |  |  | **pmol/100 µL Plasma** | | |
| **6-keto PGF1α** |  | 0.008 | ± | 0.004 |  | |  | 0.007 | ± | 0.002 |
| **TXB_2_** |  | 0.231 | ± | 0.219 |  | |  | 0.169 | ± | 0.186 |
| **PGE_2_** |  | 0.027 | ± | 0.025 |  | |  | 0.018 | ± | 0.013 |
| **PGD_2_** |  | 0.03 | ± | 0.025 |  | |  | 0.037 | ± | 0.034 |
| **Resolvin D1** |  | 0.126 | ± | 0.015 |  | |  | 0.131 | ± | 0.015* |
| **PGA_2_** |  | 0.232 | ± | 0.024 |  | |  | 0.232 | ± | 0.02 |
| **(±)14,15 DHET** |  | 0.109 | ± | 0.042 |  | |  | 0.092 | ± | 0.029 |
| **Maresin 2** |  | 0.009 | ± | 0.004 |  | |  | 0.01 | ± | 0.005 |
| **(±)11,12 DHET** |  | 0.115 | ± | 0.05 |  | |  | 0.088 | ± | 0.026* |
| **(±) 8,9 DHET** |  | 0.03 | ± | 0.014 |  | |  | 0.025 | ± | 0.007 |
| **20-HETE** |  | 9.396 | ± | 4.891 |  | |  | 9.394 | ± | 3.168 |
| **15-HETE** |  | 0.121 | ± | 0.084 |  | |  | 0.079 | ± | 0.032* |
| **12-HETE** |  | 0.682 | ± | 0.932 |  | |  | 0.405 | ± | 0.447 |
| **(±)14,15 EET** |  | 0.033 | ± | 0.045 |  | |  | 0.023 | ± | 0.012 |
| **5-HETE** |  | 0.174 | ± | 0.123 |  | |  | 0.131 | ± | 0.078 |
| **EPA** |  | 15.347 | ± | 6.879 |  | |  | 15.419 | ± | 9.066 |
| **DHA** |  | 121.26 | ± | 46.19 |  | |  | 134.094 | ± | 61.682 |
| **AA** |  | 473.16 | ± | 217.92 |  | |  | 469.597 | ± | 223.417 |
| **DHGLA** |  | 80.793 | ± | 36.344 |  | |  | 84.143 | ± | 59.805 |

**Supplemental Table 3. Eicosanoid profile observed in subjects with uncomplicated term and PE pregnancies.**

Raw, non-transformed eicosanoid levels observed in patients with uncomplicated pregnancies vs. patients later diagnosed with PE. Samples were taken prior to 24 weeks gestation and analyzed by UPLC ESI-M/S within two weeks of acquisition. Raw data were statistically compared using the Wilcoxon Sum Rank Test. The data presented are in Means ± SD in pmol lipid/100 µL plasma. Significance is represented as *p <0.05; **p<0.01; ***p<0.001; ****p<0.0001.

| **Analyte** | |  | | **Normal (n=31) pmol/50µL Plasma)** | | |  | | **PE (n=26)**  **pmol/50µL Plasma** | | |  |
| --- | --- | --- | --- | --- | --- | --- | --- | --- | --- | --- | --- | --- |
| **de18:1/14:0 Cer** |  | | 0.711 | | ± | 0.223 |  | 0.530 | | ± | 0.216** | |
| **de18:1/16:0 Cer** |  | | 2.569 | | ± | 1.080 |  | 1.622 | | ± | 0.831** | |
| **de18:1/18:0 Cer** |  | | 3.305 | | ± | 1.891 |  | 2.203 | | ± | 1.419* | |
| **de18:1/20:0 Cer** |  | | 9.335 | | ± | 3.983 |  | 7.007 | | ± | 3.666* | |
| **de18:1/22:0 Cer** |  | | 63.652 | | ± | 29.611 |  | 47.675 | | ± | 22.264 | |
| **de18:1/24:1 Cer** |  | | 47.217 | | ± | 16.042 |  | 42.753 | | ± | 19.247 | |
| **de18:1/24:0 Cer** |  | | 168.034 | | ± | 77.278 |  | 121.972 | | ± | 52.833* | |
| **de18:1/26:1 Cer** |  | | 1.839 | | ± | 0.536 |  | 2.530 | | ± | 0.789*** | |
| **de18:1/26:0 Cer** |  | | 2.275 | | ± | 1.063 |  | 1.624 | | ± | 0.987**** | |
| **de18:1/14:0 C1P** |  | | 0.921 | | ± | 0.391 |  | 0.652 | | ± | 0.269^$^ | |
| **de18:1/16:0 C1P** |  | | 55.621 | | ± | 20.155 |  | 34.717 | | ± | 18.148*** | |
| **de18:1/22:0 C1P** |  | | 0.951 | | ± | 0.726 |  | 1.107 | | ± | 0.467**** | |
| **de18:1/24:1 C1P** |  | | 1.876 | | ± | 0.868 |  | 1.115 | | ± | 0.929**** | |
| **de18:1/24:0 C1P** |  | | 4.421 | | ± | 1.942 |  | 2.681 | | ± | 1.850**** | |
| **de18:1/14:0 SM** |  | | 97.654 | | ± | 36.269 |  | 61.833 | | ± | 34.830 | |
| **de18:1/16:0 SM** |  | | 83.263 | | ± | 33.211 |  | 49.406 | | ± | 32.552**** | |
| **de18:1/18:1 SM** |  | | 165.409 | | ± | 64.728 |  | 100.689 | | ± | 63.499* | |
| **de18:1/18:0 SM** |  | | 1.466 | | ± | 0.318 |  | 1.302 | | ± | 0.466**** | |
| **de18:1/20:0 SM** |  | | 1.485 | | ± | 0.691 |  | 0.957 | | ± | 0.786* | |
| **de18:1/22:0 SM** |  | | 287.493 | | ± | 35.351 |  | 275.209 | | ± | 32.059 | |
| **de18:1/24:1 SM** |  | | 262.125 | | ± | 81.104 |  | 215.547 | | ± | 72.705* | |
| **de18:1/24:0 SM** |  | | 205.154 | | ± | 36.712 |  | 160.882 | | ± | 49.253* | |
| **de18:1/26:1 SM** |  | | 302.781 | | ± | 47.680 |  | 280.039 | | ± | 51.468* | |
| **de18:1/26:0 SM** |  | | 390.526 | | ± | 39.575 |  | 360.336 | | ± | 44.657**** | |
| **de18:1/14:0 MonHex** |  | | 539.834 | | ± | 91.772 |  | 531.642 | | ± | 74.826** | |
| **de18:1/16:0 MonHex** |  | | 546.530 | | ± | 115.308 |  | 500.887 | | ± | 110.969**** | |
| **de18:1/18:1 MonHex** |  | | 584.941 | | ± | 144.885 |  | 503.640 | | ± | 107.065**** | |
| **de18:1/18:0 MonHex** |  | | 30.257 | | ± | 9.112 |  | 23.247 | | ± | 7.976* | |
| **de18:1/20:0 MonHex** |  | | 9.851 | | ± | 4.287 |  | 7.401 | | ± | 3.048**** | |
| **de18:1/22:0 MonHex** |  | | 0.233 | | ± | 0.148 |  | 0.176 | | ± | 0.052**** | |
| **de18:1/24:1 MonHex** |  | | 4.691 | | ± | 1.869 |  | 3.312 | | ± | 1.336**** | |
| **de18:1/24:0 MonHex** |  | | 1.748 | | ± | 0.705 |  | 1.086 | | ± | 0.521**** | |
| **de18:1/26:1 MonHex** |  | | 3.798 | | ± | 1.502 |  | 1.780 | | ± | 1.282** | |
| **de18:1/26:0 MonHex** |  | | 2.432 | | ± | 1.014 |  | 1.540 | | ± | 0.734* | |
| **So** |  | | 1.459 | | ± | 1.302 |  | 1.229 | | ± | 0.746 | |
| **S1P** |  | | 44.116 | | ± | 17.025 |  | 41.853 | | ± | 12.541 | |
| **Sa1P** |  | | 7.212 | | ± | 3.123 |  | 6.780 | | ± | 2.274 | |

**Supplemental Table 4. Sphingolipid profile observed in subjects with uncomplicated term and PE pregnancies.**

Raw, non-transformed sphingolipid levels observed in patients with uncomplicated pregnancies vs. patients later diagnosed with PE. Samples were taken prior to 24 weeks gestation and analyzed by UPLC ESI-M/S within two weeks of acquisition. Raw data were statistically compared using the Wilcoxon Sum Rank Test. The data presented are in Means ± SD in pmol lipid/50 µL plasma. Significance is represented as *p <0.05; **p<0.01; ***p<0.001; ****p<0.0001.

| **Analyte** |  | **Uncomplicated (n=31)** | | | |  |  | **Preterm Preeclamptic (n=12)** | | |
| --- | --- | --- | --- | --- | --- | --- | --- | --- | --- | --- |
|  |  | **pmol/100 µL Plasma** | | | |  |  | **pmol/100 µL Plasma** | | |
| **6-keto PGF1α** |  | 0.008 | ± | 0.004 |  | |  | 0.007 | ± | 0.002 |
| **TXB_2_** |  | 0.231 | ± | 0.219 |  | |  | 0.219 | ± | 0.221 |
| **PGE_2_** |  | 0.027 | ± | 0.025 |  | |  | 0.022 | ± | 0.014* |
| **PGD_2_** |  | 0.030 | ± | 0.025 |  | |  | 0.040 | ± | 0.038 |
| **Resolvin D1** |  | 0.126 | ± | 0.015 |  | |  | 0.124 | ± | 0.008**** |
| **PGA_2_** |  | 0.232 | ± | 0.024 |  | |  | 0.224 | ± | 0.009 |
| **(±)14,15 DHET** |  | 0.109 | ± | 0.042 |  | |  | 0.097 | ± | 0.028 |
| **Maresin 2** |  | 0.009 | ± | 0.004 |  | |  | 0.010 | ± | 0.004 |
| **(±)11,12 DHET** |  | 0.115 | ± | 0.050 |  | |  | 0.092 | ± | 0.022 |
| **(±) 8,9 DHET** |  | 0.030 | ± | 0.014 |  | |  | 0.028 | ± | 0.007 |
| **20-HETE** |  | 9.396 | ± | 4.891 |  | |  | 9.853 | ± | 3.864 |
| **15-HETE** |  | 0.121 | ± | 0.084 |  | |  | 0.093 | ± | 0.034** |
| **12-HETE** |  | 0.682 | ± | 0.932 |  | |  | 0.568 | ± | 0.505** |
| **(±)14,15 EET** |  | 0.033 | ± | 0.045 |  | |  | 0.023 | ± | 0.011 |
| **5-HETE** |  | 0.174 | ± | 0.123 |  | |  | 0.151 | ± | 0.089 |
| **EPA** |  | 15.347 | ± | 6.879 |  | |  | 15.928 | ± | 9.262 |
| **DHA** |  | 121.261 | ± | 46.190 |  | |  | 134.894 | ± | 52.404 |
| **AA** |  | 473.159 | ± | 217.916 |  | |  | 492.520 | ± | 227.940 |
| **DHGLA** |  | 80.793 | ± | 36.344 |  | |  | 95.381 | ± | 69.951 |

**Supplemental Table 5. Eicosanoid profile observed in uncomplicated term pregnant patients vs. PE pregnancies with a preterm birth.** Raw, non-transformed eicosanoid levels observed in patients with uncomplicated pregnancies vs. patients later diagnosed with PE and a preterm birth. Samples were taken prior to 24 weeks gestation and analyzed by UPLC ESI-M/S within two weeks of acquisition. Raw data were statistically compared using the Wilcoxon Sum Rank Test. The data presented are in Means ± SD in pmol lipid/100 µL plasma. Significance is represented as *p <0.05; **p<0.01; ***p<0.001; ****p<0.0001.

| **Analyte** |  | **Uncomplicated (n=31)** | | | |  |  | **Term Preeclamptic (n=14)** | | |
| --- | --- | --- | --- | --- | --- | --- | --- | --- | --- | --- |
|  |  | **pmol/100 µL Plasma** | | | |  |  | **pmol/100 µL Plasma** | | |
| **6-keto PGF1α** |  | 0.008 | ± | 0.004 |  | |  | 0.007 | ± | 0.003 |
| **TXB_2_** |  | 0.231 | ± | 0.219 |  | |  | 0.232 | ± | 0.223 |
| **PGE_2_** |  | 0.027 | ± | 0.025 |  | |  | 0.023 | ± | 0.014 |
| **PGD_2_** |  | 0.030 | ± | 0.025 |  | |  | 0.043 | ± | 0.039 |
| **Resolvin D1** |  | 0.126 | ± | 0.015 |  | |  | 0.125 | ± | 0.009* |
| **PGA_2_** |  | 0.232 | ± | 0.024 |  | |  | 0.221 | ± | 0.010 |
| **(±)14,15 DHET** |  | 0.109 | ± | 0.042 |  | |  | 0.099 | ± | 0.028 |
| **Maresin 2** |  | 0.009 | ± | 0.004 |  | |  | 0.010 | ± | 0.005 |
| **(±)11,12 DHET** |  | 0.115 | ± | 0.050 |  | |  | 0.090 | ± | 0.023* |
| **(±) 8,9 DHET** |  | 0.030 | ± | 0.014 |  | |  | 0.029 | ± | 0.007 |
| **20-HETE** |  | 9.396 | ± | 4.891 |  | |  | 10.172 | ± | 3.803 |
| **15-HETE** |  | 0.121 | ± | 0.084 |  | |  | 0.096 | ± | 0.033* |
| **12-HETE** |  | 0.682 | ± | 0.932 |  | |  | 0.603 | ± | 0.506 |
| **(±)14,15 EET** |  | 0.033 | ± | 0.045 |  | |  | 0.024 | ± | 0.011 |
| **5-HETE** |  | 0.174 | ± | 0.123 |  | |  | 0.152 | ± | 0.091 |
| **EPA** |  | 15.347 | ± | 6.879 |  | |  | 16.479 | ± | 9.346 |
| **DHA** |  | 121.261 | ± | 46.190 |  | |  | 137.234 | ± | 53.480 |
| **AA** |  | 473.159 | ± | 217.916 |  | |  | 511.077 | ± | 224.728 |
| **DHGLA** |  | 80.793 | ± | 36.344 |  | |  | 97.630 | ± | 71.880 |

**Supplemental Table 6. Eicosanoid profile observed in uncomplicated term pregnant patients vs. PE pregnancies with a term birth.** Raw, non-transformed eicosanoid levels observed in patients with uncomplicated pregnancies vs. patients later diagnosed with PE and a term birth. Samples were taken prior to 24 weeks gestation and analyzed by UPLC ESI-M/S within two weeks of acquisition. Raw data were statistically compared using the Wilcoxon Sum Rank Test. The data presented are in Means ± SD in pmol lipid/100 µL plasma. Significance is represented as *p <0.05; **p<0.01; ***p<0.001; ****p<0.0001.

| **Analyte** | |  | | **Uncomplicated (n=31) pmol/50µL Plasma** | | |  | | **Preterm Preeclamptic (n=12) pmol/50µL Plasma** | | |  |
| --- | --- | --- | --- | --- | --- | --- | --- | --- | --- | --- | --- | --- |
| **de18:1/14:0 Cer** |  | | 0.711 | | ± | 0.223 |  | 0.589 | | ± | 0.223** | |
| **de18:1/16:0 Cer** |  | | 2.569 | | ± | 1.080 |  | 1.932 | | ± | 0.962**** | |
| **de18:1/18:0 Cer** |  | | 3.305 | | ± | 1.891 |  | 2.696 | | ± | 1.667** | |
| **de18:1/20:0 Cer** |  | | 9.335 | | ± | 3.983 |  | 8.481 | | ± | 4.115** | |
| **de18:1/22:0 Cer** |  | | 63.652 | | ± | 29.611 |  | 55.736 | | ± | 25.471* | |
| **de18:1/24:1 Cer** |  | | 47.217 | | ± | 16.042 |  | 49.952 | | ± | 19.464** | |
| **de18:1/24:0 Cer** |  | | 168.034 | | ± | 77.278 |  | 141.957 | | ± | 60.240* | |
| **de18:1/26:1 Cer** |  | | 1.839 | | ± | 0.536 |  | 2.611 | | ± | 0.919**** | |
| **de18:1/26:0 Cer** |  | | 2.275 | | ± | 1.063 |  | 1.990 | | ± | 1.156* | |
| **de18:1/14:0 C1P** |  | | 0.921 | | ± | 0.391 |  | 0.716 | | ± | 0.252 | |
| **de18:1/16:0 C1P** |  | | 55.621 | | ± | 20.155 |  | 41.244 | | ± | 20.379* | |
| **de18:1/22:0 C1P** |  | | 0.951 | | ± | 0.726 |  | 0.965 | | ± | 0.332**** | |
| **de18:1/24:1 C1P** |  | | 1.876 | | ± | 0.868 |  | 1.438 | | ± | 1.103**** | |
| **de18:1/24:0 C1P** |  | | 4.421 | | ± | 1.942 |  | 3.415 | | ± | 2.112**** | |
| **de18:1/14:0 SM** |  | | 97.654 | | ± | 36.269 |  | 75.046 | | ± | 40.097* | |
| **de18:1/16:0 SM** |  | | 83.263 | | ± | 33.211 |  | 61.950 | | ± | 37.585**** | |
| **de18:1/18:1 SM** |  | | 165.409 | | ± | 64.728 |  | 126.689 | | ± | 71.956* | |
| **de18:1/18:0 SM** |  | | 1.466 | | ± | 0.318 |  | 1.484 | | ± | 0.477**** | |
| **de18:1/20:0 SM** |  | | 1.485 | | ± | 0.691 |  | 1.216 | | ± | 0.940** | |
| **de18:1/22:0 SM** |  | | 287.493 | | ± | 35.351 |  | 283.894 | | ± | 34.920 | |
| **de18:1/24:1 SM** |  | | 262.125 | | ± | 81.104 |  | 235.451 | | ± | 89.468** | |
| **de18:1/24:0 SM** |  | | 205.154 | | ± | 36.712 |  | 177.906 | | ± | 46.885* | |
| **de18:1/26:1 SM** |  | | 302.781 | | ± | 47.680 |  | 294.056 | | ± | 43.333** | |
| **de18:1/26:0 SM** |  | | 390.526 | | ± | 39.575 |  | 375.261 | | ± | 40.666**** | |
| **de18:1/14:0 MonHex** |  | | 539.834 | | ± | 91.772 |  | 551.843 | | ± | 59.404** | |
| **de18:1/16:0 MonHex** |  | | 546.530 | | ± | 115.308 |  | 524.659 | | ± | 136.667**** | |
| **de18:1/18:1 MonHex** |  | | 584.941 | | ± | 144.885 |  | 549.928 | | ± | 111.954**** | |
| **de18:1/18:0 MonHex** |  | | 30.257 | | ± | 9.112 |  | 26.029 | | ± | 8.774* | |
| **de18:1/20:0 MonHex** |  | | 9.851 | | ± | 4.287 |  | 8.373 | | ± | 3.524**** | |
| **de18:1/22:0 MonHex** |  | | 0.233 | | ± | 0.148 |  | 0.165 | | ± | 0.037**** | |
| **de18:1/24:1 MonHex** |  | | 4.691 | | ± | 1.869 |  | 3.550 | | ± | 1.299**** | |
| **de18:1/24:0 MonHex** |  | | 1.748 | | ± | 0.705 |  | 1.203 | | ± | 0.526**** | |
| **de18:1/26:1 MonHex** |  | | 3.798 | | ± | 1.502 |  | 2.148 | | ± | 1.526**** | |
| **de18:1/26:0 MonHex** |  | | 2.432 | | ± | 1.014 |  | 1.736 | | ± | 0.803** | |
| **So** |  | | 1.459 | | ± | 1.302 |  | 1.311 | | ± | 0.858 | |
| **S1P** |  | | 44.116 | | ± | 17.025 |  | 43.352 | | ± | 12.843 | |
| **Sa1P** |  | | 7.212 | | ± | 3.123 |  | 7.079 | | ± | 2.435 | |

**Supplemental Table 7. Sphingolipid profile observed in subjects with uncomplicated term and PE pregnancies with a preterm birth.** Raw, non-transformed sphingolipid levels observed in patients with uncomplicated pregnancies vs. patients later diagnosed with PE and a preterm birth. Samples were taken prior to 24 weeks gestation and analyzed by UPLC ESI-M/S within two weeks of acquisition. Raw data were statistically compared using the Wilcoxon Sum Rank Test. The data presented are in Means ± SD in pmol lipid/50 µL plasma. Significance is represented as *p <0.05; **p<0.01; ***p<0.001; ****p<0.0001.

| **Analyte** | |  | | **Uncomplicated (n=31) pmol/50µL Plasma** | | |  | | **Term Preeclamptic (n=14)**  **pmol/50µL Plasma** | | |  |
| --- | --- | --- | --- | --- | --- | --- | --- | --- | --- | --- | --- | --- |
| **de18:1/14:0 Cer** |  | | 0.711 | | ± | 0.223 |  | 0.583 | | ± | 0.229** | |
| **de18:1/16:0 Cer** |  | | 2.569 | | ± | 1.080 |  | 1.830 | | ± | 0.914** | |
| **de18:1/18:0 Cer** |  | | 3.305 | | ± | 1.891 |  | 2.637 | | ± | 1.710* | |
| **de18:1/20:0 Cer** |  | | 9.335 | | ± | 3.983 |  | 8.445 | | ± | 4.257* | |
| **de18:1/22:0 Cer** |  | | 63.652 | | ± | 29.611 |  | 56.610 | | ± | 26.147 | |
| **de18:1/24:1 Cer** |  | | 47.217 | | ± | 16.042 |  | 50.636 | | ± | 19.972 | |
| **de18:1/24:0 Cer** |  | | 168.034 | | ± | 77.278 |  | 143.204 | | ± | 62.167* | |
| **de18:1/26:1 Cer** |  | | 1.839 | | ± | 0.536 |  | 2.719 | | ± | 0.853**** | |
| **de18:1/26:0 Cer** |  | | 2.275 | | ± | 1.063 |  | 2.044 | | ± | 1.179** | |
| **de18:1/14:0 C1P** |  | | 0.921 | | ± | 0.391 |  | 0.719 | | ± | 0.261 | |
| **de18:1/16:0 C1P** |  | | 55.621 | | ± | 20.155 |  | 40.042 | | ± | 20.575** | |
| **de18:1/22:0 C1P** |  | | 0.951 | | ± | 0.726 |  | 0.996 | | ± | 0.323**** | |
| **de18:1/24:1 C1P** |  | | 1.876 | | ± | 0.868 |  | 1.351 | | ± | 1.092**** | |
| **de18:1/24:0 C1P** |  | | 4.421 | | ± | 1.942 |  | 3.202 | | ± | 2.025**** | |
| **de18:1/14:0 SM** |  | | 97.654 | | ± | 36.269 |  | 69.246 | | ± | 34.899 | |
| **de18:1/16:0 SM** |  | | 83.263 | | ± | 33.211 |  | 59.112 | | ± | 37.318**** | |
| **de18:1/18:1 SM** |  | | 165.409 | | ± | 64.728 |  | 126.577 | | ± | 74.480**** | |
| **de18:1/18:0 SM** |  | | 1.466 | | ± | 0.318 |  | 1.466 | | ± | 0.489* | |
| **de18:1/20:0 SM** |  | | 1.485 | | ± | 0.691 |  | 1.260 | | ± | 0.957* | |
| **de18:1/22:0 SM** |  | | 287.493 | | ± | 35.351 |  | 279.924 | | ± | 32.712 | |
| **de18:1/24:1 SM** |  | | 262.125 | | ± | 81.104 |  | 233.660 | | ± | 92.348* | |
| **de18:1/24:0 SM** |  | | 205.154 | | ± | 36.712 |  | 173.634 | | ± | 45.623* | |
| **de18:1/26:1 SM** |  | | 302.781 | | ± | 47.680 |  | 290.123 | | ± | 42.188**** | |
| **de18:1/26:0 SM** |  | | 390.526 | | ± | 39.575 |  | 369.986 | | ± | 36.801* | |
| **de18:1/14:0 MonHex** |  | | 539.834 | | ± | 91.772 |  | 543.015 | | ± | 51.106 | |
| **de18:1/16:0 MonHex** |  | | 546.530 | | ± | 115.308 |  | 523.350 | | ± | 141.373* | |
| **de18:1/18:1 MonHex** |  | | 584.941 | | ± | 144.885 |  | 542.467 | | ± | 112.222 | |
| **de18:1/18:0 MonHex** |  | | 30.257 | | ± | 9.112 |  | 26.366 | | ± | 8.988* | |
| **de18:1/20:0 MonHex** |  | | 9.851 | | ± | 4.287 |  | 8.547 | | ± | 3.585* | |
| **de18:1/22:0 MonHex** |  | | 0.233 | | ± | 0.148 |  | 0.167 | | ± | 0.038* | |
| **de18:1/24:1 MonHex** |  | | 4.691 | | ± | 1.869 |  | 3.568 | | ± | 1.343* | |
| **de18:1/24:0 MonHex** |  | | 1.748 | | ± | 0.705 |  | 1.199 | | ± | 0.544 | |
| **de18:1/26:1 MonHex** |  | | 3.798 | | ± | 1.502 |  | 2.302 | | ± | 1.464 | |
| **de18:1/26:0 MonHex** |  | | 2.432 | | ± | 1.014 |  | 1.802 | | ± | 0.791 | |
| **So** |  | | 1.459 | | ± | 1.302 |  | 1.371 | | ± | 0.857 | |
| **S1P** |  | | 44.116 | | ± | 17.025 |  | 42.481 | | ± | 12.858 | |
| **Sa1P** |  | | 7.212 | | ± | 3.123 |  | 6.908 | | ± | 2.432 | |

**Supplemental Table 8. Sphingolipid profile observed in subjects with uncomplicated term and PE pregnancies with a term birth.** Raw, non-transformed sphingolipid levels observed in patients with uncomplicated pregnancies vs. patients later diagnosed with PE and a term birth. Samples were taken prior to 24 weeks gestation and analyzed by UPLC ESI-M/S within two weeks of acquisition. Raw data were statistically compared using the Wilcoxon Sum Rank Test. The data presented are in Means ± SD in pmol lipid/50 µL plasma. Significance is represented as *p <0.05; **p<0.01; ***p<0.001; ****p<0.0001.

| **Analyte** |  | **Uncomplicated (n=19)** | | | |  |  | **Preeclamptic (n=13)** | | |
| --- | --- | --- | --- | --- | --- | --- | --- | --- | --- | --- |
|  |  | **pmol/100 µL Plasma** | | | |  |  | **pmol/100 µL Plasma** | | |
| **6-keto PGF1α** |  | 0.009 | ± | 0.005 |  | |  | 0.006 | ± | 0.002 |
| **TXB_2_** |  | 0.280 | ± | 0.246 |  | |  | 0.143 | ± | 0.167* |
| **PGE_2_** |  | 0.032 | ± | 0.026 |  | |  | 0.017 | ± | 0.014 |
| **PGD_2_** |  | 0.031 | ± | 0.028 |  | |  | 0.025 | ± | 0.023 |
| **Resolvin D1** |  | 0.128 | ± | 0.018 |  | |  | 0.133 | ± | 0.020 |
| **PGA_2_** |  | 0.238 | ± | 0.028 |  | |  | 0.239 | ± | 0.026 |
| **(±)14,15 DHET** |  | 0.116 | ± | 0.047 |  | |  | 0.103 | ± | 0.027 |
| **Maresin 2** |  | 0.009 | ± | 0.004 |  | |  | 0.012 | ± | 0.005 |
| **(±)11,12 DHET** |  | 0.123 | ± | 0.056 |  | |  | 0.101 | ± | 0.024* |
| **(±) 8,9 DHET** |  | 0.032 | ± | 0.015 |  | |  | 0.026 | ± | 0.004 |
| **20-HETE** |  | 9.222 | ± | 4.927 |  | |  | 8.740 | ± | 2.966 |
| **15-HETE** |  | 0.115 | ± | 0.075 |  | |  | 0.088 | ± | 0.033 |
| **12-HETE** |  | 0.635 | ± | 0.775 |  | |  | 0.331 | ± | 0.240 |
| **(±)14,15 EET** |  | 0.040 | ± | 0.055 |  | |  | 0.027 | ± | 0.012 |
| **5-HETE** |  | 0.186 | ± | 0.141 |  | |  | 0.145 | ± | 0.094 |
| **EPA** |  | 14.488 | ± | 7.448 |  | |  | 18.142 | ± | 9.516 |
| **DHA** |  | 114.723 | ± | 50.432 |  | |  | 141.320 | ± | 50.386 |
| **AA** |  | 514.251 | ± | 244.561 |  | |  | 560.996 | ± | 216.431 |
| **DHGLA** |  | 84.076 | ± | 39.556 |  | |  | 98.843 | ± | 69.561 |

**Supplemental Table 9. Eicosanoid profile observed in uncomplicated term pregnant patients vs. PE pregnancies of African descent.** Raw, non-transformed eicosanoid levels observed in patients of African descent with uncomplicated pregnancies vs. patients later diagnosed with PE. Samples were taken prior to 24 weeks gestation and analyzed by UPLC ESI-M/S within two weeks of acquisition. Raw data were statistically compared using the Wilcoxon Sum Rank Test. The data presented are in Means ± SD in pmol lipid/100 µL plasma. Significance is represented as *p <0.05; **p<0.01; ***p<0.001; ****p<0.0001.

| **Analyte** | |  | | **Uncomplicated (n=19) pmol/50µL Plasma** | | |  | | **Preeclamptic (n=13)**  **pmol/50µL Plasma** | | |  |
| --- | --- | --- | --- | --- | --- | --- | --- | --- | --- | --- | --- | --- |
| **de18:1/14:0 Cer** |  | | 0.679 | | ± | 0.224 |  | 0.486 | | ± | 0.163* | |
| **de18:1/16:0 Cer** |  | | 2.605 | | ± | 1.023 |  | 1.637 | | ± | 0.770** | |
| **de18:1/18:0 Cer** |  | | 3.707 | | ± | 1.986 |  | 2.276 | | ± | 1.357* | |
| **de18:1/20:0 Cer** |  | | 9.821 | | ± | 3.866 |  | 6.615 | | ± | 2.520* | |
| **de18:1/22:0 Cer** |  | | 64.957 | | ± | 28.451 |  | 49.212 | | ± | 17.846 | |
| **de18:1/24:1 Cer** |  | | 46.581 | | ± | 15.707 |  | 41.060 | | ± | 15.415 | |
| **de18:1/24:0 Cer** |  | | 167.083 | | ± | 77.207 |  | 123.374 | | ± | 48.574 | |
| **de18:1/26:1 Cer** |  | | 1.709 | | ± | 0.362 |  | 2.546 | | ± | 0.910** | |
| **de18:1/26:0 Cer** |  | | 2.187 | | ± | 0.899 |  | 1.536 | | ± | 0.714* | |
| **de18:1/14:0 C1P** |  | | 0.926 | | ± | 0.407 |  | 0.623 | | ± | 0.208 | |
| **de18:1/16:0 C1P** |  | | 61.008 | | ± | 21.255 |  | 39.389 | | ± | 19.449 | |
| **de18:1/22:0 C1P** |  | | 1.029 | | ± | 0.849 |  | 1.280 | | ± | 0.559 | |
| **de18:1/24:1 C1P** |  | | 2.086 | | ± | 0.909 |  | 1.194 | | ± | 1.075** | |
| **de18:1/24:0 C1P** |  | | 4.833 | | ± | 2.072 |  | 2.742 | | ± | 1.744 | |
| **de18:1/14:0 SM** |  | | 104.964 | | ± | 37.873 |  | 64.860 | | ± | 26.651 | |
| **de18:1/16:0 SM** |  | | 88.822 | | ± | 36.203 |  | 48.711 | | ± | 30.398* | |
| **de18:1/18:1 SM** |  | | 180.029 | | ± | 65.236 |  | 108.250 | | ± | 53.826* | |
| **de18:1/18:0 SM** |  | | 1.469 | | ± | 0.350 |  | 1.420 | | ± | 0.363 | |
| **de18:1/20:0 SM** |  | | 1.635 | | ± | 0.740 |  | 1.024 | | ± | 0.509 | |
| **de18:1/22:0 SM** |  | | 288.405 | | ± | 28.685 |  | 275.408 | | ± | 33.585 | |
| **de18:1/24:1 SM** |  | | 265.427 | | ± | 86.939 |  | 218.148 | | ± | 73.389 | |
| **de18:1/24:0 SM** |  | | 213.922 | | ± | 35.139 |  | 173.380 | | ± | 53.902 | |
| **de18:1/26:1 SM** |  | | 310.407 | | ± | 46.782 |  | 299.170 | | ± | 52.343* | |
| **de18:1/26:0 SM** |  | | 387.091 | | ± | 39.468 |  | 365.674 | | ± | 42.979 | |
| **de18:1/14:0 MonHex** |  | | 523.028 | | ± | 95.528 |  | 515.423 | | ± | 83.597* | |
| **de18:1/16:0 MonHex** |  | | 541.130 | | ± | 124.558 |  | 489.879 | | ± | 118.066* | |
| **de18:1/18:1 MonHex** |  | | 574.582 | | ± | 144.896 |  | 511.040 | | ± | 106.655 | |
| **de18:1/18:0 MonHex** |  | | 30.326 | | ± | 7.534 |  | 23.947 | | ± | 7.358** | |
| **de18:1/20:0 MonHex** |  | | 10.137 | | ± | 4.348 |  | 7.938 | | ± | 3.186** | |
| **de18:1/22:0 MonHex** |  | | 0.244 | | ± | 0.174 |  | 0.172 | | ± | 0.059* | |
| **de18:1/24:1 MonHex** |  | | 4.541 | | ± | 1.906 |  | 3.780 | | ± | 1.373** | |
| **de18:1/24:0 MonHex** |  | | 1.712 | | ± | 0.759 |  | 1.290 | | ± | 0.447* | |
| **de18:1/26:1 MonHex** |  | | 3.700 | | ± | 1.654 |  | 2.071 | | ± | 1.137 | |
| **de18:1/26:0 MonHex** |  | | 2.331 | | ± | 1.004 |  | 1.783 | | ± | 0.487* | |
| **So** |  | | 1.433 | | ± | 1.156 |  | 1.075 | | ± | 0.525 | |
| **S1P** |  | | 44.199 | | ± | 18.572 |  | 37.569 | | ± | 6.412 | |
| **Sa1P** |  | | 7.224 | | ± | 3.285 |  | 6.189 | | ± | 1.874 | |

**Supplemental Table 10. Sphingolipid profile observed in subjects with uncomplicated term and PE pregnancies of African descent.** Raw, non-transformed sphingolipid levels observed in patients of African descent with uncomplicated pregnancies vs. patients later diagnosed with PE and a term birth. Samples were taken prior to 24 weeks gestation and analyzed by UPLC ESI-M/S within two weeks of acquisition. Raw data were statistically compared using the Wilcoxon Sum Rank Test. The data presented are in Means ± SD in pmol lipid/50 µL plasma. Significance is represented as *p <0.05; **p<0.01; ***p<0.001; ****p<0.0001.

| **Analyte** |  | **Uncomplicated (n=7)** | | | |  |  | **Preeclamptic (n=5)** | | |
| --- | --- | --- | --- | --- | --- | --- | --- | --- | --- | --- |
|  |  | **pmol/100 µL Plasma** | | | |  |  | **pmol/100 µL Plasma** | | |
| **6-keto PGF1α** |  | 0.006 | ± | 0.002 |  | |  | 0.004 | ± | 0.000 |
| **TXB_2_** |  | 0.197 | ± | 0.133 |  | |  | 0.133 | ± | 0.158 |
| **PGE_2_** |  | 0.025 | ± | 0.026 |  | |  | 0.012 | ± | 0.009 |
| **PGD_2_** |  | 0.032 | ± | 0.016 |  | |  | 0.023 | ± | 0.015 |
| **Resolvin D1** |  | 0.122 | ± | 0.003 |  | |  | 0.126 | ± | 0.004 |
| **PGA_2_** |  | 0.228 | ± | 0.010 |  | |  | 0.223 | ± | 0.004 |
| **(±)14,15 DHET** |  | 0.101 | ± | 0.029 |  | |  | 0.086 | ± | 0.032 |
| **Maresin 2** |  | 0.009 | ± | 0.002 |  | |  | 0.008 | ± | 0.002 |
| **(±)11,12 DHET** |  | 0.103 | ± | 0.033 |  | |  | 0.075 | ± | 0.024 |
| **(±) 8,9 DHET** |  | 0.030 | ± | 0.012 |  | |  | 0.028 | ± | 0.010 |
| **20-HETE** |  | 7.931 | ± | 3.367 |  | |  | 8.212 | ± | 2.138 |
| **15-HETE** |  | 0.153 | ± | 0.114 |  | |  | 0.060 | ± | 0.032* |
| **12-HETE** |  | 1.093 | ± | 1.331 |  | |  | 0.209 | ± | 0.299* |
| **(±)14,15 EET** |  | 0.023 | ± | 0.012 |  | |  | 0.016 | ± | 0.005 |
| **5-HETE** |  | 0.163 | ± | 0.081 |  | |  | 0.095 | ± | 0.021 |
| **EPA** |  | 18.584 | ± | 4.778 |  | |  | 8.045 | ± | 3.217** |
| **DHA** |  | 129.446 | ± | 32.810 |  | |  | 79.231 | ± | 22.762* |
| **AA** |  | 364.012 | ± | 104.353 |  | |  | 249.892 | ± | 61.353 |
| **DHGLA** |  | 73.236 | ± | 25.668 |  | |  | 41.299 | ± | 17.342 |

**Supplemental Table 11. Eicosanoid profile observed in uncomplicated term pregnant patients vs. PE pregnancies of Western European descent.** Raw, non-transformed eicosanoid levels observed in patients of Western European descent with uncomplicated pregnancies vs. patients later diagnosed with PE. Samples were taken prior to 24 weeks gestation and analyzed by UPLC ESI-M/S within two weeks of acquisition. Raw data were statistically compared using the Wilcoxon Sum Rank Test. The data presented are in Means ± SD in pmol lipid/100 µL plasma. Significance is represented as *p <0.05; **p<0.01; ***p<0.001; ****p<0.0001.

| **Analyte** | |  | | **Uncomplicated (n=7) pmol/50µL Plasma** | | |  | | **Preeclamptic (n=5)**  **pmol/50µL Plasma** | | |  |
| --- | --- | --- | --- | --- | --- | --- | --- | --- | --- | --- | --- | --- |
| **de18:1/14:0 Cer** |  | | 0.795 | | ± | 0.194 |  | 0.576 | | ± | 0.242 | |
| **de18:1/16:0 Cer** |  | | 3.089 | | ± | 1.027 |  | 1.998 | | ± | 0.992 | |
| **de18:1/18:0 Cer** |  | | 3.070 | | ± | 1.529 |  | 2.290 | | ± | 1.233 | |
| **de18:1/20:0 Cer** |  | | 10.173 | | ± | 4.083 |  | 8.673 | | ± | 5.834 | |
| **de18:1/22:0 Cer** |  | | 76.170 | | ± | 30.362 |  | 53.535 | | ± | 35.707 | |
| **de18:1/24:1 Cer** |  | | 52.884 | | ± | 17.545 |  | 48.517 | | ± | 24.962 | |
| **de18:1/24:0 Cer** |  | | 204.243 | | ± | 77.559 |  | 142.761 | | ± | 81.243 | |
| **de18:1/26:1 Cer** |  | | 1.764 | | ± | 0.585 |  | 2.316 | | ± | 0.833 | |
| **de18:1/26:0 Cer** |  | | 2.900 | | ± | 1.377 |  | 2.077 | | ± | 1.773 | |
| **de18:1/14:0 C1P** |  | | 0.978 | | ± | 0.431 |  | 0.865 | | ± | 0.268 | |
| **de18:1/16:0 C1P** |  | | 53.798 | | ± | 10.868 |  | 39.454 | | ± | 19.659 | |
| **de18:1/22:0 C1P** |  | | 0.667 | | ± | 0.401 |  | 0.815 | | ± | 0.216* | |
| **de18:1/24:1 C1P** |  | | 1.760 | | ± | 0.553 |  | 1.500 | | ± | 0.977* | |
| **de18:1/24:0 C1P** |  | | 4.501 | | ± | 0.914 |  | 3.845 | | ± | 2.466* | |
| **de18:1/14:0 SM** |  | | 102.696 | | ± | 20.037 |  | 89.043 | | ± | 51.624 | |
| **de18:1/16:0 SM** |  | | 84.884 | | ± | 20.422 |  | 70.884 | | ± | 44.213 | |
| **de18:1/18:1 SM** |  | | 166.201 | | ± | 50.148 |  | 130.570 | | ± | 98.399 | |
| **de18:1/18:0 SM** |  | | 1.526 | | ± | 0.216 |  | 1.521 | | ± | 0.587 | |
| **de18:1/20:0 SM** |  | | 1.416 | | ± | 0.528 |  | 1.319 | | ± | 1.439 | |
| **de18:1/22:0 SM** |  | | 288.405 | | ± | 28.685 |  | 275.408 | | ± | 33.585 | |
| **de18:1/24:1 SM** |  | | 265.427 | | ± | 86.939 |  | 218.148 | | ± | 73.389 | |
| **de18:1/24:0 SM** |  | | 213.922 | | ± | 35.139 |  | 173.380 | | ± | 53.902 | |
| **de18:1/26:1 SM** |  | | 310.407 | | ± | 46.782 |  | 299.170 | | ± | 52.343* | |
| **de18:1/26:0 SM** |  | | 387.091 | | ± | 39.468 |  | 365.674 | | ± | 42.979 | |
| **de18:1/14:0 MonHex** |  | | 523.028 | | ± | 95.528 |  | 515.423 | | ± | 83.597 | |
| **de18:1/16:0 MonHex** |  | | 541.130 | | ± | 124.558 |  | 489.879 | | ± | 118.066* | |
| **de18:1/18:1 MonHex** |  | | 574.582 | | ± | 144.896 |  | 511.040 | | ± | 106.655 | |
| **de18:1/18:0 MonHex** |  | | 30.326 | | ± | 7.534 |  | 23.947 | | ± | 7.358 | |
| **de18:1/20:0 MonHex** |  | | 10.137 | | ± | 4.348 |  | 7.938 | | ± | 3.186 | |
| **de18:1/22:0 MonHex** |  | | 0.244 | | ± | 0.174 |  | 0.172 | | ± | 0.059 | |
| **de18:1/24:1 MonHex** |  | | 4.541 | | ± | 1.906 |  | 3.780 | | ± | 1.373 | |
| **de18:1/24:0 MonHex** |  | | 1.712 | | ± | 0.759 |  | 1.290 | | ± | 0.447 | |
| **de18:1/26:1 MonHex** |  | | 3.700 | | ± | 1.654 |  | 2.071 | | ± | 1.137 | |
| **de18:1/26:0 MonHex** |  | | 2.331 | | ± | 1.004 |  | 1.783 | | ± | 0.487 | |
| **So** |  | | 1.433 | | ± | 1.156 |  | 1.075 | | ± | 0.525 | |
| **S1P** |  | | 44.199 | | ± | 18.572 |  | 37.569 | | ± | 6.412 | |
| **Sa1P** |  | | 7.224 | | ± | 3.285 |  | 6.189 | | ± | 1.874 | |

**Supplemental Table 12. Sphingolipid profile observed in subjects with uncomplicated term and PE pregnancies of Western European descent.** Raw, non-transformed sphingolipid levels observed in patients of Western European descent with uncomplicated pregnancies vs. patients later diagnosed with PE and a term birth. Samples were taken prior to 24 weeks gestation and analyzed by UPLC ESI-M/S within two weeks of acquisition. Raw data were statistically compared using the Wilcoxon Sum Rank Test. The data presented are in Means ± SD in pmol lipid/50 µL plasma. Significance is represented as *p <0.05; **p<0.01; ***p<0.001; ****p<0.0001.

| **Analyte** |  | **Uncomplicated (n=4)** | | | |  |  | **Preeclamptic (n=8)** | | |
| --- | --- | --- | --- | --- | --- | --- | --- | --- | --- | --- |
|  |  | **pmol/100 µL Plasma** | | | |  |  | **pmol/100 µL Plasma** | | |
| **6-keto PGF1α** |  | 0.004 | ± | 0.001 |  | |  | 0.008 | ± | 0.002 |
| **TXB_2_** |  | 0.063 | ± | 0.029 |  | |  | 0.234 | ± | 0.213 |
| **PGE_2_** |  | 0.012 | ± | 0.002 |  | |  | 0.023 | ± | 0.013 |
| **PGD_2_** |  | 0.018 | ± | 0.004 |  | |  | 0.056 | ± | 0.040 |
| **Resolvin D1** |  | 0.127 | ± | 0.006 |  | |  | 0.129 | ± | 0.005 |
| **PGA_2_** |  | 0.212 | ± | 0.006 |  | |  | 0.227 | ± | 0.007* |
| **(±)14,15 DHET** |  | 0.090 | ± | 0.021 |  | |  | 0.079 | ± | 0.023 |
| **Maresin 2** |  | 0.009 | ± | 0.005 |  | |  | 0.007 | ± | 0.003 |
| **(±)11,12 DHET** |  | 0.096 | ± | 0.028 |  | |  | 0.076 | ± | 0.019 |
| **(±) 8,9 DHET** |  | 0.020 | ± | 0.002 |  | |  | 0.022 | ± | 0.007 |
| **20-HETE** |  | 12.787 | ± | 5.372 |  | |  | 11.194 | ± | 3.265 |
| **15-HETE** |  | 0.096 | ± | 0.030 |  | |  | 0.076 | ± | 0.025 |
| **12-HETE** |  | 0.181 | ± | 0.084 |  | |  | 0.646 | ± | 0.639 |
| **(±)14,15 EET** |  | 0.015 | ± | 0.005 |  | |  | 0.022 | ± | 0.013 |
| **5-HETE** |  | 0.135 | ± | 0.072 |  | |  | 0.131 | ± | 0.063 |
| **EPA** |  | 13.761 | ± | 5.203 |  | |  | 15.601 | ± | 8.213 |
| **DHA** |  | 137.995 | ± | 37.894 |  | |  | 156.643 | ± | 73.767 |
| **AA** |  | 468.976 | ± | 155.291 |  | |  | 458.390 | ± | 199.709 |
| **DHGLA** |  | 78.424 | ± | 34.125 |  | |  | 87.032 | ± | 45.457 |

**Supplemental Table 13. Eicosanoid profile observed in uncomplicated term pregnant patients vs. PE pregnancies of Hispanic descent.** Raw, non-transformed eicosanoid levels observed in patients of Hispanic descent with uncomplicated pregnancies vs. patients later diagnosed with PE. Samples were taken prior to 24 weeks gestation and analyzed by UPLC ESI-M/S within two weeks of acquisition. Raw data were statistically compared using the Wilcoxon Sum Rank Test. The data presented are in Means ± SD in pmol lipid/100 µL plasma. Significance is represented as *p <0.05; **p<0.01; ***p<0.001; ****p<0.0001.

| **Analyte** | |  | | **Uncomplicated (n=4) pmol/50µL Plasma** | | |  | | **Preeclamptic (n=8)**  **pmol/50µL Plasma** | | |  |
| --- | --- | --- | --- | --- | --- | --- | --- | --- | --- | --- | --- | --- |
| **de18:1/14:0 Cer** |  | | 0.713 | | ± | 0.225 |  | 0.572 | | ± | 0.256 | |
| **de18:1/16:0 Cer** |  | | 1.490 | | ± | 0.529 |  | 1.362 | | ± | 0.712 | |
| **de18:1/18:0 Cer** |  | | 1.809 | | ± | 0.934 |  | 2.029 | | ± | 1.599 | |
| **de18:1/20:0 Cer** |  | | 5.555 | | ± | 1.472 |  | 6.605 | | ± | 3.152 | |
| **de18:1/22:0 Cer** |  | | 35.545 | | ± | 6.761 |  | 41.514 | | ± | 15.277 | |
| **de18:1/24:1 Cer** |  | | 40.320 | | ± | 10.676 |  | 41.901 | | ± | 20.056 | |
| **de18:1/24:0 Cer** |  | | 109.185 | | ± | 13.620 |  | 106.702 | | ± | 24.613 | |
| **de18:1/26:1 Cer** |  | | 2.589 | | ± | 0.531 |  | 2.637 | | ± | 0.451 | |
| **de18:1/26:0 Cer** |  | | 1.597 | | ± | 0.371 |  | 1.483 | | ± | 0.458 | |
| **de18:1/14:0 C1P** |  | | 0.797 | | ± | 0.127 |  | 0.566 | | ± | 0.286 | |
| **de18:1/16:0 C1P** |  | | 33.222 | | ± | 6.984 |  | 24.165 | | ± | 7.300 | |
| **de18:1/22:0 C1P** |  | | 1.078 | | ± | 0.243 |  | 1.008 | | ± | 0.241* | |
| **de18:1/24:1 C1P** |  | | 1.083 | | ± | 0.575 |  | 0.745 | | ± | 0.302* | |
| **de18:1/24:0 C1P** |  | | 2.323 | | ± | 1.094 |  | 1.852 | | ± | 0.920* | |
| **de18:1/14:0 SM** |  | | 54.107 | | ± | 12.467 |  | 39.906 | | ± | 12.821 | |
| **de18:1/16:0 SM** |  | | 54.024 | | ± | 17.618 |  | 37.112 | | ± | 16.738 | |
| **de18:1/18:1 SM** |  | | 94.575 | | ± | 27.806 |  | 69.727 | | ± | 27.117 | |
| **de18:1/18:0 SM** |  | | 1.348 | | ± | 0.268 |  | 0.975 | | ± | 0.340 | |
| **de18:1/20:0 SM** |  | | 0.895 | | ± | 0.168 |  | 0.621 | | ± | 0.301 | |
| **de18:1/22:0 SM** |  | | 267.041 | | ± | 1.238 |  | 258.899 | | ± | 12.002 | |
| **de18:1/24:1 SM** |  | | 260.232 | | ± | 108.762 |  | 216.805 | | ± | 88.181 | |
| **de18:1/24:0 SM** |  | | 165.424 | | ± | 30.857 |  | 139.959 | | ± | 29.466 | |
| **de18:1/26:1 SM** |  | | 254.815 | | ± | 42.854 |  | 249.880 | | ± | 39.145* | |
| **de18:1/26:0 SM** |  | | 364.280 | | ± | 45.933 |  | 344.080 | | ± | 35.854 | |
| **de18:1/14:0 MonHex** |  | | 512.617 | | ± | 71.017 |  | 522.359 | | ± | 51.117 | |
| **de18:1/16:0 MonHex** |  | | 622.986 | | ± | 120.811 |  | 528.131 | | ± | 122.375* | |
| **de18:1/18:1 MonHex** |  | | 468.462 | | ± | 40.828 |  | 445.906 | | ± | 50.119 | |
| **de18:1/18:0 MonHex** |  | | 25.645 | | ± | 1.483 |  | 20.567 | | ± | 3.323 | |
| **de18:1/20:0 MonHex** |  | | 7.174 | | ± | 1.697 |  | 6.294 | | ± | 1.462 | |
| **de18:1/22:0 MonHex** |  | | 0.211 | | ± | 0.062 |  | 0.184 | | ± | 0.040 | |
| **de18:1/24:1 MonHex** |  | | 4.107 | | ± | 1.647 |  | 2.555 | | ± | 0.833 | |
| **de18:1/24:0 MonHex** |  | | 1.171 | | ± | 0.056 |  | 0.697 | | ± | 0.269 | |
| **de18:1/26:1 MonHex** |  | | 2.875 | | ± | 0.956 |  | 1.284 | | ± | 0.670 | |
| **de18:1/26:0 MonHex** |  | | 1.888 | | ± | 0.159 |  | 1.074 | | ± | 0.373 | |
| **So** |  | | 1.045 | | ± | 0.404 |  | 1.429 | | ± | 0.581 | |
| **S1P** |  | | 33.294 | | ± | 6.886 |  | 45.475 | | ± | 10.232 | |
| **Sa1P** |  | | 5.709 | | ± | 1.709 |  | 7.141 | | ± | 0.850 | |

**Supplemental Table 14. Sphingolipid profile observed in subjects with uncomplicated term and PE pregnancies of Hispanic descent.** Raw, non-transformed sphingolipid levels observed in patients of Hispanic descent with uncomplicated pregnancies vs. patients later diagnosed with PE and a term birth. Samples were taken prior to 24 weeks gestation and analyzed by UPLC ESI-M/S within two weeks of acquisition. Raw data were statistically compared using the Wilcoxon Sum Rank Test. The data presented are in Means ± SD in pmol lipid/50 µL plasma. Significance is represented as *p <0.05; **p<0.01; ***p<0.001; ****p<0.0001.

| **Analyte** |  | **Uncomplicated (n=16)** | | | |  |  | **Preeclamptic (n=26)** | | |
| --- | --- | --- | --- | --- | --- | --- | --- | --- | --- | --- |
|  |  | **pmol/100 µL Plasma** | | | |  |  | **pmol/100 µL Plasma** | | |
| **6-keto PGF1α** |  | 0.007 | ± | 0.002 |  | |  | 0.007 | ± | 0.002 |
| **TXB_2_** |  | 0.268 | ± | 0.239 |  | |  | 0.169 | ± | 0.186 |
| **PGE_2_** |  | 0.032 | ± | 0.027 |  | |  | 0.018 | ± | 0.013 |
| **PGD_2_** |  | 0.029 | ± | 0.027 |  | |  | 0.037 | ± | 0.034 |
| **Resolvin D1** |  | 0.120 | ± | 0.003 |  | |  | 0.131 | ± | 0.015**** |
| **PGA_2_** |  | 0.230 | ± | 0.009 |  | |  | 0.232 | ± | 0.020 |
| **(±)14,15 DHET** |  | 0.098 | ± | 0.022 |  | |  | 0.092 | ± | 0.029 |
| **Maresin 2** |  | 0.009 | ± | 0.003 |  | |  | 0.010 | ± | 0.005 |
| **(±)11,12 DHET** |  | 0.101 | ± | 0.027 |  | |  | 0.088 | ± | 0.026 |
| **(±) 8,9 DHET** |  | 0.030 | ± | 0.010 |  | |  | 0.025 | ± | 0.007 |
| **20-HETE** |  | 6.238 | ± | 0.971 |  | |  | 9.394 | ± | 3.168**** |
| **15-HETE** |  | 0.122 | ± | 0.101 |  | |  | 0.079 | ± | 0.032 |
| **12-HETE** |  | 0.991 | ± | 1.160 |  | |  | 0.405 | ± | 0.447* |
| **(±)14,15 EET** |  | 0.023 | ± | 0.008 |  | |  | 0.023 | ± | 0.012 |
| **5-HETE** |  | 0.179 | ± | 0.104 |  | |  | 0.131 | ± | 0.078 |
| **EPA** |  | 15.203 | ± | 6.683 |  | |  | 15.419 | ± | 9.066 |
| **DHA** |  | 107.484 | ± | 43.819 |  | |  | 134.094 | ± | 61.682 |
| **AA** |  | 362.446 | ± | 117.261 |  | |  | 469.597 | ± | 223.417 |
| **DHGLA** |  | 70.851 | ± | 25.607 |  | |  | 84.143 | ± | 59.805 |

**Supplemental Table 15. Eicosanoid profile observed in plasma from uncomplicated term pregnant patients recruited from the general OB/GYN clinic vs. total PE pregnant patients .** Raw, non-transformed eicosanoid levels observed in plasma from uncomplicated term pregnant patients recruited from the general OB/GYN clinic vs. total PE pregnant patients. Samples were taken prior to 24 weeks gestation and analyzed by UPLC ESI-M/S within two weeks of acquisition. Raw data were statistically compared using the Wilcoxon Sum Rank Test. The data presented are in Means ± SD in pmol lipid/100 µL plasma. Significance is represented as *p <0.05; **p<0.01; ***p<0.001; ****p<0.0001.

| **Analyte** |  | **Uncomplicated (n=14)** | | | |  |  | **Preeclamptic (n=26)** | | |
| --- | --- | --- | --- | --- | --- | --- | --- | --- | --- | --- |
|  |  | **pmol/100 µL Plasma** | | | |  |  | **pmol/100 µL Plasma** | | |
| **6-keto PGF1α** |  | 0.009 | ± | 0.006 |  | |  | 0.007 | ± | 0.002 |
| **TXB_2_** |  | 0.189 | ± | 0.185 |  | |  | 0.169 | ± | 0.186 |
| **PGE_2_** |  | 0.022 | ± | 0.021 |  | |  | 0.018 | ± | 0.013 |
| **PGD_2_** |  | 0.030 | ± | 0.020 |  | |  | 0.037 | ± | 0.034 |
| **Resolvin D1** |  | 0.133 | ± | 0.019 |  | |  | 0.131 | ± | 0.015 |
| **PGA_2_** |  | 0.235 | ± | 0.034 |  | |  | 0.232 | ± | 0.020 |
| **(±)14,15 DHET** |  | 0.122 | ± | 0.054 |  | |  | 0.092 | ± | 0.029 |
| **Maresin 2** |  | 0.009 | ± | 0.005 |  | |  | 0.010 | ± | 0.005 |
| **(±)11,12 DHET** |  | 0.130 | ± | 0.064 |  | |  | 0.088 | ± | 0.026* |
| **(±) 8,9 DHET** |  | 0.030 | ± | 0.018 |  | |  | 0.025 | ± | 0.007 |
| **20-HETE** |  | 13.005 | ± | 5.075 |  | |  | 9.394 | ± | 3.168* |
| **15-HETE** |  | 0.121 | ± | 0.059 |  | |  | 0.079 | ± | 0.032* |
| **12-HETE** |  | 0.328 | ± | 0.299 |  | |  | 0.405 | ± | 0.447 |
| **(±)14,15 EET** |  | 0.045 | ± | 0.063 |  | |  | 0.023 | ± | 0.012 |
| **5-HETE** |  | 0.167 | ± | 0.141 |  | |  | 0.131 | ± | 0.078 |
| **EPA** |  | 15.511 | ± | 7.092 |  | |  | 15.419 | ± | 9.066 |
| **DHA** |  | 137.007 | ± | 43.733 |  | |  | 134.094 | ± | 61.682 |
| **AA** |  | 599.688 | ± | 236.699 |  | |  | 469.597 | ± | 223.417 |
| **DHGLA** |  | 92.155 | ± | 42.884 |  | |  | 84.143 | ± | 59.805 |

**Supplemental Table 16. Eicosanoid profile observed in plasma from uncomplicated term pregnant patients recruited from the High-Risk OB/GYN clinic vs. total PE pregnant patients.** Raw, non-transformed eicosanoid levels observed in plasma from uncomplicated term pregnant patients recruited from the High-Risk OB/GYN clinic vs. total PE pregnant patients. Samples were taken prior to 24 weeks gestation and analyzed by UPLC ESI-M/S within two weeks of acquisition. Raw data were statistically compared using the Wilcoxon Sum Rank Test. The data presented are in Means ± SD in pmol lipid/100 µL plasma. Significance is represented as *p <0.05; **p<0.01; ***p<0.001; ****p<0.0001.

| **Analyte** | |  | | **Uncomplicated (n=16) pmol/50µL Plasma** | | |  | | **Preeclamptic (n=26)**  **pmol/50µL Plasma** | | |  |
| --- | --- | --- | --- | --- | --- | --- | --- | --- | --- | --- | --- | --- |
| **de18:1/14:0 Cer** |  | | 0.783 | | ± | 0.167 |  | 0.530 | | ± | 0.216**** | |
| **de18:1/16:0 Cer** |  | | 3.139 | | ± | 0.587 |  | 1.622 | | ± | 0.831**** | |
| **de18:1/18:0 Cer** |  | | 3.694 | | ± | 1.402 |  | 2.203 | | ± | 1.419**** | |
| **de18:1/20:0 Cer** |  | | 11.321 | | ± | 3.068 |  | 7.007 | | ± | 3.666**** | |
| **de18:1/22:0 Cer** |  | | 83.378 | | ± | 21.754 |  | 47.675 | | ± | 22.264**** | |
| **de18:1/24:1 Cer** |  | | 55.029 | | ± | 12.413 |  | 42.753 | | ± | 19.247* | |
| **de18:1/24:0 Cer** |  | | 227.483 | | ± | 51.306 |  | 121.972 | | ± | 52.833**** | |
| **de18:1/26:1 Cer** |  | | 1.660 | | ± | 0.447 |  | 2.530 | | ± | 0.789**** | |
| **de18:1/26:0 Cer** |  | | 2.867 | | ± | 0.984 |  | 1.624 | | ± | 0.987**** | |
| **de18:1/14:0 C1P** |  | | 0.970 | | ± | 0.429 |  | 0.652 | | ± | 0.269 | |
| **de18:1/16:0 C1P** |  | | 64.207 | | ± | 15.762 |  | 34.717 | | ± | 18.148**** | |
| **de18:1/22:0 C1P** |  | | 0.519 | | ± | 0.173 |  | 1.107 | | ± | 0.467**** | |
| **de18:1/24:1 C1P** |  | | 2.187 | | ± | 0.630 |  | 1.115 | | ± | 0.929**** | |
| **de18:1/24:0 C1P** |  | | 5.389 | | ± | 1.362 |  | 2.681 | | ± | 1.850**** | |
| **de18:1/14:0 SM** |  | | 122.319 | | ± | 22.224 |  | 61.833 | | ± | 34.830** | |
| **de18:1/16:0 SM** |  | | 100.466 | | ± | 23.182 |  | 49.406 | | ± | 32.552**** | |
| **de18:1/18:1 SM** |  | | 209.764 | | ± | 41.479 |  | 100.689 | | ± | 63.499** | |
| **de18:1/18:0 SM** |  | | 1.530 | | ± | 0.250 |  | 1.302 | | ± | 0.466**** | |
| **de18:1/20:0 SM** |  | | 1.944 | | ± | 0.599 |  | 0.957 | | ± | 0.786**** | |
| **de18:1/22:0 SM** |  | | 305.015 | | ± | 38.168 |  | 275.209 | | ± | 32.059**** | |
| **de18:1/24:1 SM** |  | | 255.556 | | ± | 21.679 |  | 215.547 | | ± | 72.705**** | |
| **de18:1/24:0 SM** |  | | 219.927 | | ± | 24.464 |  | 160.882 | | ± | 49.253 | |
| **de18:1/26:1 SM** |  | | 319.211 | | ± | 36.548 |  | 280.039 | | ± | 51.468**** | |
| **de18:1/26:0 SM** |  | | 406.359 | | ± | 28.459 |  | 360.336 | | ± | 44.657**** | |
| **de18:1/14:0 MonHex** |  | | 602.882 | | ± | 35.716 |  | 531.642 | | ± | 74.826**** | |
| **de18:1/16:0 MonHex** |  | | 500.769 | | ± | 33.774 |  | 500.887 | | ± | 110.969**** | |
| **de18:1/18:1 MonHex** |  | | 701.989 | | ± | 57.153 |  | 503.640 | | ± | 107.065**** | |
| **de18:1/18:0 MonHex** |  | | 34.118 | | ± | 9.981 |  | 23.247 | | ± | 7.976**** | |
| **de18:1/20:0 MonHex** |  | | 12.727 | | ± | 3.872 |  | 7.401 | | ± | 3.048**** | |
| **de18:1/22:0 MonHex** |  | | 0.206 | | ± | 0.078 |  | 0.176 | | ± | 0.052**** | |
| **de18:1/24:1 MonHex** |  | | 5.451 | | ± | 1.375 |  | 3.312 | | ± | 1.336**** | |
| **de18:1/24:0 MonHex** |  | | 2.193 | | ± | 0.475 |  | 1.086 | | ± | 0.521**** | |
| **de18:1/26:1 MonHex** |  | | 4.439 | | ± | 1.037 |  | 1.780 | | ± | 1.282* | |
| **de18:1/26:0 MonHex** |  | | 3.035 | | ± | 0.939 |  | 1.540 | | ± | 0.734**** | |
| **So** |  | | 1.866 | | ± | 1.551 |  | 1.229 | | ± | 0.746 | |
| **S1P** |  | | 51.878 | | ± | 13.377 |  | 41.853 | | ± | 12.541* | |
| **Sa1P** |  | | 8.711 | | ± | 2.583 |  | 6.780 | | ± | 2.274* | |

**Supplemental Table 17. Sphingolipid profile observed in plasma from uncomplicated term pregnant patients recruited from the general OB/GYN clinic vs. total PE pregnant patients.** Raw, non-transformed sphingolipid levels observed in plasma from uncomplicated term pregnant patients recruited from the general OB/GYN clinic vs. total PE pregnant patients. Samples were taken prior to 24 weeks gestation and analyzed by UPLC ESI-M/S within two weeks of acquisition. Raw data were statistically compared using the Wilcoxon Sum Rank Test. The data presented are in Means ± SD in pmol lipid/50 µL plasma. Significance is represented as *p <0.05; **p<0.01; ***p<0.001; ****p<0.0001.

| **Analyte** | |  | | **Uncomplicated (n=14) pmol/50µL Plasma** | | |  | | **Preeclamptic (n=26)**  **pmol/50µL Plasma** | | |  |
| --- | --- | --- | --- | --- | --- | --- | --- | --- | --- | --- | --- | --- |
| **de18:1/14:0 Cer** |  | | 0.628 | | ± | 0.248 |  | 0.530 | | ± | 0.216 | |
| **de18:1/16:0 Cer** |  | | 1.918 | | ± | 1.145 |  | 1.622 | | ± | 0.831 | |
| **de18:1/18:0 Cer** |  | | 2.861 | | ± | 2.246 |  | 2.203 | | ± | 1.419 | |
| **de18:1/20:0 Cer** |  | | 7.065 | | ± | 3.684 |  | 7.007 | | ± | 3.666 | |
| **de18:1/22:0 Cer** |  | | 41.108 | | ± | 19.626 |  | 47.675 | | ± | 22.264 | |
| **de18:1/24:1 Cer** |  | | 38.289 | | ± | 15.032 |  | 42.753 | | ± | 19.247 | |
| **de18:1/24:0 Cer** |  | | 100.092 | | ± | 33.663 |  | 121.972 | | ± | 52.833 | |
| **de18:1/26:1 Cer** |  | | 2.044 | | ± | 0.556 |  | 2.530 | | ± | 0.789 | |
| **de18:1/26:0 Cer** |  | | 1.598 | | ± | 0.676 |  | 1.624 | | ± | 0.987 | |
| **de18:1/14:0 C1P** |  | | 0.864 | | ± | 0.335 |  | 0.652 | | ± | 0.269 | |
| **de18:1/16:0 C1P** |  | | 45.808 | | ± | 20.149 |  | 34.717 | | ± | 18.148 | |
| **de18:1/22:0 C1P** |  | | 1.445 | | ± | 0.799 |  | 1.107 | | ± | 0.467 | |
| **de18:1/24:1 C1P** |  | | 1.521 | | ± | 0.962 |  | 1.115 | | ± | 0.929* | |
| **de18:1/24:0 C1P** |  | | 3.314 | | ± | 1.914 |  | 2.681 | | ± | 1.850 | |
| **de18:1/14:0 SM** |  | | 69.465 | | ± | 27.649 |  | 61.833 | | ± | 34.830 | |
| **de18:1/16:0 SM** |  | | 63.604 | | ± | 32.009 |  | 49.406 | | ± | 32.552 | |
| **de18:1/18:1 SM** |  | | 114.716 | | ± | 46.834 |  | 100.689 | | ± | 63.499 | |
| **de18:1/18:0 SM** |  | | 1.394 | | ± | 0.367 |  | 1.302 | | ± | 0.466 | |
| **de18:1/20:0 SM** |  | | 0.961 | | ± | 0.312 |  | 0.957 | | ± | 0.786 | |
| **de18:1/22:0 SM** |  | | 267.468 | | ± | 16.160 |  | 275.209 | | ± | 32.059* | |
| **de18:1/24:1 SM** |  | | 269.633 | | ± | 115.986 |  | 215.547 | | ± | 72.705* | |
| **de18:1/24:0 SM** |  | | 188.271 | | ± | 40.862 |  | 160.882 | | ± | 49.253 | |
| **de18:1/26:1 SM** |  | | 284.005 | | ± | 51.806 |  | 280.039 | | ± | 51.468* | |
| **de18:1/26:0 SM** |  | | 372.430 | | ± | 42.621 |  | 360.336 | | ± | 44.657 | |
| **de18:1/14:0 MonHex** |  | | 467.779 | | ± | 82.794 |  | 531.642 | | ± | 74.826* | |
| **de18:1/16:0 MonHex** |  | | 598.829 | | ± | 148.523 |  | 500.887 | | ± | 110.969* | |
| **de18:1/18:1 MonHex** |  | | 451.173 | | ± | 87.736 |  | 503.640 | | ± | 107.065 | |
| **de18:1/18:0 MonHex** |  | | 25.846 | | ± | 5.250 |  | 23.247 | | ± | 7.976 | |
| **de18:1/20:0 MonHex** |  | | 6.566 | | ± | 1.412 |  | 7.401 | | ± | 3.048 | |
| **de18:1/22:0 MonHex** |  | | 0.264 | | ± | 0.196 |  | 0.176 | | ± | 0.052 | |
| **de18:1/24:1 MonHex** |  | | 3.823 | | ± | 1.978 |  | 3.312 | | ± | 1.336* | |
| **de18:1/24:0 MonHex** |  | | 1.239 | | ± | 0.567 |  | 1.086 | | ± | 0.521 | |
| **de18:1/26:1 MonHex** |  | | 3.064 | | ± | 1.612 |  | 1.780 | | ± | 1.282 | |
| **de18:1/26:0 MonHex** |  | | 1.743 | | ± | 0.551 |  | 1.540 | | ± | 0.734 | |
| **So** |  | | 0.993 | | ± | 0.691 |  | 1.229 | | ± | 0.746 | |
| **S1P** |  | | 35.244 | | ± | 16.401 |  | 41.853 | | ± | 12.541* | |
| **Sa1P** |  | | 5.499 | | ± | 2.788 |  | 6.780 | | ± | 2.274 | |

**Supplemental Table 18. Sphingolipid profile observed in plasma from uncomplicated term pregnant patients recruited from the High-Risk OB/GYN clinic vs. total PE pregnant patients.** Raw, non-transformed sphingolipid levels observed in plasma from uncomplicated term pregnant patients recruited from the High-Risk OB/GYN clinic vs. total PE pregnant patients. Samples were taken prior to 24 weeks gestation and analyzed by UPLC ESI-M/S within two weeks of acquisition. Raw data were statistically compared using the Wilcoxon Sum Rank Test. The data presented are in Means ± SD in pmol lipid/50 µL plasma. Significance is represented as *p <0.05; **p<0.01; ***p<0.001; ****p<0.0001.

| **Analyte** |  | **General Clinic (n=16)** | | | |  |  | **High Risk Clinic (n=14)** | | |
| --- | --- | --- | --- | --- | --- | --- | --- | --- | --- | --- |
|  |  | **pmol/100 µL Plasma** | | | |  |  | **pmol/100 µL Plasma** | | |
| **6-keto PGF1α** |  | 0.007 | ± | 0.002 |  | |  | 0.009 | ± | 0.006 |
| **TXB_2_** |  | 0.268 | ± | 0.239 |  | |  | 0.189 | ± | 0.185 |
| **PGE_2_** |  | 0.032 | ± | 0.027 |  | |  | 0.022 | ± | 0.021 |
| **PGD_2_** |  | 0.029 | ± | 0.027 |  | |  | 0.030 | ± | 0.020 |
| **Resolvin D1** |  | 0.120 | ± | 0.003 |  | |  | 0.133 | ± | 0.019** |
| **PGA_2_** |  | 0.230 | ± | 0.009 |  | |  | 0.235 | ± | 0.034 |
| **(±)14,15 DHET** |  | 0.098 | ± | 0.022 |  | |  | 0.122 | ± | 0.054 |
| **Maresin 2** |  | 0.009 | ± | 0.003 |  | |  | 0.009 | ± | 0.005 |
| **(±)11,12 DHET** |  | 0.101 | ± | 0.027 |  | |  | 0.130 | ± | 0.064 |
| **(±) 8,9 DHET** |  | 0.030 | ± | 0.010 |  | |  | 0.030 | ± | 0.018 |
| **20-HETE** |  | 6.238 | ± | 0.971 |  | |  | 13.005 | ± | 5.075**** |
| **15-HETE** |  | 0.122 | ± | 0.101 |  | |  | 0.121 | ± | 0.059 |
| **12-HETE** |  | 0.991 | ± | 1.160 |  | |  | 0.328 | ± | 0.299* |
| **(±)14,15 EET** |  | 0.023 | ± | 0.008 |  | |  | 0.045 | ± | 0.063 |
| **5-HETE** |  | 0.179 | ± | 0.104 |  | |  | 0.167 | ± | 0.141 |
| **EPA** |  | 15.203 | ± | 6.683 |  | |  | 15.511 | ± | 7.092 |
| **DHA** |  | 107.484 | ± | 43.819 |  | |  | 137.007 | ± | 43.733 |
| **AA** |  | 362.446 | ± | 117.261 |  | |  | 599.688 | ± | 236.699** |
| **DHGLA** |  | 70.851 | ± | 25.607 |  | |  | 92.155 | ± | 42.884 |

**Supplemental Table 19. Eicosanoid profile observed in the plasma from uncomplicated term pregnant patients recruited from General OB/GYN clinic vs. uncomplicated term pregnant patients referred to High-Risk OB/GYN clinic.** Raw, non-transformed eicosanoid levels in the plasma from uncomplicated term pregnant patients recruited from General OB/GYN clinic vs. uncomplicated term pregnant patients referred to High-Risk OB/GYN clinic. Samples were taken prior to 24 weeks gestation and analyzed by UPLC ESI-M/S within two weeks of acquisition. Raw data were statistically compared using the Wilcoxon Sum Rank Test. The data presented are in Means ± SD in pmol lipid/100 µL plasma. Significance is represented as *p <0.05; **p<0.01; ***p<0.001; ****p<0.0001.

| **Analyte** | |  | | **General Clinic (n=16) pmol/50µL Plasma** | | |  | | **High Risk Clinic (n=14)**  **pmol/50µL Plasma** | | |  |
| --- | --- | --- | --- | --- | --- | --- | --- | --- | --- | --- | --- | --- |
| **de18:1/14:0 Cer** |  | | 0.783 | | ± | 0.167 |  | 0.628 | | ± | 0.248 | |
| **de18:1/16:0 Cer** |  | | 3.139 | | ± | 0.587 |  | 1.918 | | ± | 1.145**** | |
| **de18:1/18:0 Cer** |  | | 3.694 | | ± | 1.402 |  | 2.861 | | ± | 2.246* | |
| **de18:1/20:0 Cer** |  | | 11.321 | | ± | 3.068 |  | 7.065 | | ± | 3.684** | |
| **de18:1/22:0 Cer** |  | | 83.378 | | ± | 21.754 |  | 41.108 | | ± | 19.626**** | |
| **de18:1/24:1 Cer** |  | | 55.029 | | ± | 12.413 |  | 38.289 | | ± | 15.032** | |
| **de18:1/24:0 Cer** |  | | 227.483 | | ± | 51.306 |  | 100.092 | | ± | 33.663**** | |
| **de18:1/26:1 Cer** |  | | 1.660 | | ± | 0.447 |  | 2.044 | | ± | 0.556 | |
| **de18:1/26:0 Cer** |  | | 2.867 | | ± | 0.984 |  | 1.598 | | ± | 0.676**** | |
| **de18:1/14:0 C1P** |  | | 0.970 | | ± | 0.429 |  | 0.864 | | ± | 0.335 | |
| **de18:1/16:0 C1P** |  | | 64.207 | | ± | 15.762 |  | 45.808 | | ± | 20.149* | |
| **de18:1/22:0 C1P** |  | | 0.519 | | ± | 0.173 |  | 1.445 | | ± | 0.799**** | |
| **de18:1/24:1 C1P** |  | | 2.187 | | ± | 0.630 |  | 1.521 | | ± | 0.962** | |
| **de18:1/24:0 C1P** |  | | 5.389 | | ± | 1.362 |  | 3.314 | | ± | 1.914**** | |
| **de18:1/14:0 SM** |  | | 122.319 | | ± | 22.224 |  | 69.465 | | ± | 27.649**** | |
| **de18:1/16:0 SM** |  | | 100.466 | | ± | 23.182 |  | 63.604 | | ± | 32.009 | |
| **de18:1/18:1 SM** |  | | 209.764 | | ± | 41.479 |  | 114.716 | | ± | 46.834* | |
| **de18:1/18:0 SM** |  | | 1.530 | | ± | 0.250 |  | 1.394 | | ± | 0.367 | |
| **de18:1/20:0 SM** |  | | 1.944 | | ± | 0.599 |  | 0.961 | | ± | 0.312* | |
| **de18:1/22:0 SM** |  | | 305.015 | | ± | 38.168 |  | 267.468 | | ± | 16.160**** | |
| **de18:1/24:1 SM** |  | | 255.556 | | ± | 21.679 |  | 269.633 | | ± | 115.986 | |
| **de18:1/24:0 SM** |  | | 219.927 | | ± | 24.464 |  | 188.271 | | ± | 40.862**** | |
| **de18:1/26:1 SM** |  | | 319.211 | | ± | 36.548 |  | 284.005 | | ± | 51.806** | |
| **de18:1/26:0 SM** |  | | 406.359 | | ± | 28.459 |  | 372.430 | | ± | 42.621**** | |
| **de18:1/14:0 MonHex** |  | | 602.882 | | ± | 35.716 |  | 467.779 | | ± | 82.794 | |
| **de18:1/16:0 MonHex** |  | | 500.769 | | ± | 33.774 |  | 598.829 | | ± | 148.523** | |
| **de18:1/18:1 MonHex** |  | | 701.989 | | ± | 57.153 |  | 451.173 | | ± | 87.736* | |
| **de18:1/18:0 MonHex** |  | | 34.118 | | ± | 9.981 |  | 25.846 | | ± | 5.250**** | |
| **de18:1/20:0 MonHex** |  | | 12.727 | | ± | 3.872 |  | 6.566 | | ± | 1.412** | |
| **de18:1/22:0 MonHex** |  | | 0.206 | | ± | 0.078 |  | 0.264 | | ± | 0.196**** | |
| **de18:1/24:1 MonHex** |  | | 5.451 | | ± | 1.375 |  | 3.823 | | ± | 1.978** | |
| **de18:1/24:0 MonHex** |  | | 2.193 | | ± | 0.475 |  | 1.239 | | ± | 0.567**** | |
| **de18:1/26:1 MonHex** |  | | 4.439 | | ± | 1.037 |  | 3.064 | | ± | 1.612 | |
| **de18:1/26:0 MonHex** |  | | 3.035 | | ± | 0.939 |  | 1.743 | | ± | 0.551**** | |
| **So** |  | | 1.866 | | ± | 1.551 |  | 0.993 | | ± | 0.691 | |
| **S1P** |  | | 51.878 | | ± | 13.377 |  | 35.244 | | ± | 16.401* | |
| **Sa1P** |  | | 8.711 | | ± | 2.583 |  | 5.499 | | ± | 2.788* | |

**Supplemental Table 20. Sphingolipid profile observed in the plasma from uncomplicated term pregnant patients recruited from General OB/GYN clinic vs. uncomplicated term pregnant patients referred to High-Risk OB/GYN clinic.** Raw, non-transformed sphingolipid levels in the plasma from uncomplicated term pregnant patients recruited from General OB/GYN clinic vs. uncomplicated term pregnant patients referred to High-Risk OB/GYN clinic. Samples were taken prior to 24 weeks gestation and analyzed by UPLC ESI-M/S within two weeks of acquisition. Raw data were statistically compared using the Wilcoxon Sum Rank Test. The data presented are in Means ± SD in pmol lipid/50 µL plasma. Significance is represented as *p <0.05; **p<0.01; ***p<0.001; ****p<0.0001.


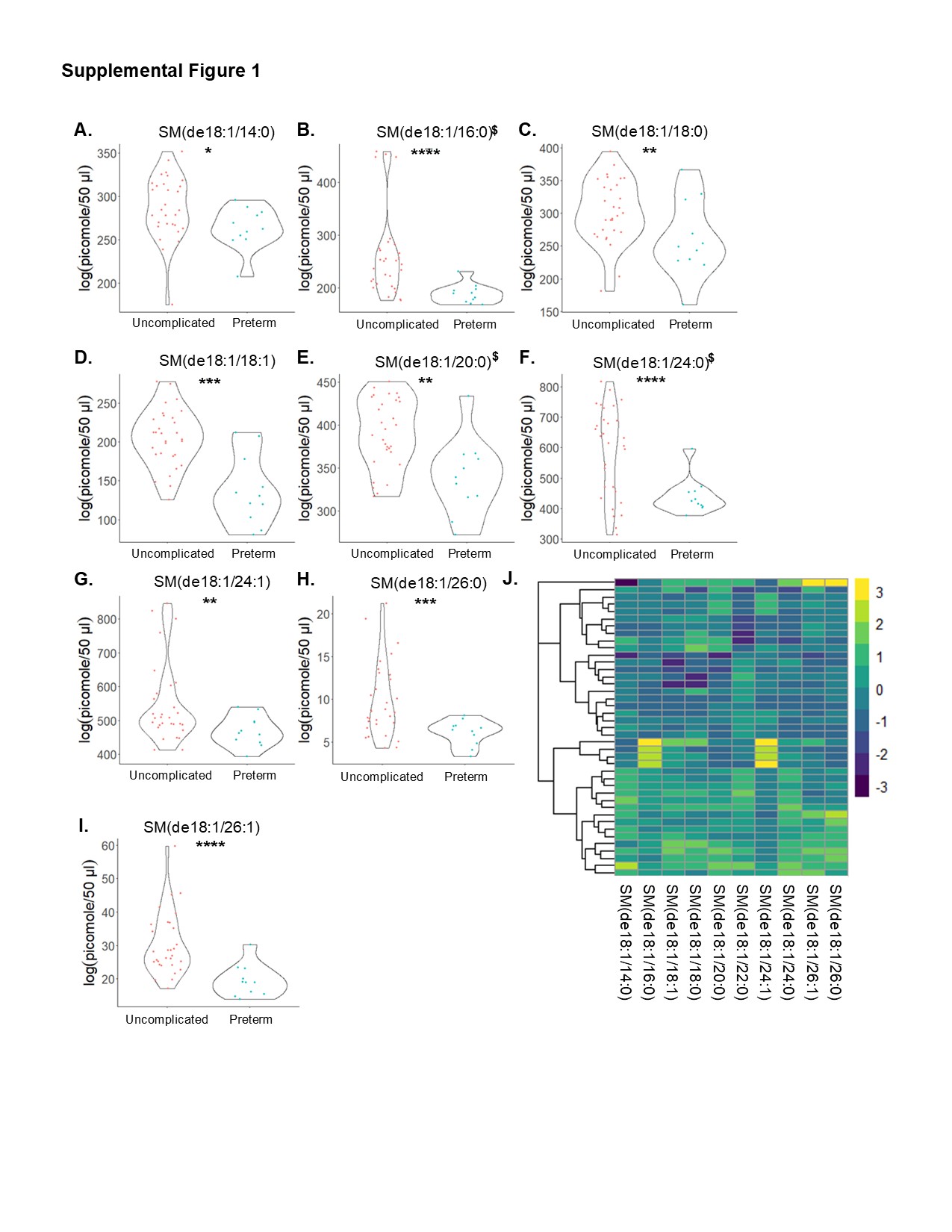
**Supplemental Figure 1. Sphingomyelin levels show significant differences in the plasma from uncomplicated term pregnant patients vs. PE pregnancies with a preterm birth. (A-I)** Sphingomyelin species that occurred at significantly different levels when comparing plasma from uncomplicated term pregnant patients vs. PE pregnancies with a preterm birth using UPLC ESI-MS/MS as the detection method. Samples were analyzed by UPLC ESI-MS/MS within two weeks of acquisition. **(J)** Heatmap of all sphingomyelin species that were detected via UPLC ESI-MS/MS in plasma (fold change is depicted). Samples were compared using unpaired students t-test with Welch’s correction. Data shown are means + SD depicted as violin plots, *P< 0.05, **P< 0.01, ***P< 0.001, ****P< 0.0001. The log transformed data failing the Shapiro-Wilk Test are designated with a $.


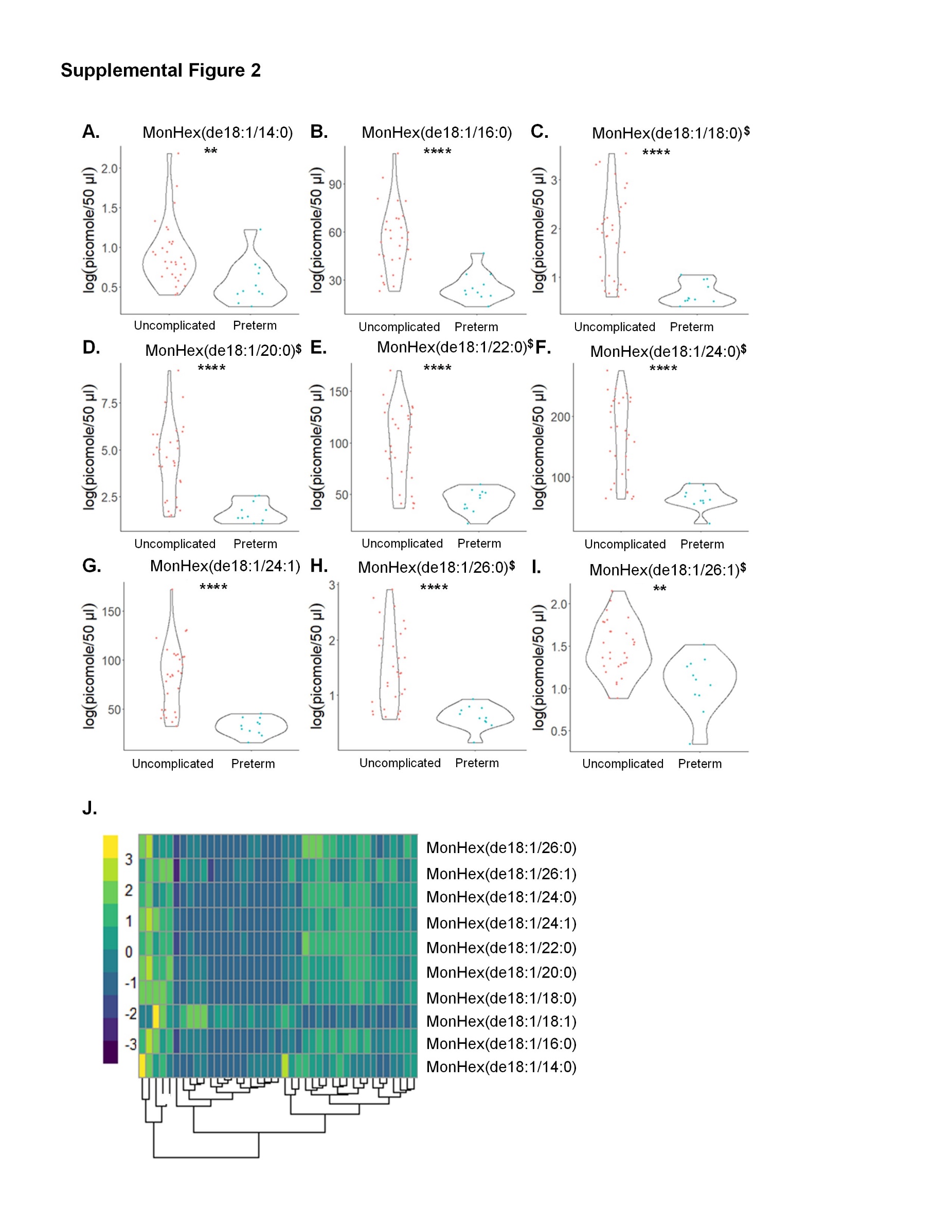


**Supplemental Figure 2. MonHex species show significant differences in the plasma from uncomplicated term pregnant patients vs. PE pregnancies with a preterm birth. (A-I**) MonHex species that occurred at significantly different levels when comparing plasma from uncomplicated term pregnant patients vs. PE pregnancies with a preterm birth using UPLC ESI-MS/MS as the detection method. Samples were analyzed by UPLC ESI-MS/MS within two weeks of acquisition. **(J)** Heatmap of all MonHex species that were detected via UPLC ESI-MS/MS in plasma (fold change is depicted). Samples were compared using unpaired students t-test with Welch’s correction. Data shown are means + SD depicted as violin plots, *P< 0.05, **P< 0.01, ***P< 0.001, ****P< 0.0001. The log transformed data failing the Shapiro-Wilk Test are designated with a $.


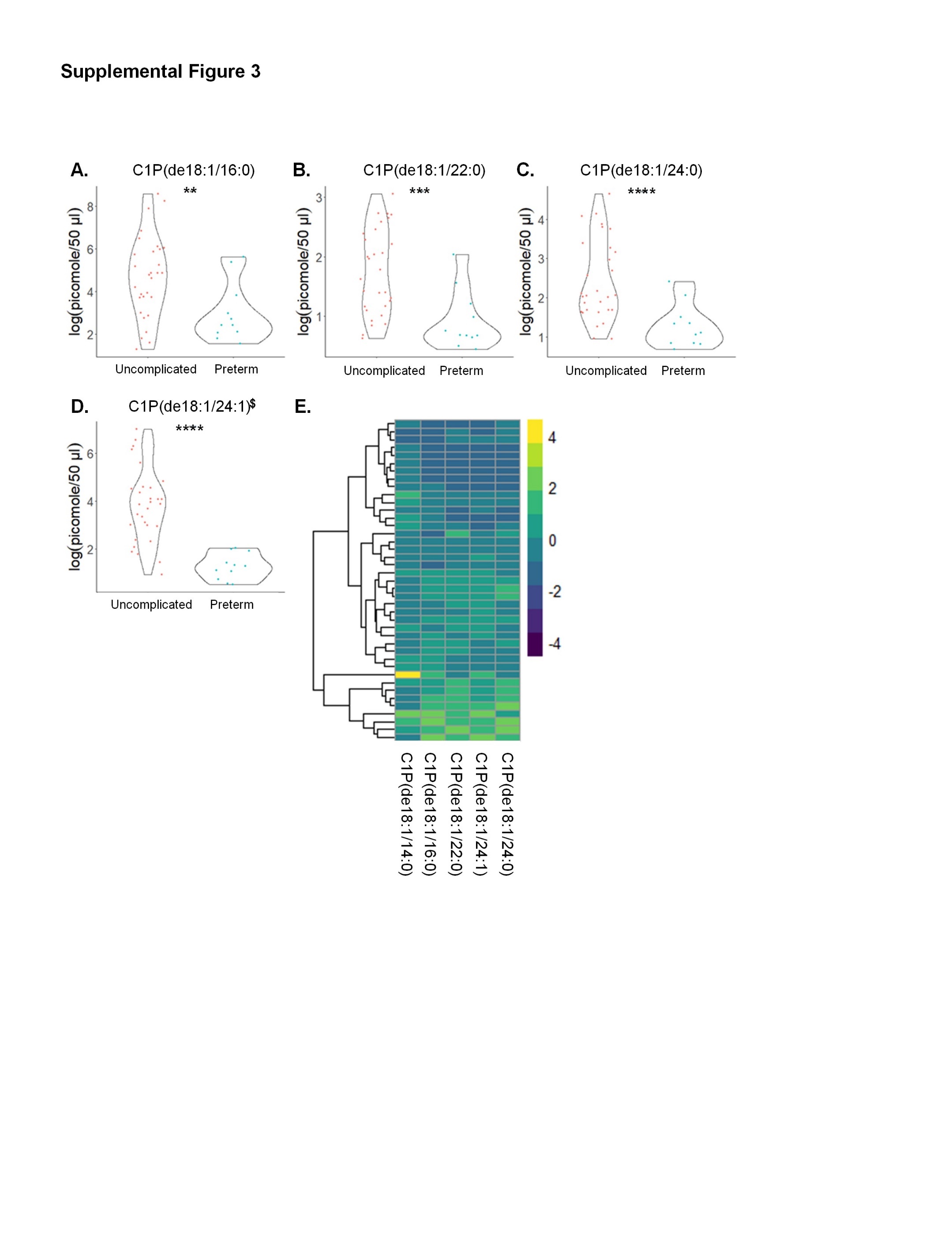


**Supplemental Figure 3. C1P levels show significant differences in the plasma from uncomplicated term pregnant patients vs. PE pregnancies with a preterm birth. (A-D**) C1P species that occurred at significantly different levels when comparing plasma from uncomplicated term pregnant patients vs. PE pregnancies with a preterm birth using UPLC ESI-MS/MS as the detection method. Samples were analyzed by UPLC ESI-MS/MS within two weeks of acquisition. **(E)** Heatmap of all C1P species that were detected via UPLC ESI-MS/MS in plasma (fold change is depicted). Samples were compared using unpaired students t-test with Welch’s correction. Data shown are means + SD depicted as violin plots, *P< 0.05, **P< 0.01, ***P< 0.001, ****P< 0.0001. The log transformed data failing the Shapiro-Wilk Test are designated with a $.


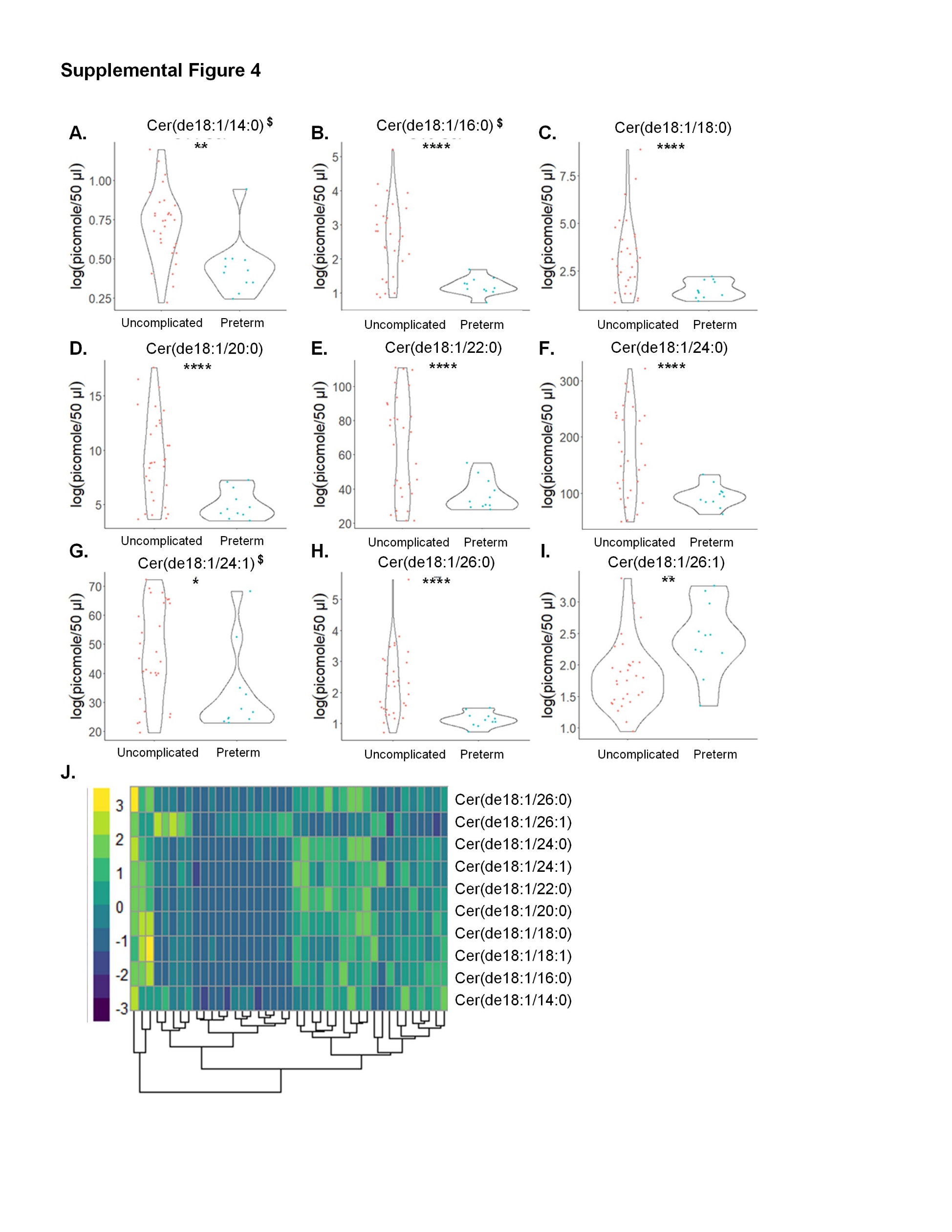


**Supplemental Figure 4. Ceramide species show significant differences in the plasma from uncomplicated term pregnant patients vs. PE pregnancies with a preterm birth. (A-I**) Ceramide species that occurred at significantly different levels when comparing plasma from uncomplicated term pregnant patients vs. PE pregnancies with a preterm birth using UPLC ESI-MS/MS as the detection method. Samples were analyzed by UPLC ESI-MS/MS within two weeks of acquisition. **(J)** Heatmap of all Ceramide species that were detected via UPLC ESI-MS/MS in plasma (fold change is depicted). Samples were compared using unpaired students t-test with Welch’s correction. Data shown are means + SD depicted as violin plots, *P< 0.05, **P< 0.01, ***P< 0.001, ****P< 0.0001. The log transformed data failing the Shapiro-Wilk Test are designated with a $.


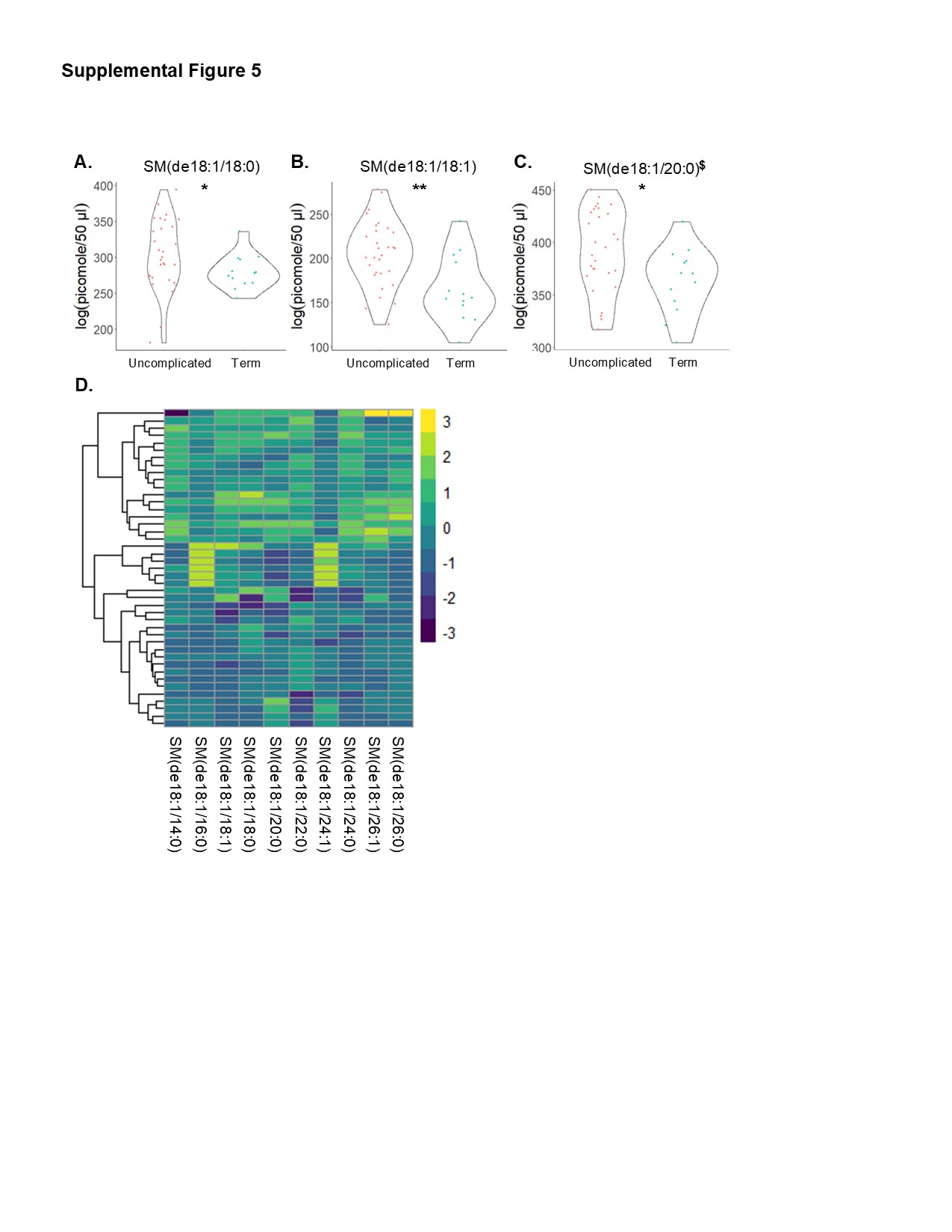


**Supplemental Figure 5. Sphingomyelin levels show significant differences in the plasma from uncomplicated term pregnant patients vs. term PE pregnancies. (A-C)** Sphingomyelin species that occurred at significantly different levels when comparing plasma from patients with normal pregnancies to patients with term PE pregnancies using UPLC ESI-MS/MS as the detection method. Samples were analyzed by UPLC ESI-MS/MS within two weeks of acquisition. **(D)** Heatmap of all sphingomyelin species that were detected via UPLC ESI-MS/MS in plasma (fold change is depicted). Samples were compared using unpaired students t-test with Welch’s correction. Data shown are means + SD depicted as violin plots, *P< 0.05, **P< 0.01, ***P< 0.001, ****P< 0.0001. The log transformed data failing the Shapiro-Wilk Test are designated with a $.


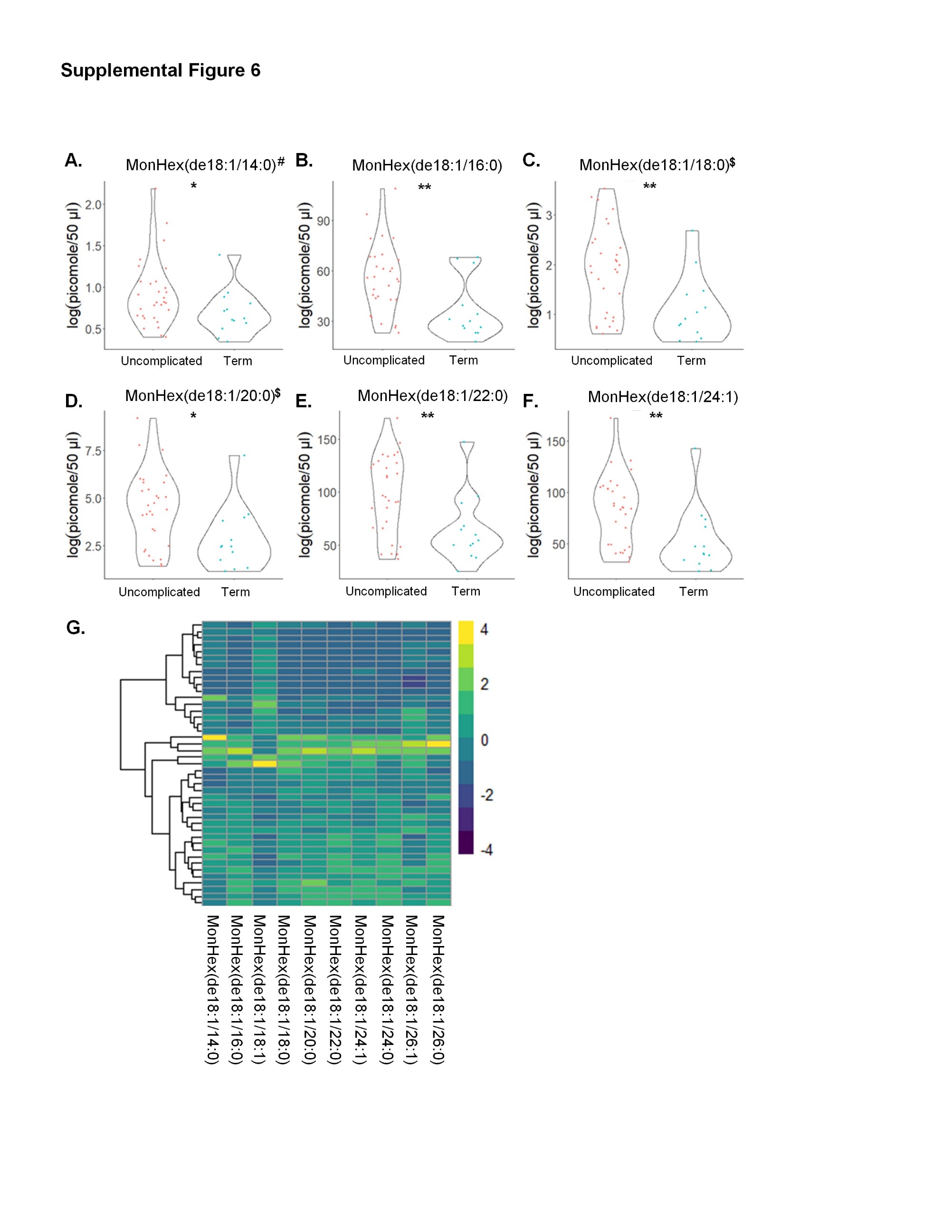


**Supplemental Figure 6. MonHex species show significant differences in the plasma from uncomplicated term pregnant patients vs. term PE pregnancies. (A-F**) MonHex species that occurred at significantly different levels when comparing plasma from patients with normal pregnancies to patients with term PE pregnancies using UPLC ESI-MS/MS as the detection method. Samples were analyzed by UPLC ESI-MS/MS within two weeks of acquisition. **(G)** Heatmap of all MonHex species that were detected via UPLC ESI-MS/MS in plasma (fold change is depicted). Samples were compared using unpaired students t-test with Welch’s correction. Data shown are means + SD depicted as violin plots, *P< 0.05, **P< 0.01, ***P< 0.001, ****P< 0.0001. The log transformed data failing the Shapiro-Wilk Test are designated with a $. Non-transformed data were also analyzed by the Wilcoxon Sum Rank Test. Bioactive lipid mediators not found to be significantly different by the Wilcoxon Sum Rank Test are designated with a #.


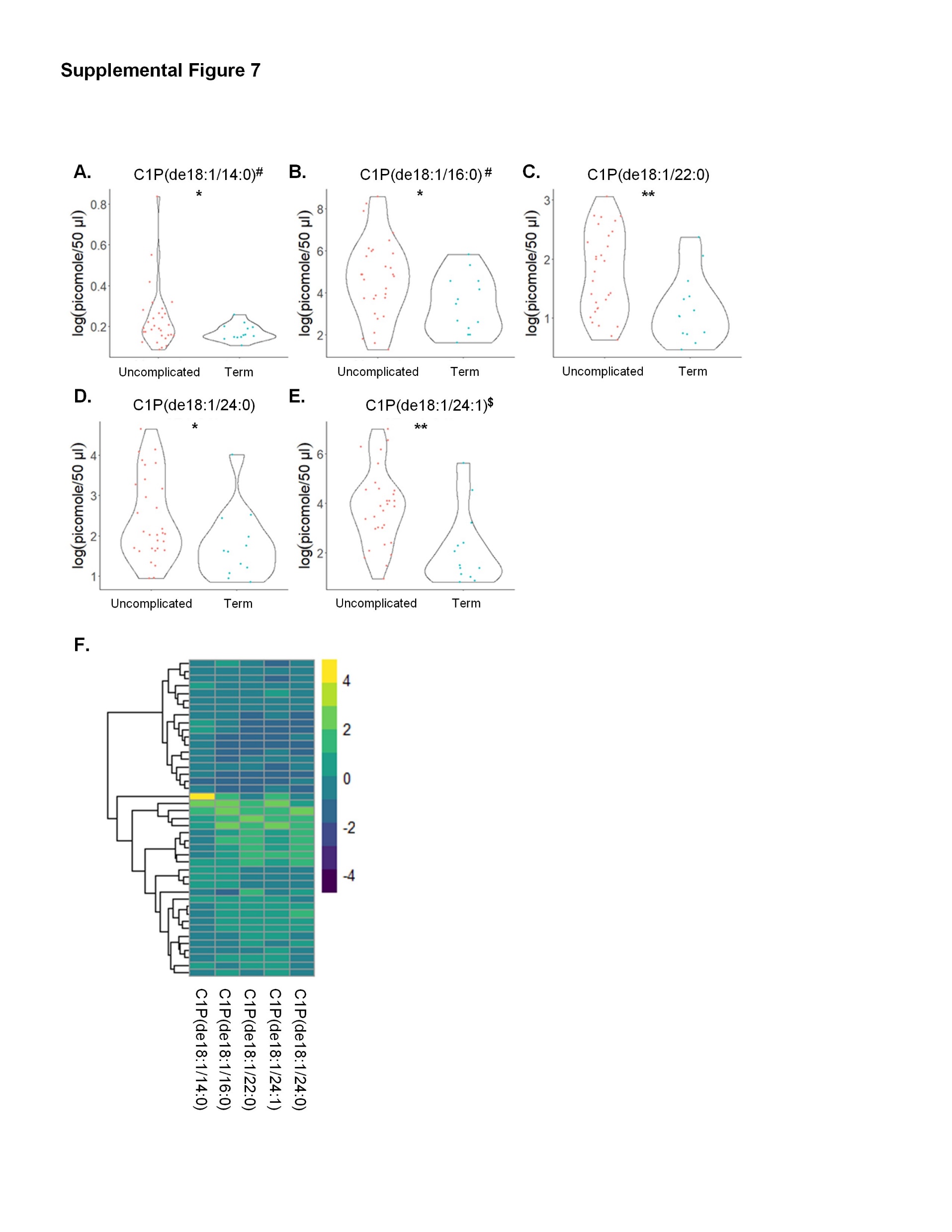


**Supplemental Figure 7. C1P levels show significant differences in the plasma from uncomplicated term pregnant patients vs. term PE pregnancies. (A-E**) C1P species that occurred at significantly different levels when comparing plasma from patients with normal pregnancies to patients with term PE pregnancies using UPLC ESI-MS/MS as the detection method. Samples were analyzed by UPLC ESI-MS/MS within two weeks of acquisition. **(F)** Heatmap of all C1P species that were detected via UPLC ESI-MS/MS in plasma (fold change is depicted). Samples were compared using unpaired students t-test with Welch’s correction. Data shown are means + SD depicted as violin plots, *P< 0.05, **P< 0.01, ***P< 0.001, ****P< 0.0001. The log transformed data failing the Shapiro-Wilk Test are designated with a $. Non-transformed data were also analyzed by the Wilcoxon Sum Rank Test. Bioactive lipid mediators not found to be significantly different by the Wilcoxon Sum Rank Test are designated with a #.


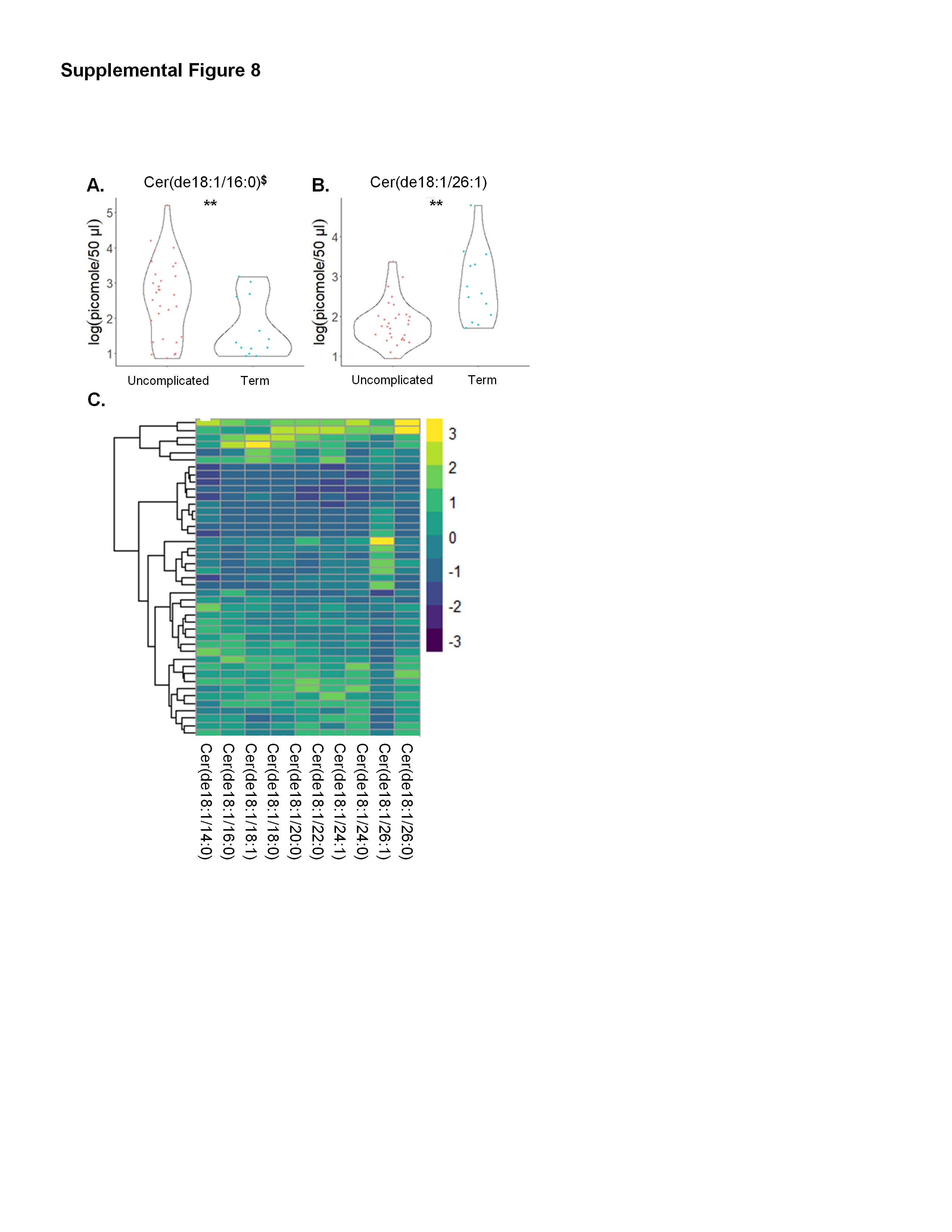


**Supplemental Figure 8. Ceramide species show significant differences in the plasma from uncomplicated term pregnant patients vs. term PE pregnancies. (A,B**) Ceramide species that occurred at significantly different levels when comparing plasma from patients with normal pregnancies to patients with term PE pregnancies using UPLC ESI-MS/MS as the detection method. Samples were analyzed by UPLC ESI-MS/MS within two weeks of acquisition. **(C)** Heatmap of all Ceramide species that were detected via UPLC ESI-MS/MS in plasma (fold change is depicted). Samples were compared using unpaired students t-test with Welch’s correction. Data shown are means + SD depicted as violin plots, *P< 0.05, **P< 0.01, ***P< 0.001, ****P< 0.0001. The log transformed data failing the Shapiro-Wilk Test are designated with a $.


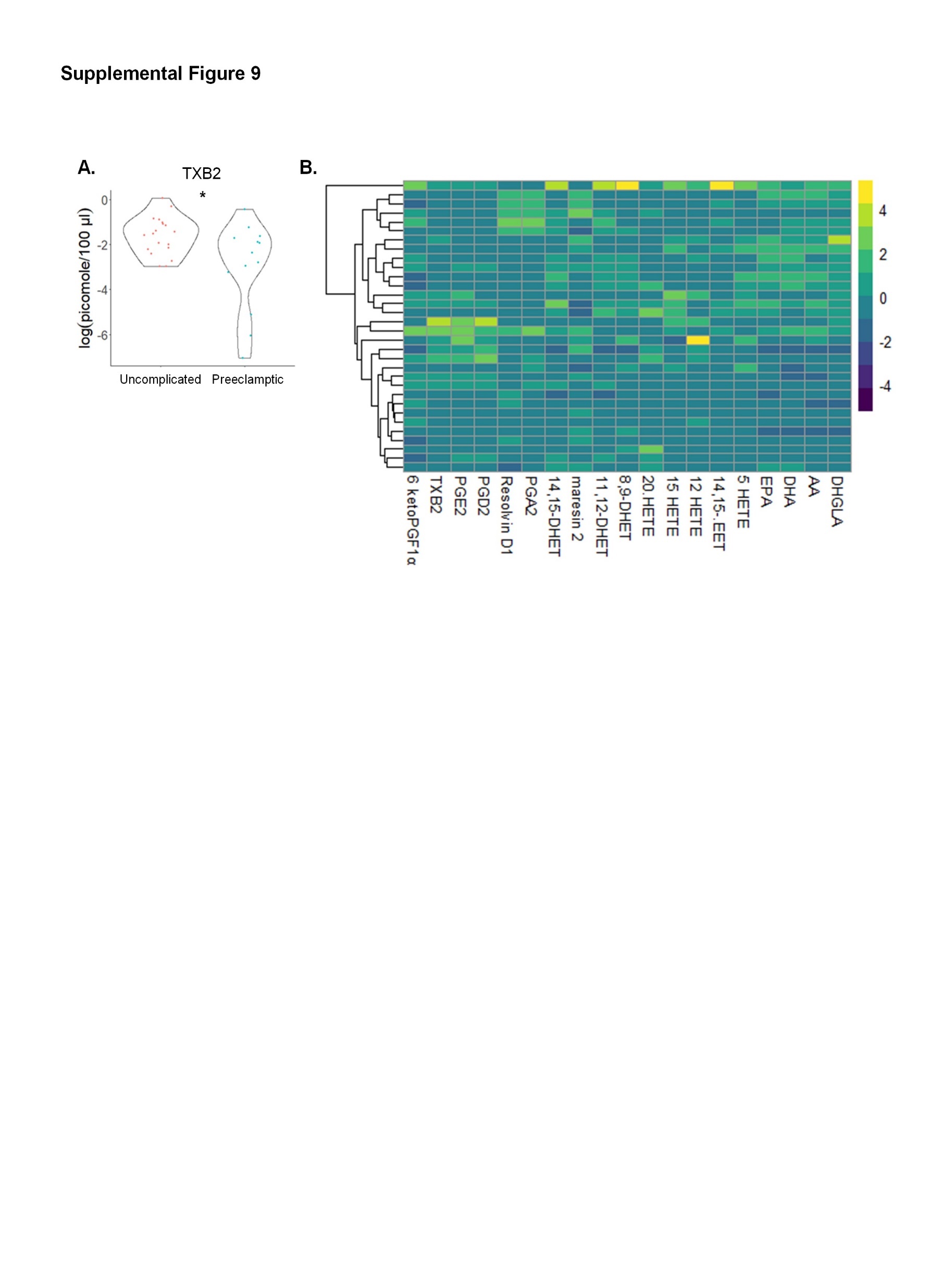


**Supplemental Figure 9. Distinct eicosanoids show significant differences in the plasma from uncomplicated term pregnant patients of African descent vs. PE patients of African descent. (A**) Eicosanoid species that occurred at significantly different levels when comparing plasma from uncomplicated term pregnant patients of African descent to patients of African descent with PE using UPLC ESI-MS/MS as the detection method. Samples were analyzed by UPLC ESI-MS/MS within two weeks of acquisition. **(B)** Heatmap of all Eicosanoid species that were detected via UPLC ESI-MS/MS in plasma (fold change is depicted). Samples were compared using unpaired students t-test with Welch’s correction. Data shown are means + SD depicted as violin plots, *P< 0.05, **P< 0.01, ***P< 0.001, ****P< 0.0001.


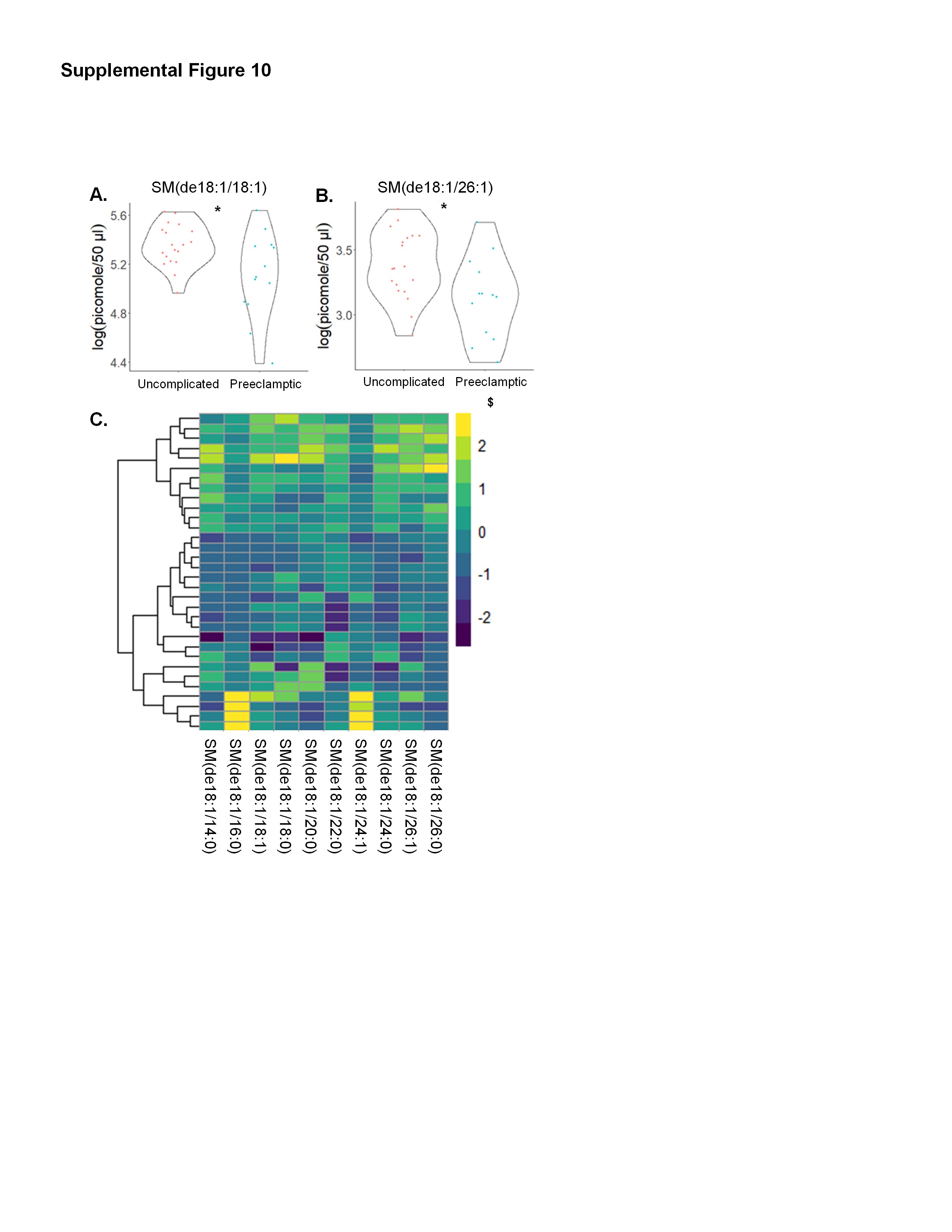


**Supplemental Figure 10. Sphingomyelin levels show significant differences in the plasma from uncomplicated term pregnant patients of African descent vs. PE patients of African descent. (A,B)** Sphingomyelin species that occurred at significantly different levels when comparing plasma from uncomplicated term pregnant patients of African descent to patients of African descent with PE using UPLC ESI-MS/MS as the detection method. Samples were analyzed by UPLC ESI-MS/MS within two weeks of acquisition. **(C)** Heatmap of all sphingomyelin species that were detected via UPLC ESI-MS/MS in plasma (fold change is depicted). Samples were compared using unpaired students t-test with Welch’s correction. Data shown are means + SD depicted as violin plots, *P< 0.05, **P< 0.01, ***P< 0.001, ****P< 0.0001.


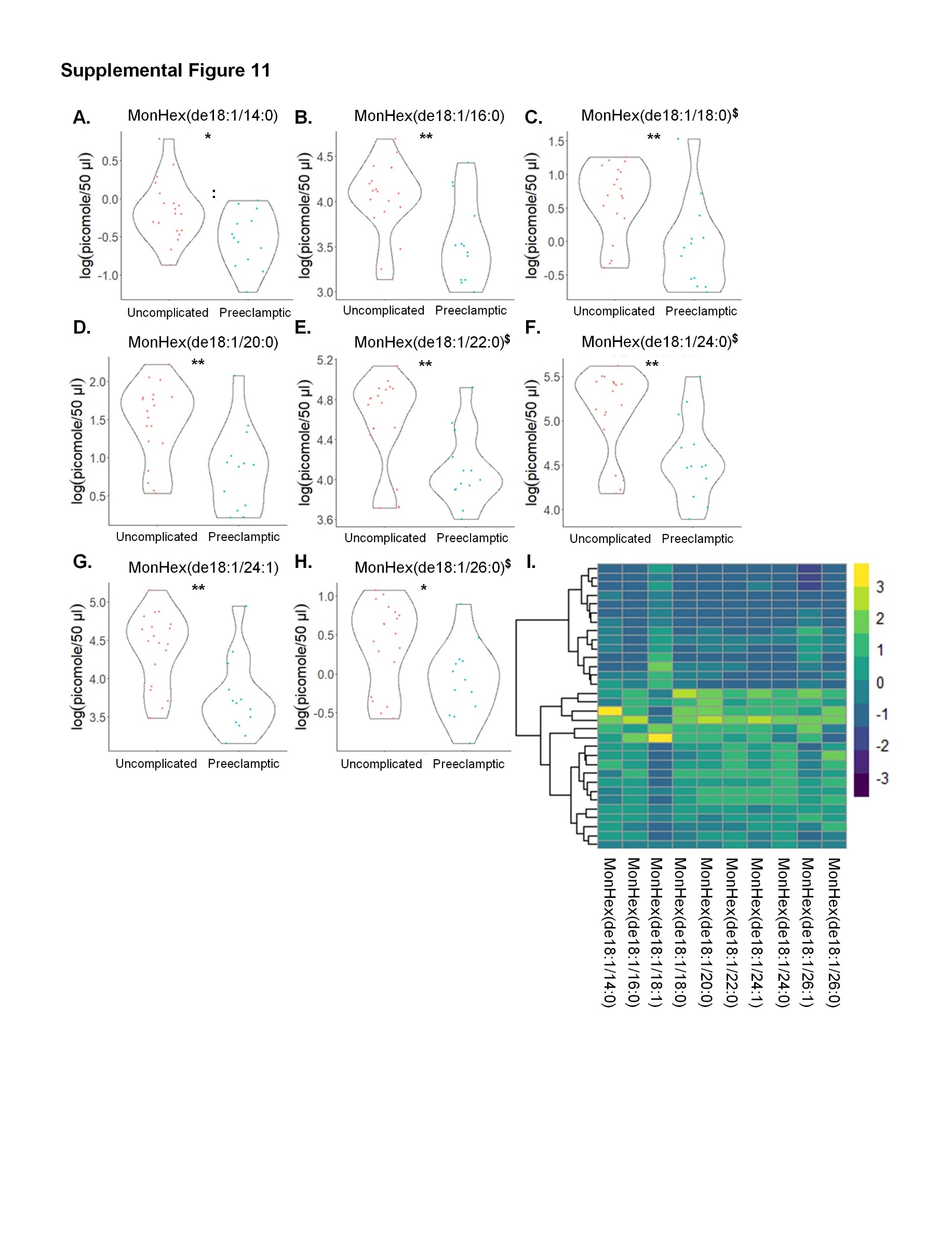


**Supplemental Figure 11. MonHex species show significant differences in the plasma from uncomplicated term pregnant patients of African descent vs. PE patients of African descent. (A-H**) MonHex species that occurred at significantly different levels when comparing plasma from uncomplicated term pregnant patients of African descent to patients of African descent with PE using UPLC ESI-MS/MS as the detection method. Samples were analyzed by UPLC ESI-MS/MS within two weeks of acquisition. **(I)** Heatmap of all MonHex species that were detected via UPLC ESI-MS/MS in plasma (fold change is depicted). Samples were compared using unpaired students t-test with Welch’s correction. Data shown are means + SD depicted as violin plots, *P< 0.05, **P< 0.01, ***P< 0.001, ****P< 0.0001. The log transformed data failing the Shapiro-Wilk Test are designated with a $.


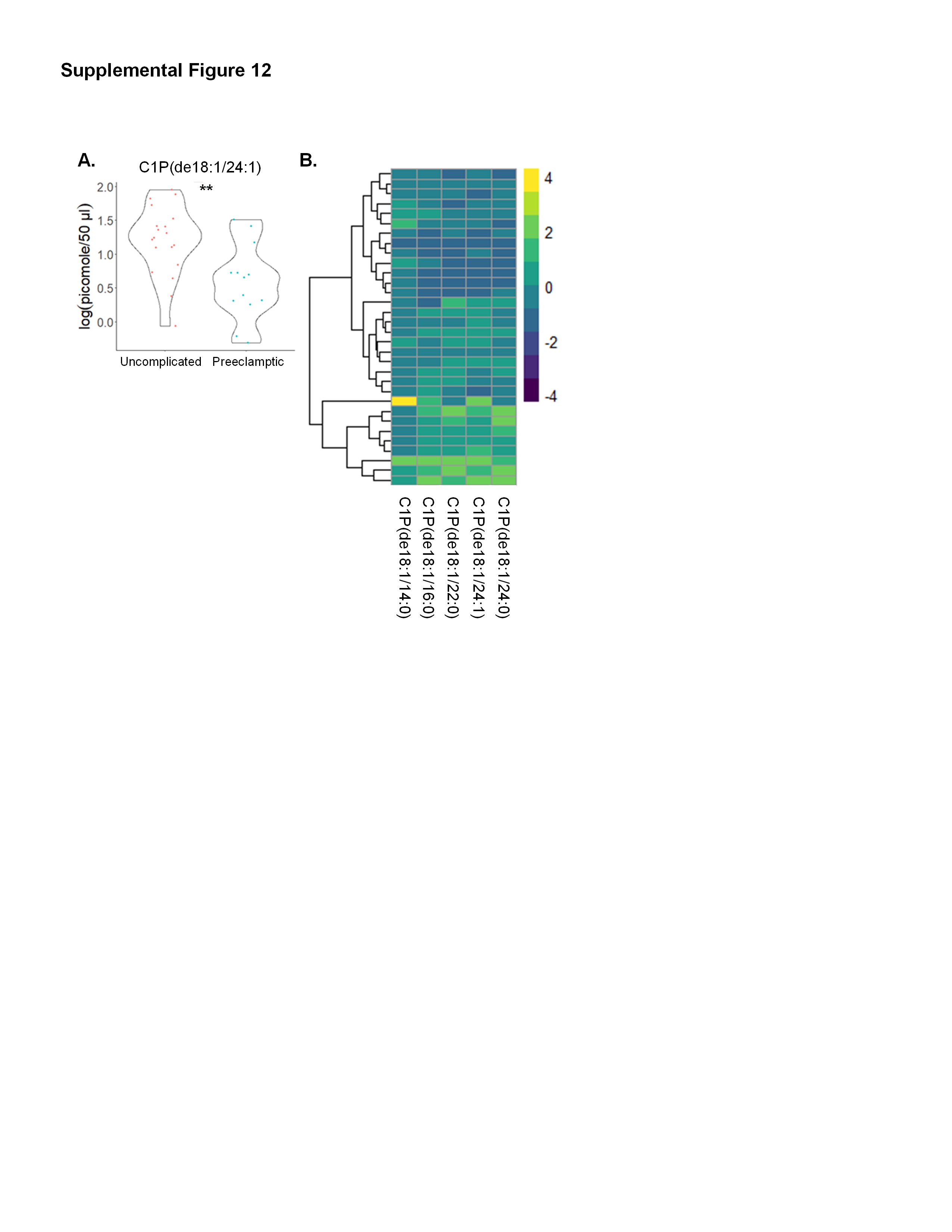


**Supplemental Figure 12. C1P levels show significant differences in the plasma from uncomplicated term pregnant patients of African descent vs. PE patients of African descent. (A**) C1P species that occurred at significantly different levels when comparing plasma from uncomplicated term pregnant patients of African descent to patients of African descent with PE using UPLC ESI-MS/MS as the detection method. Samples were analyzed by UPLC ESI-MS/MS within two weeks of acquisition. **(B)** Heatmap of all C1P species that were detected via UPLC ESI-MS/MS in plasma (fold change is depicted). Samples were compared using unpaired students t-test with Welch’s correction. Data shown are means + SD depicted as violin plots, *P< 0.05, **P< 0.01, ***P< 0.001, ****P< 0.0001.


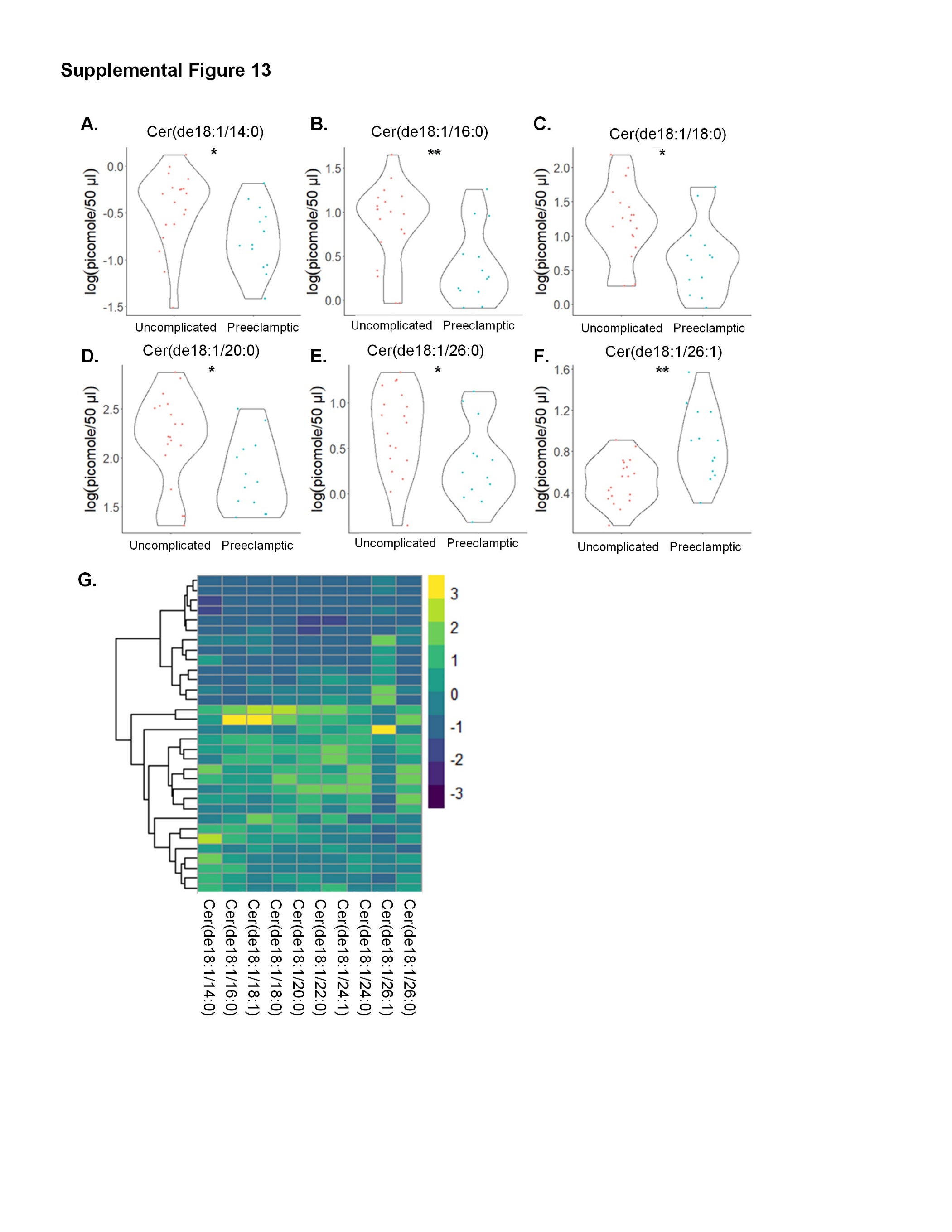


**Supplemental Figure 13. Ceramide species show significant differences in the plasma from uncomplicated term pregnant patients of African descent vs. PE patients of African descent. (A-F**) that occurred at significantly different levels when comparing plasma from uncomplicated term pregnant patients of African descent to patients of African descent with PE using UPLC ESI-MS/MS as the detection method. Samples were analyzed by UPLC ESI-MS/MS within two weeks of acquisition. **(G)** Heatmap of all Ceramide species that were detected via UPLC ESI-MS/MS in plasma (fold change is depicted). Samples were compared using unpaired students t-test with Welch’s correction. Data shown are means + SD depicted as violin plots, *P< 0.05, **P< 0.01, ***P< 0.001, ****P< 0.0001.


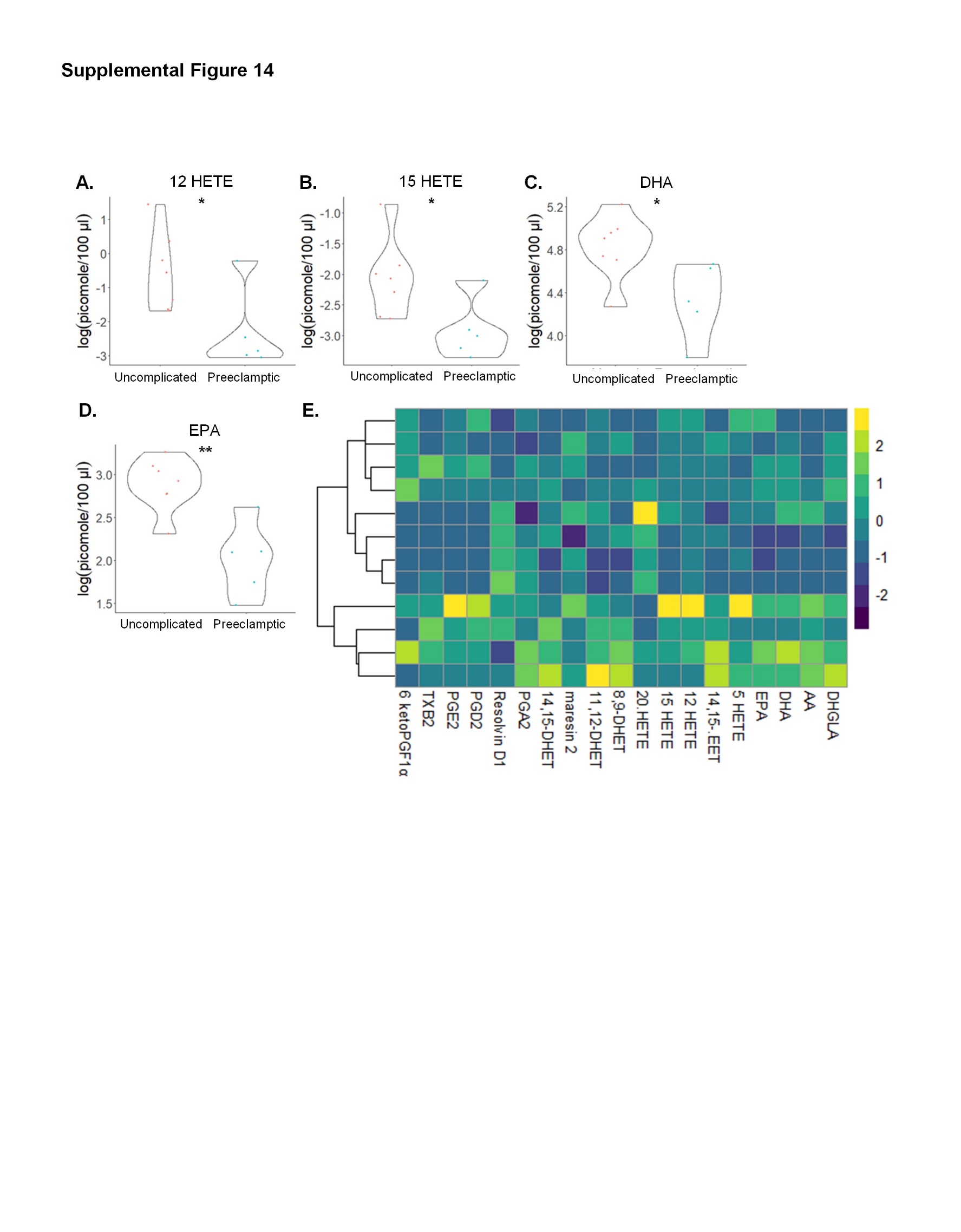


**Supplemental Figure 14. Distinct eicosanoids show significant differences in the plasma from uncomplicated term pregnant patients of Western European descent vs. PE patients of Western European descent. (A-D**) Eicosanoid species that occurred at significantly different levels when comparing plasma from uncomplicated term pregnant patients of Western European to patients of Western European with PE using UPLC ESI-MS/MS as the detection method. Samples were analyzed by UPLC ESI-MS/MS within two weeks of acquisition. **(E)** Heatmap of all Eicosanoid species that were detected via UPLC ESI-MS/MS in plasma (fold change is depicted). Samples were compared using unpaired students t-test with Welch’s correction. Data shown are means + SD depicted as violin plots, *P< 0.05, **P< 0.01, ***P< 0.001, ****P< 0.0001.


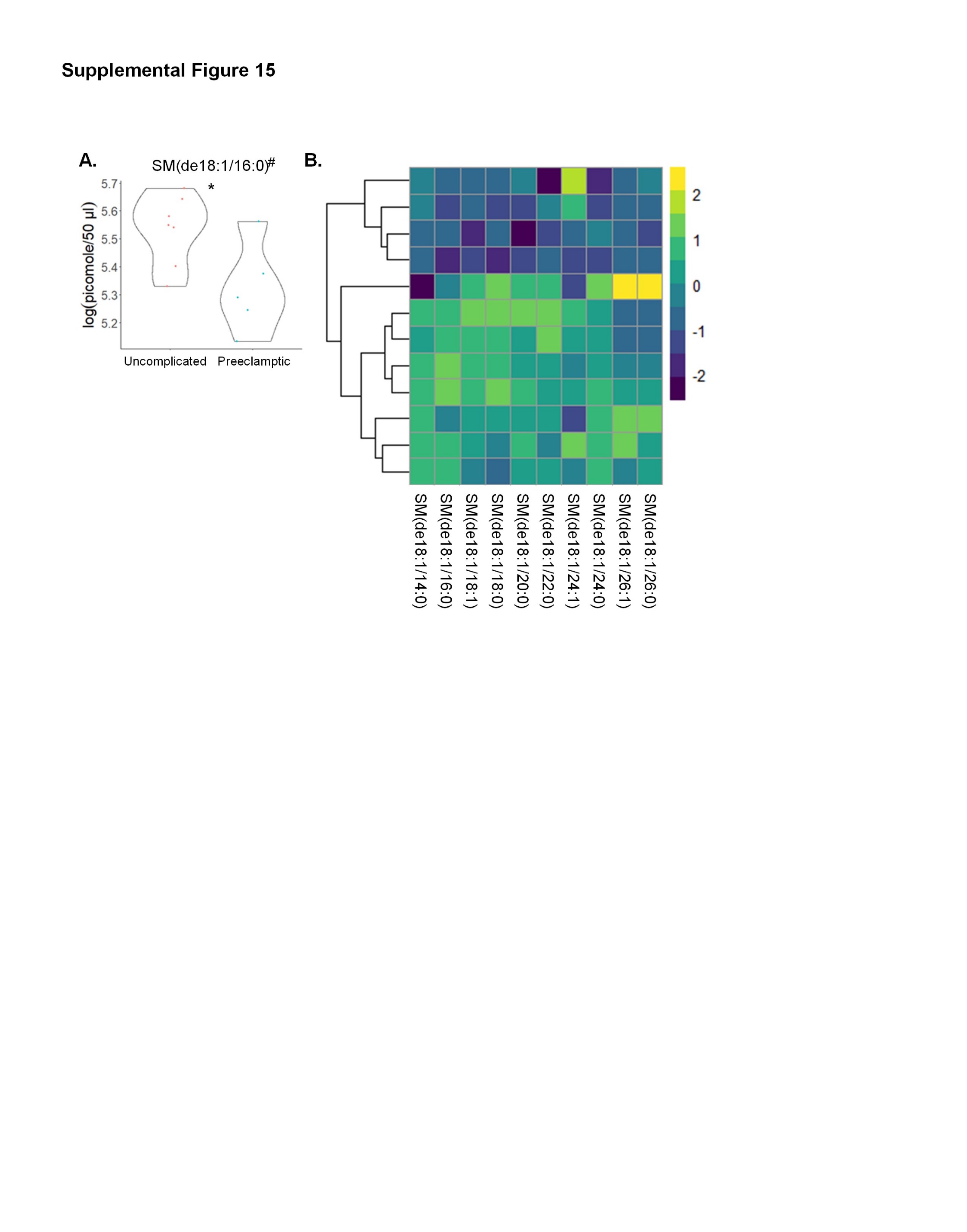


**Supplemental Figure 15. Sphingomyelin levels show significant differences in the plasma from uncomplicated term pregnant patients of Western European descent vs. PE patients of Western European descent. (A)** Sphingomyelin species that occurred at significantly different levels when comparing plasma from uncomplicated term pregnant patients of Western European to patients of Western European with PE using UPLC ESI-MS/MS as the detection method. Samples were analyzed by UPLC ESI-MS/MS within two weeks of acquisition. **(B)** Heatmap of all sphingomyelin species that were detected via UPLC ESI-MS/MS in plasma (fold change is depicted). Samples were compared using unpaired students t-test with Welch’s correction. Data shown are means + SD depicted as violin plots, *P< 0.05, **P< 0.01, ***P< 0.001, ****P< 0.0001. Non-transformed data were also analyzed by the Wilcoxon Sum Rank Test. Bioactive lipid mediators not found to be significantly different by the Wilcoxon Sum Rank Test are designated with a #.


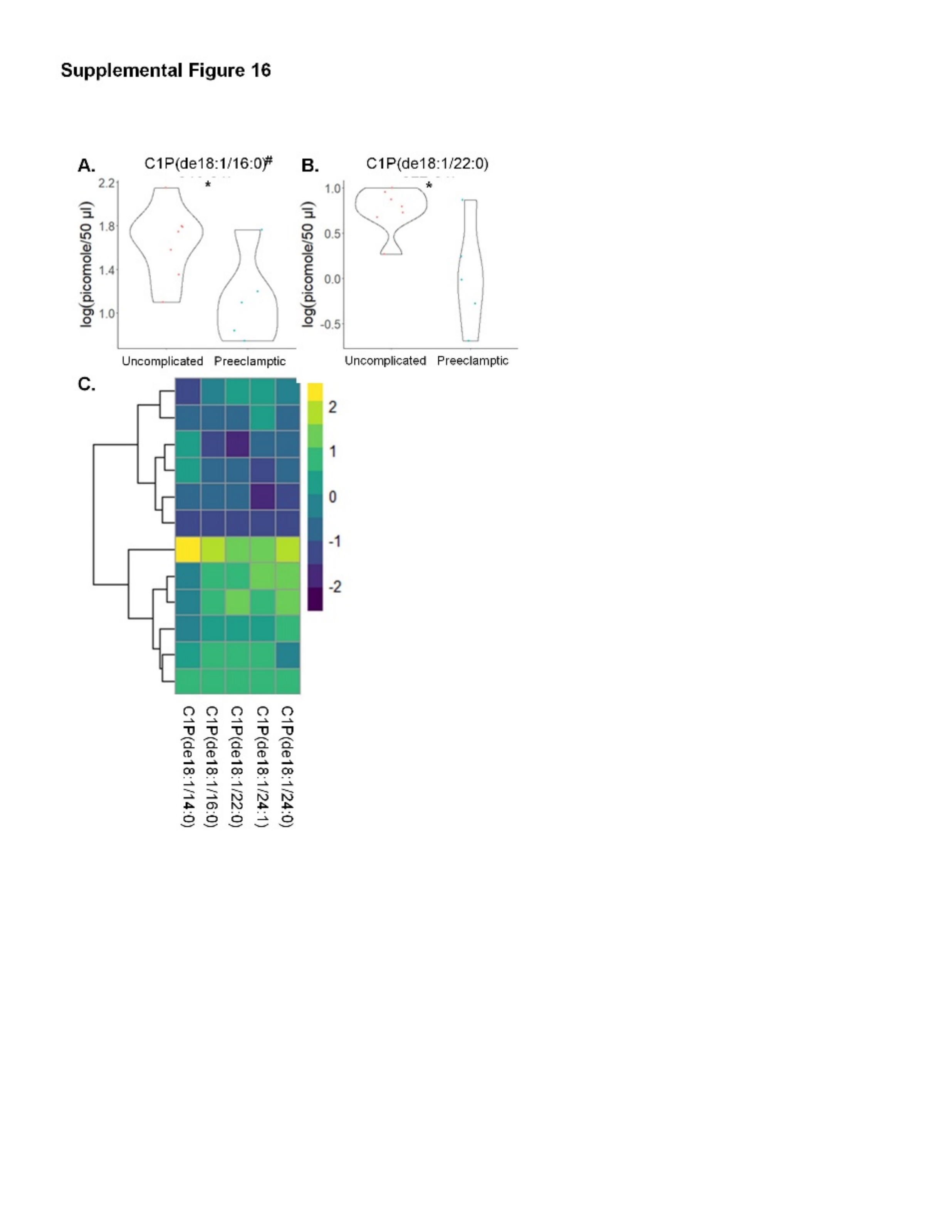


**Supplemental Figure 16. C1P levels show significant differences in the plasma from uncomplicated term pregnant patients of Western European descent vs. PE patients of Western European descent. (A,B**) C1P species that occurred at significantly different levels when comparing plasma from uncomplicated term pregnant patients of Western European to patients of Western European with PE using UPLC ESI-MS/MS as the detection method. Samples were analyzed by UPLC ESI-MS/MS within two weeks of acquisition. **(C)** Heatmap of all C1P species that were detected via UPLC ESI-MS/MS in plasma (fold change is depicted). Samples were compared using unpaired students t-test with Welch’s correction. Data shown are means + SD depicted as violin plots, *P< 0.05, **P< 0.01, ***P< 0.001, ****P< 0.0001. Non-transformed data were also analyzed by the Wilcoxon Sum Rank Test. Bioactive lipid mediators not found to be significantly different by the Wilcoxon Sum Rank Test are designated with a #.


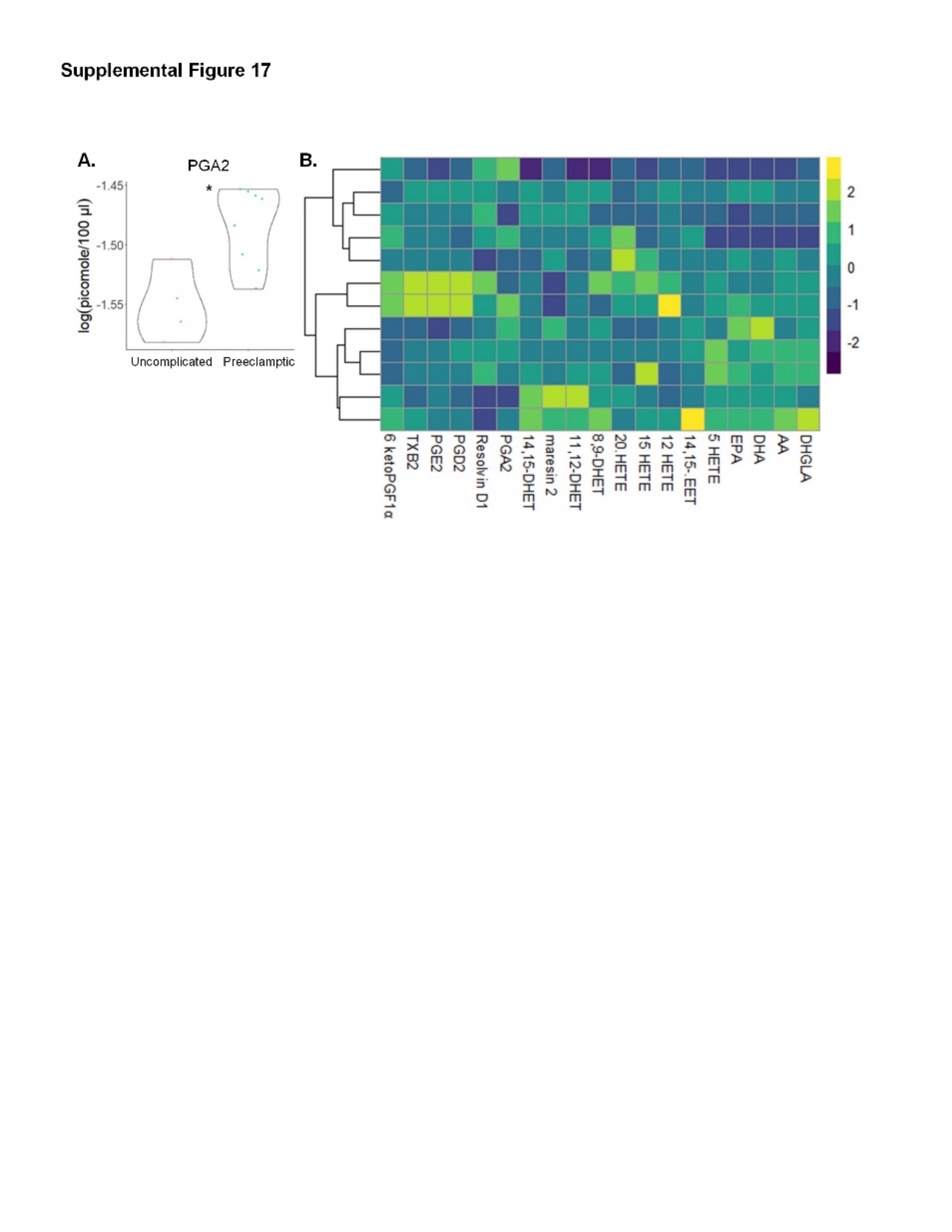
**Supplemental Figure 17. Distinct eicosanoids show significant differences in the plasma from uncomplicated term pregnant patients of Hispanic descent vs. PE patients of Hispanic descent. (A**) Eicosanoid species that occurred at significantly different levels when comparing plasma from uncomplicated term pregnant patients of Hispanic descent to patients of Hispanic descent with PE using UPLC ESI-MS/MS as the detection method. Samples were analyzed by UPLC ESI-MS/MS within two weeks of acquisition. **(B)** Heatmap of all Eicosanoid species that were detected via UPLC ESI-MS/MS in plasma (fold change is depicted). Samples were compared using unpaired students t-test with Welch’s correction. Data shown are means + SD depicted as violin plots, *P< 0.05, **P< 0.01, ***P< 0.001, ****P< 0.0001.


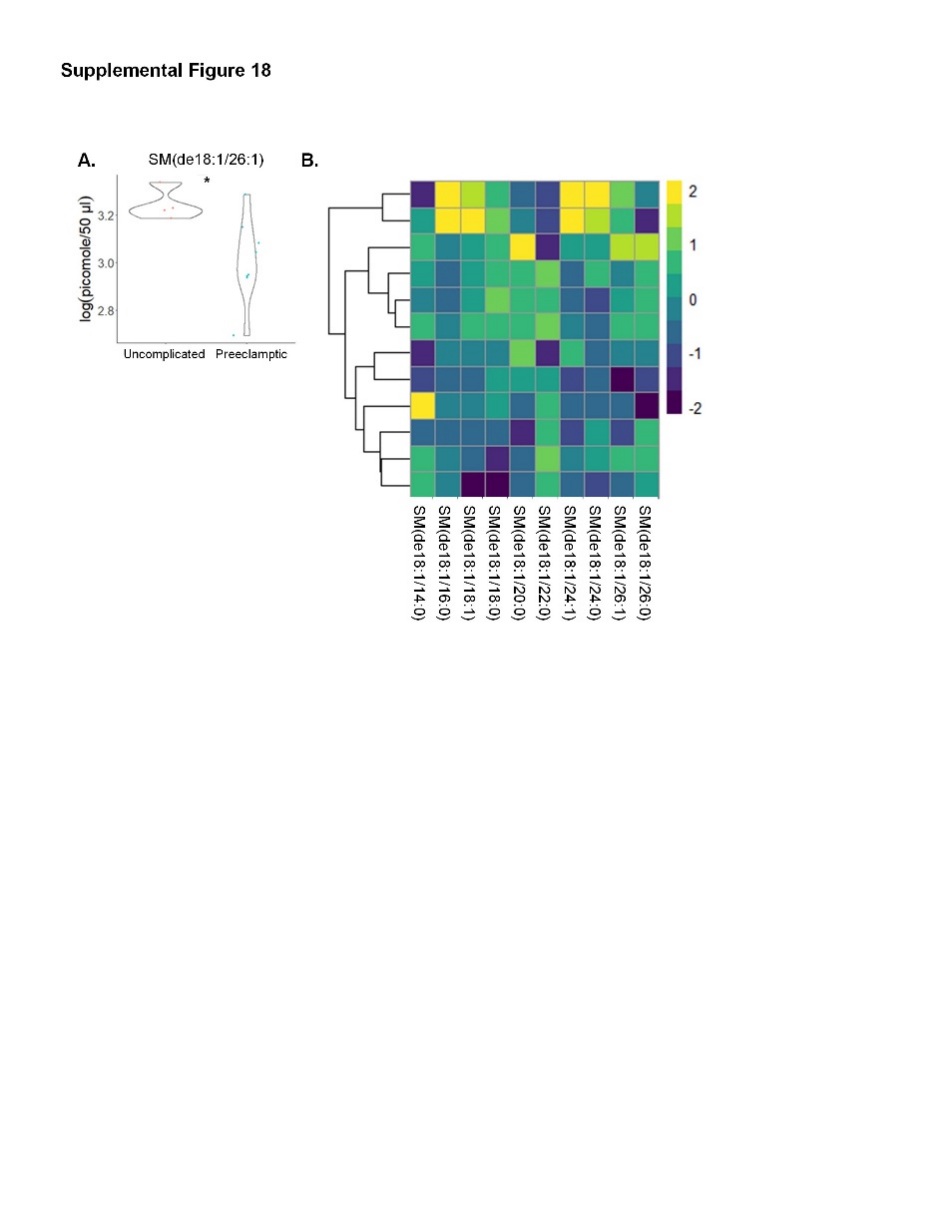


**Supplemental Figure 18. Sphingomyelin levels show significant differences in the plasma from uncomplicated term pregnant patients of Hispanic descent vs. PE patients of Hispanic descent. (A)** Sphingomyelin species that occurred at significantly different levels when comparing plasma from uncomplicated term pregnant patients of Hispanic descent to patients of Hispanic descent with PE using UPLC ESI-MS/MS as the detection method. Samples were analyzed by UPLC ESI-MS/MS within two weeks of acquisition. **(B)** Heatmap of all sphingomyelin species that were detected via UPLC ESI-MS/MS in plasma (fold change is depicted). Samples were compared using unpaired students t-test with Welch’s correction. Data shown are means + SD depicted as violin plots, *P< 0.05, **P< 0.01, ***P< 0.001, ****P< 0.0001.


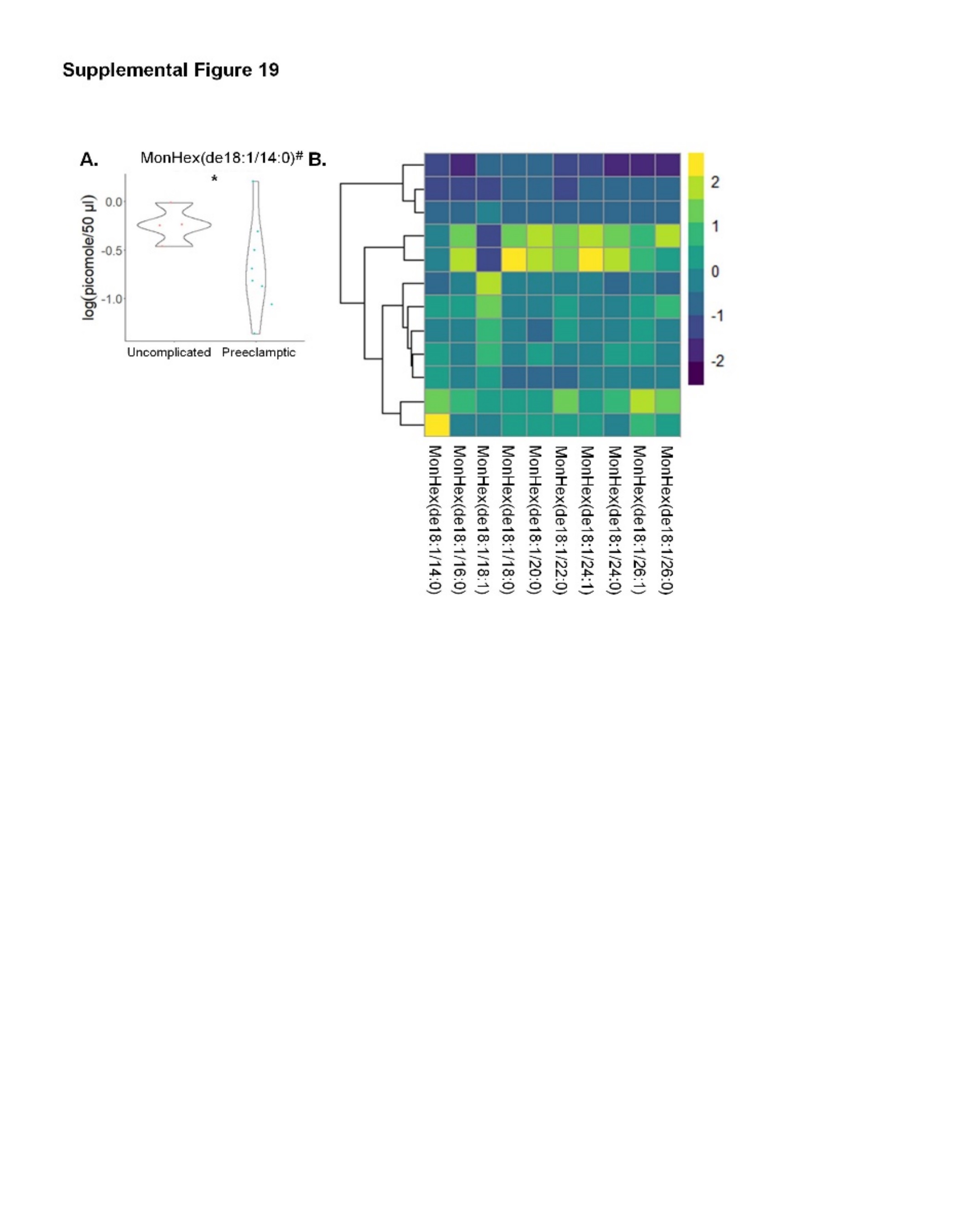


**Supplemental Figure 19. MonHex species show significant differences in the plasma from uncomplicated term pregnant patients of Hispanic descent vs. PE patients of Hispanic descent. (A**) MonHex species that occurred at significantly different levels when comparing plasma from uncomplicated term pregnant patients of Hispanic descent to patients of Hispanic descent with PE using UPLC ESI-MS/MS as the detection method. Samples were analyzed by UPLC ESI-MS/MS within two weeks of acquisition. **(B)** Heatmap of all MonHex species that were detected via UPLC ESI-MS/MS in plasma (fold change is depicted). Samples were compared using unpaired students t-test with Welch’s correction. Data shown are means + SD depicted as violin plots, *P< 0.05, **P< 0.01, ***P< 0.001, ****P< 0.0001. Non-transformed data were also analyzed by the Wilcoxon Sum Rank Test. Bioactive lipid mediators not found to be significantly different by the Wilcoxon Sum Rank Test are designated with a #.


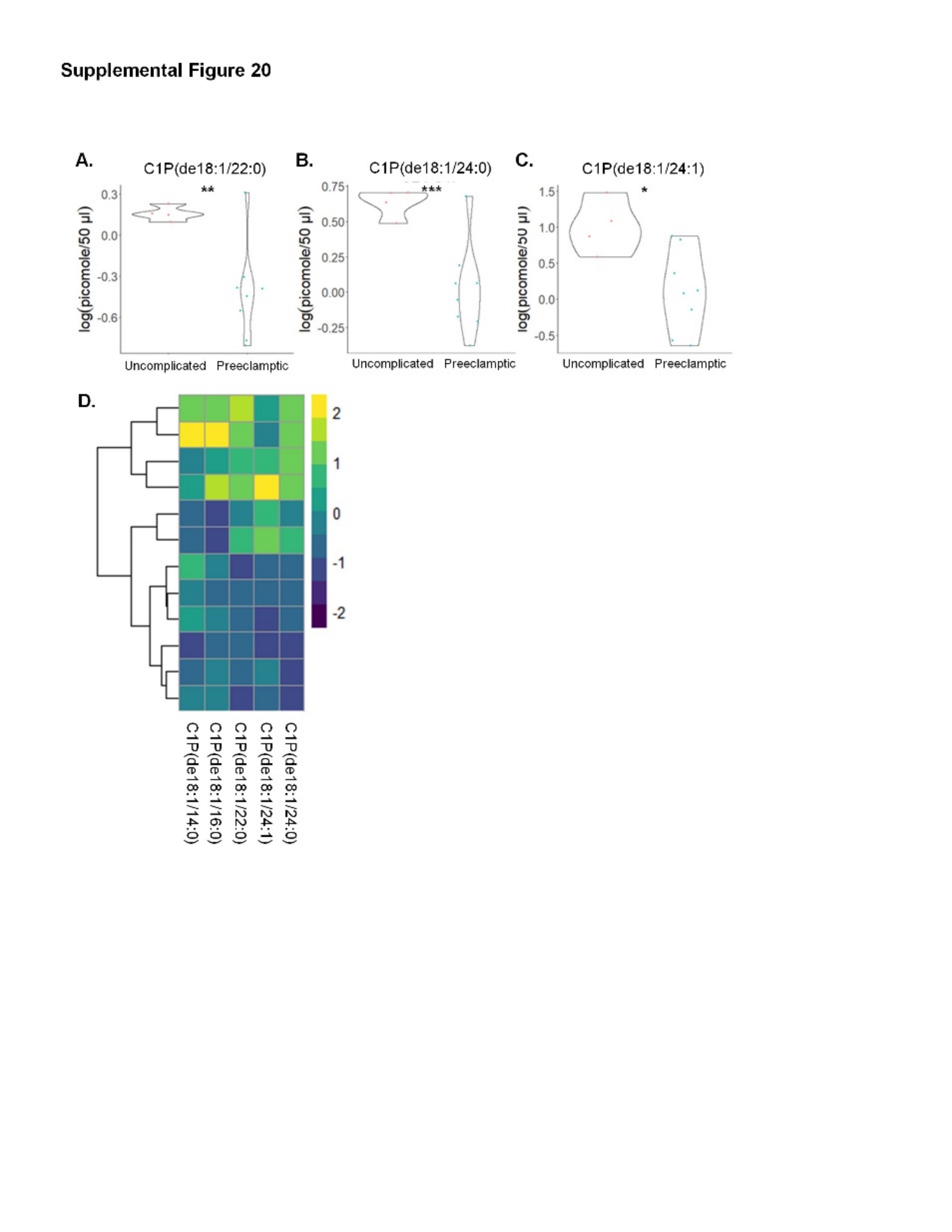


**Supplemental Figure 20. C1P levels show significant differences in the plasma from uncomplicated term pregnant patients of Hispanic descent vs. PE patients of Hispanic descent. (A-C**) C1P species that occurred at significantly different levels when comparing plasma from uncomplicated term pregnant patients of Hispanic descent to patients of Hispanic descent with PE using UPLC ESI-MS/MS as the detection method. Samples were analyzed by UPLC ESI-MS/MS within two weeks of acquisition. **(D)** Heatmap of all C1P species that were detected via UPLC ESI-MS/MS in plasma (fold change is depicted). Samples were compared using unpaired students t-test with Welch’s correction. Data shown are means + SD depicted as violin plots, *P< 0.05, **P< 0.01, ***P< 0.001, ****P< 0.0001.


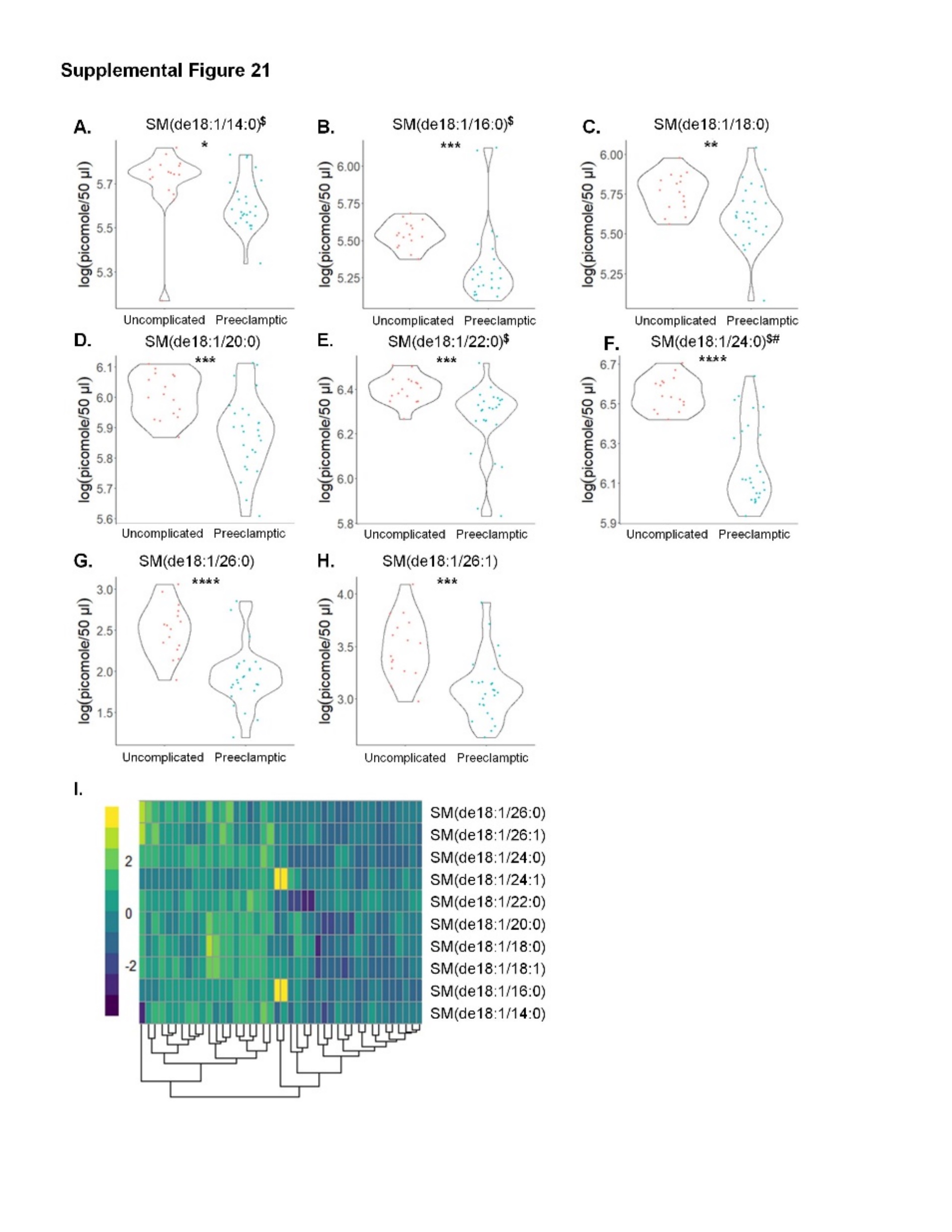


**Supplemental Figure 21. Sphingomyelin levels show significant differences in the plasma from uncomplicated term pregnant patients recruited from the general OB/GYN clinic vs. total PE pregnant patients. (A-H)** Sphingomyelin species that occurred at significantly different levels when comparing plasma from uncomplicated term pregnant patients recruited from the general OB/GYN clinic vs. total PE pregnant patients using UPLC ESI-MS/MS as the detection method. Samples were analyzed by UPLC ESI-MS/MS within two weeks of acquisition. **(I)** Heatmap of all sphingomyelin species that were detected via UPLC ESI-MS/MS in plasma (depicted as fold change). Samples were compared using unpaired students t-test with Welch’s correction. Data shown are means + SD depicted as violin plots, *P< 0.05, **P< 0.01, ***P< 0.001, ****P< 0.0001. The log transformed data failing the Shapiro-Wilk Test are designated with a $. Non-transformed data were also analyzed by the Wilcoxon Sum Rank Test. Bioactive lipid mediators not found to be significantly different by the Wilcoxon Sum Rank Test are designated with a #.


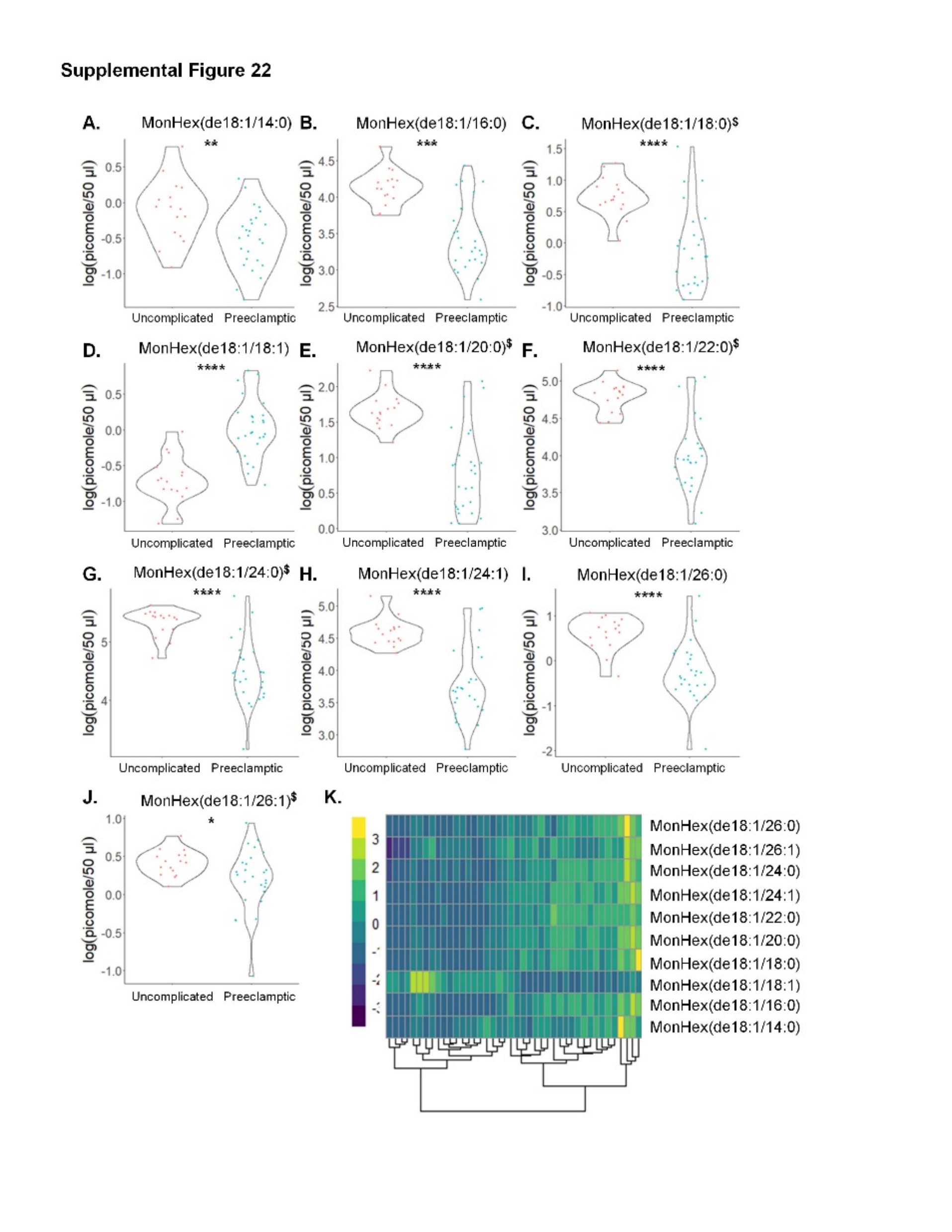


**Supplemental Figure 22. MonHex species show significant differences in the plasma from uncomplicated term pregnant patients recruited from the general OB/GYN clinic vs. total PE pregnant patients. (A-J**) MonHex species that occurred at significantly different levels when comparing plasma from uncomplicated term pregnant patients recruited from the general OB/GYN clinic vs. total PE pregnant patients using UPLC ESI-MS/MS as the detection method. Samples were analyzed by UPLC ESI-MS/MS within two weeks of acquisition. **(K)** Heatmap of all MonHex species that were detected via UPLC ESI-MS/MS in plasma (depicted as fold change). Samples were compared using unpaired students t-test with Welch’s correction. Data shown are means + SD depicted as violin plots, *P< 0.05, **P< 0.01, ***P< 0.001, ****P< 0.0001. The log transformed data failing the Shapiro-Wilk Test are designated with a $.


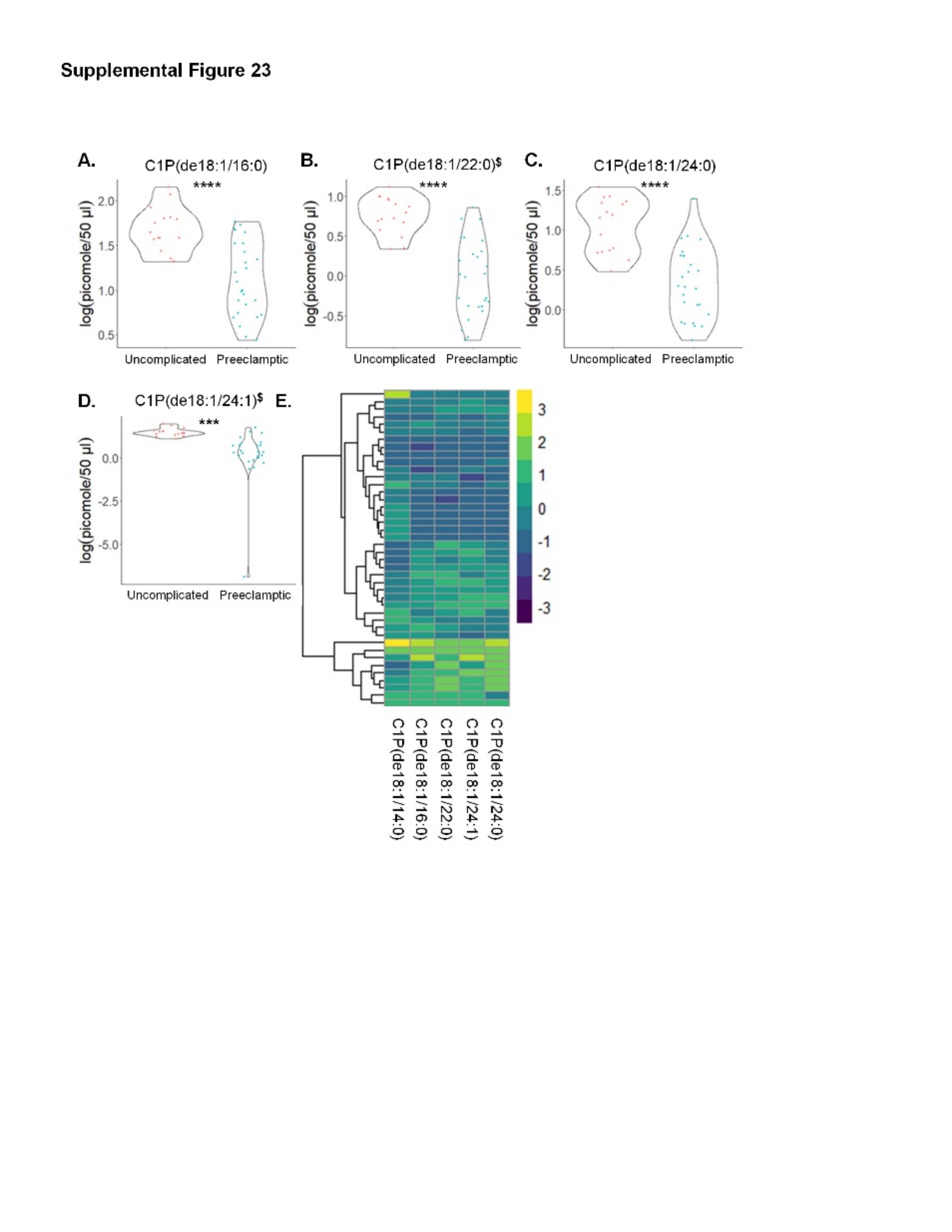


**Supplemental Figure 23. C1P levels show significant differences in the plasma from uncomplicated term pregnant patients recruited from the general OB/GYN clinic vs. total PE pregnant patients. (A-D**) C1P species that occurred at significantly different levels when comparing plasma from uncomplicated term pregnant patients recruited from the general OB/GYN clinic vs. total PE pregnant patients using UPLC ESI-MS/MS as the detection method. Samples were analyzed by UPLC ESI-MS/MS within two weeks of acquisition. **(E)** Heatmap of all C1P species that were detected via UPLC ESI-MS/MS in plasma (depicted as fold change). Samples were compared using unpaired students t-test with Welch’s correction. Data shown are means + SD depicted as violin plots, *P< 0.05, **P< 0.01, ***P< 0.001, ****P< 0.0001. The log transformed data failing the Shapiro-Wilk Test are designated with a $.


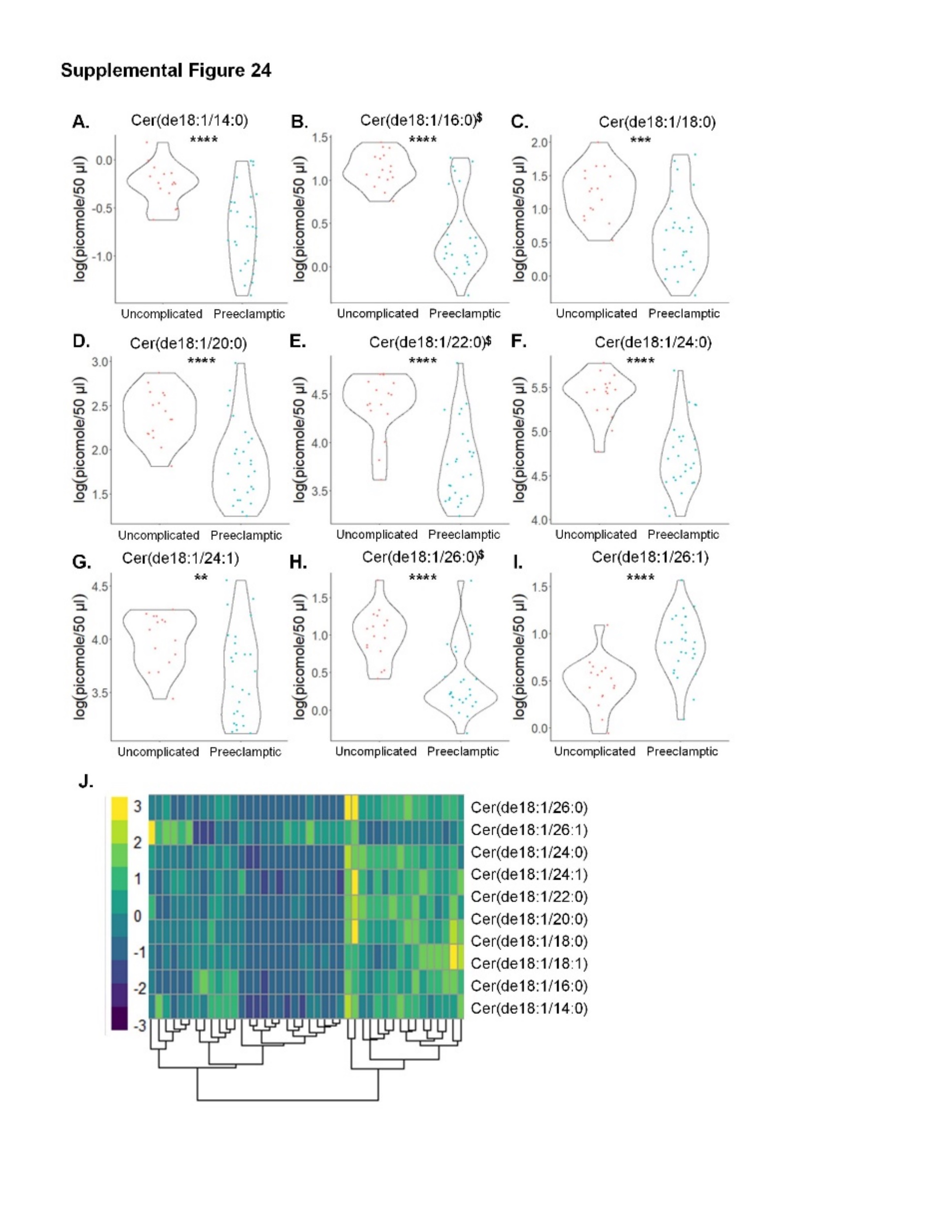


**Supplemental Figure 24. Ceramide species show significant differences in the plasma from uncomplicated term pregnant patients recruited from the general OB/GYN clinic vs. total PE pregnant patients. (A-I**) Ceramide species that occurred at significantly different levels when comparing plasma from uncomplicated term pregnant patients recruited from the general OB/GYN clinic vs. total PE pregnant patients using UPLC ESI-MS/MS as the detection method. Samples were analyzed by UPLC ESI-MS/MS within two weeks of acquisition. **(J)** Heatmap of all Ceramide species that were detected via UPLC ESI-MS/MS in plasma (depicted as fold change). Samples were compared using unpaired students t-test with Welch’s correction. Data shown are means + SD depicted as violin plots, *P< 0.05, **P< 0.01, ***P< 0.001, ****P< 0.0001. The log transformed data failing the Shapiro-Wilk Test are designated with a $.


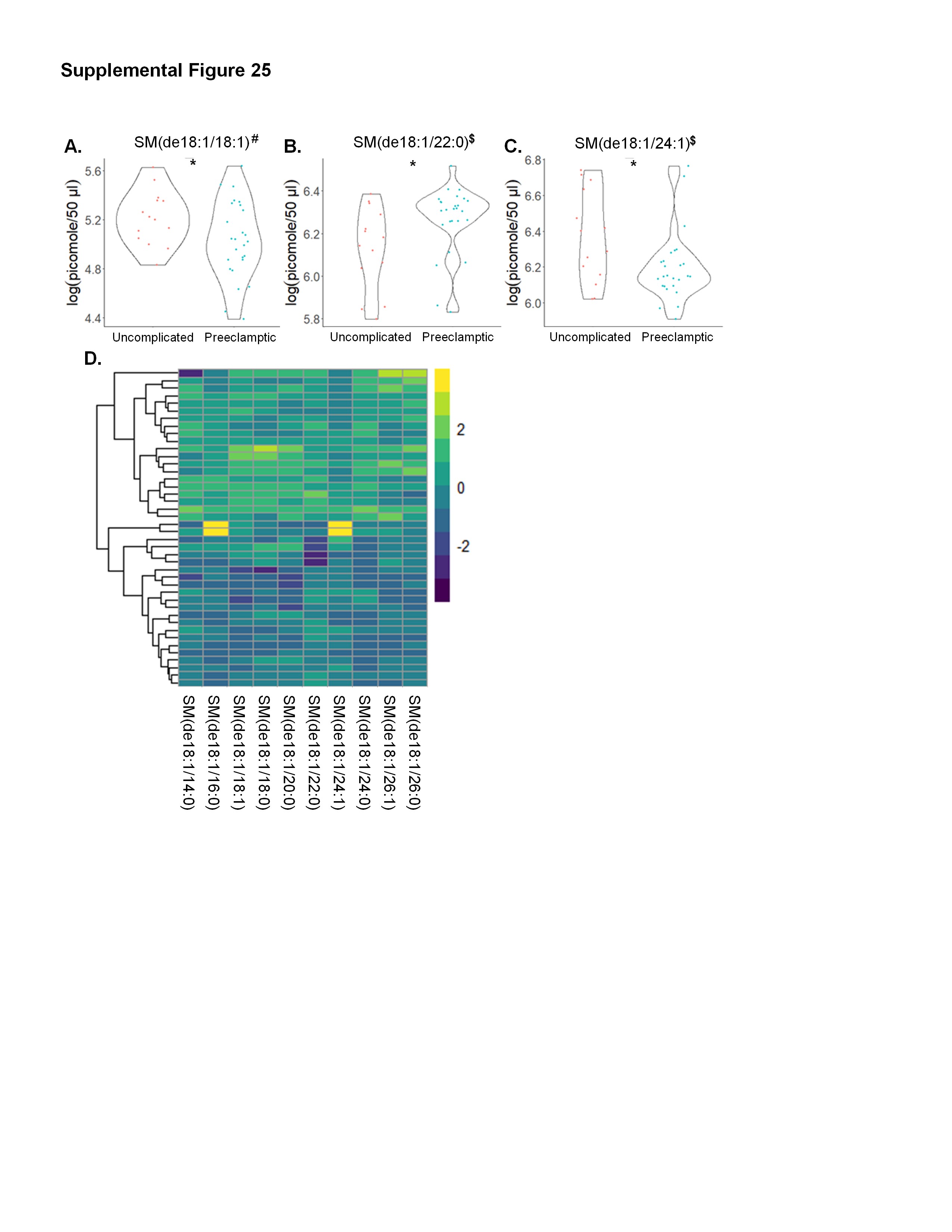


**Supplemental Figure 25. Sphingomyelin levels show significant differences in the plasma from uncomplicated term pregnant patients referred to High-Risk OB/GYN clinic vs. total PE pregnant patients. (A-C)** Sphingomyelin species that occurred at significantly different levels when comparing plasma from uncomplicated term pregnant patients referred to High-Risk OB/GYN clinic vs. total PE pregnant patients using UPLC ESI-MS/MS as the detection method. Samples were analyzed by UPLC ESI-MS/MS within two weeks of acquisition. **(D)** Heatmap of all sphingomyelin species that were detected via UPLC ESI-MS/MS in plasma (depicted as fold change). Samples were compared using unpaired students t-test with Welch’s correction. Data shown are means + SD depicted as violin plots, *P< 0.05, **P< 0.01, ***P< 0.001, ****P< 0.0001. The log transformed data failing the Shapiro-Wilk Test are designated with a $. Non-transformed data were also analyzed by the Wilcoxon Sum Rank Test. Bioactive lipid mediators not found to be significantly different by the Wilcoxon Sum Rank Test are designated with a #.


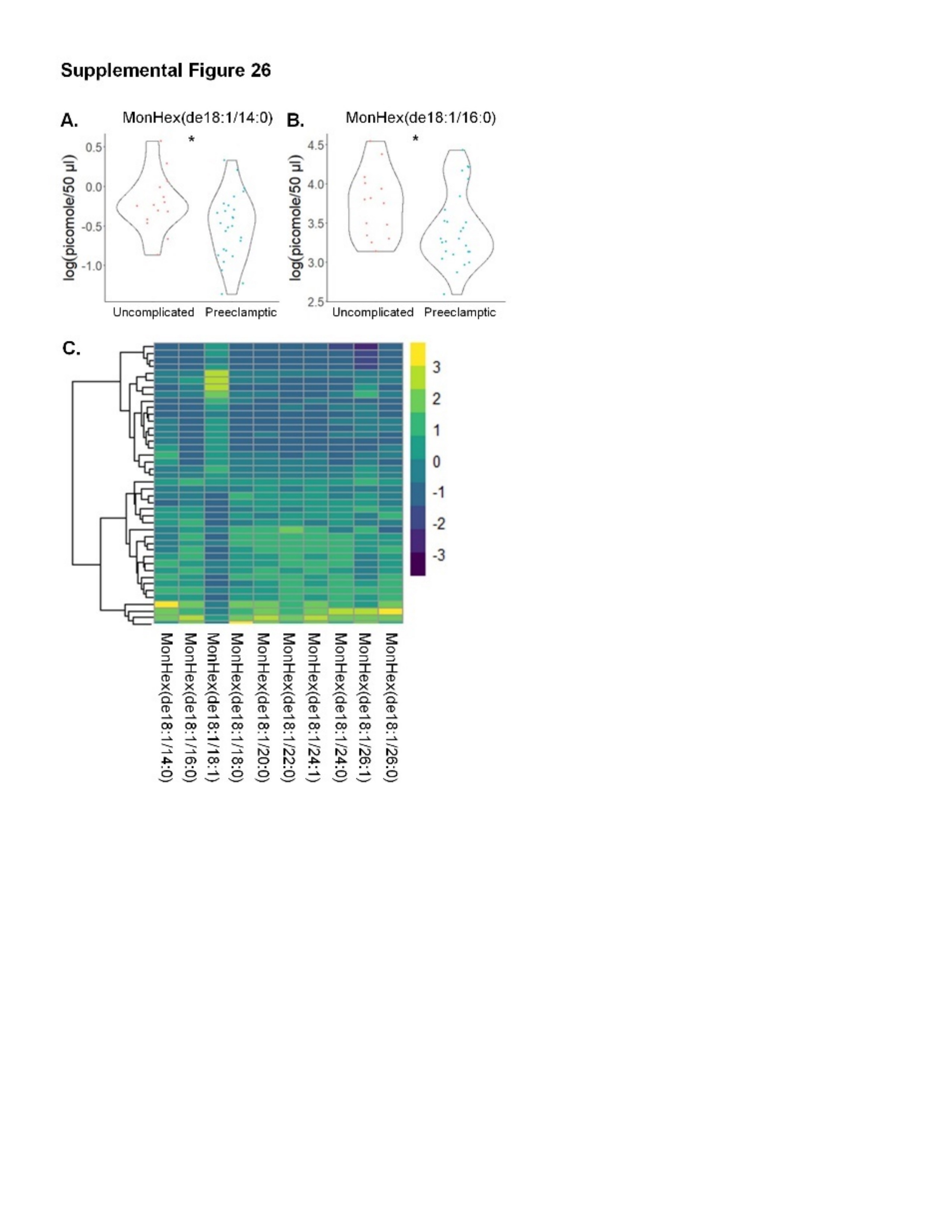


**Supplemental Figure 26. MonHex species show significant differences in the plasma from uncomplicated term pregnant patients referred to High-Risk OB/GYN clinic vs. total PE pregnant patients. (A,B**) MonHex species that occurred at significantly different levels when comparing plasma from uncomplicated term pregnant patients referred to High-Risk OB/GYN clinic vs. total PE pregnant patients using UPLC ESI-MS/MS as the detection method. Samples were analyzed by UPLC ESI-MS/MS within two weeks of acquisition. **(C)** Heatmap of all MonHex species that were detected via UPLC ESI-MS/MS in plasma (depicted as fold change). Samples were compared using unpaired students t-test with Welch’s correction. Data shown are means + SD depicted as violin plots, *P< 0.05, **P< 0.01, ***P< 0.001, ****P< 0.0001.


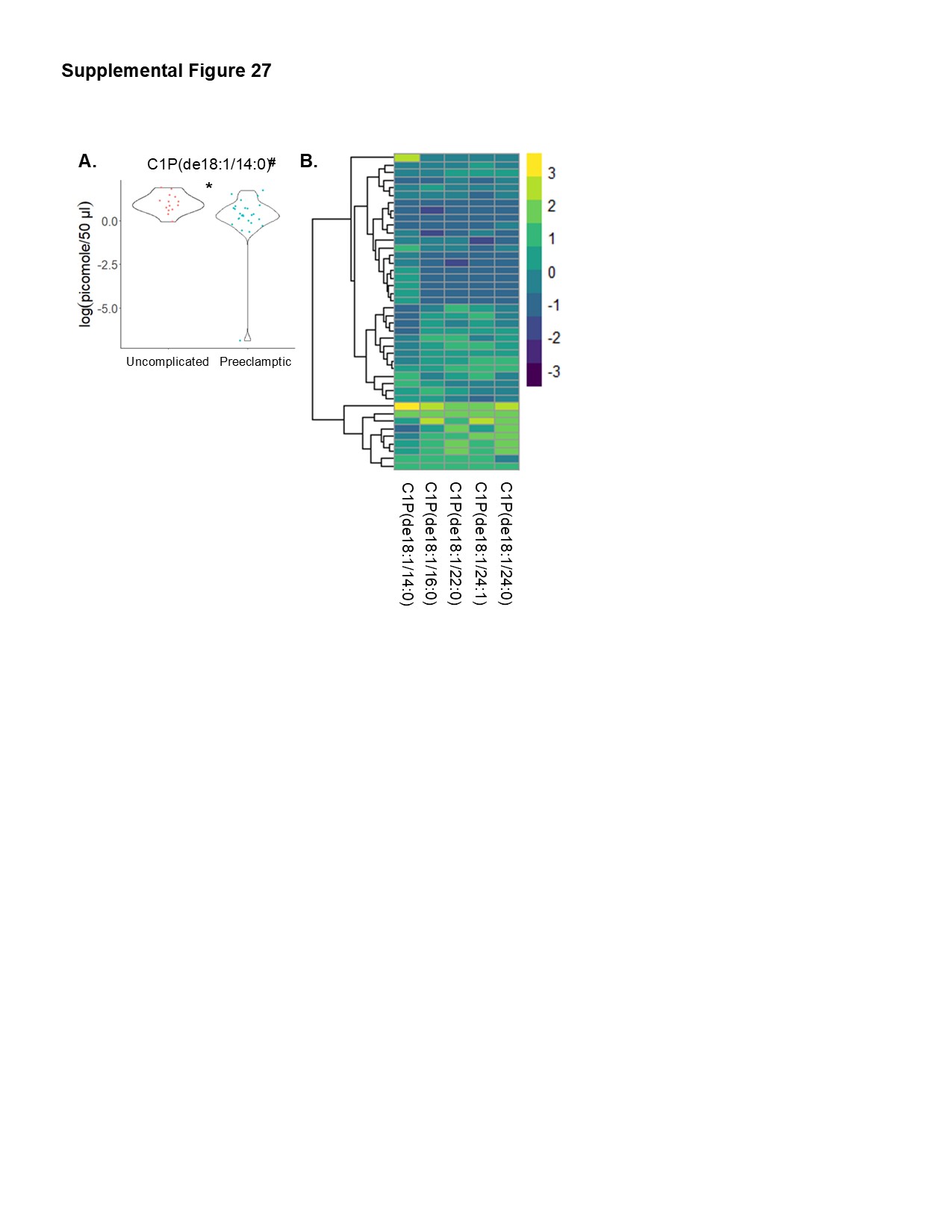


**Supplemental Figure 27. C1P levels show significant differences in the plasma from uncomplicated term pregnant patients referred to High-Risk OB/GYN clinic vs. total PE pregnant patients. (A**) C1P species that occurred at significantly different levels when comparing plasma from uncomplicated term pregnant patients referred to High-Risk OB/GYN clinic vs. total PE pregnant patients using UPLC ESI-MS/MS as the detection method. Samples were analyzed by UPLC ESI-MS/MS within two weeks of acquisition. **(B)** Heatmap of all C1P species that were detected via UPLC ESI-MS/MS in plasma (depicted as fold change). Samples were compared using unpaired students t-test with Welch’s correction. Data shown are means + SD depicted as violin plots, *P< 0.05, **P< 0.01, ***P< 0.001, ****P< 0.0001. Non-transformed data were also analyzed by the Wilcoxon Sum Rank Test. Bioactive lipid mediators not found to be significantly different by the Wilcoxon Sum Rank Test are designated with a #.


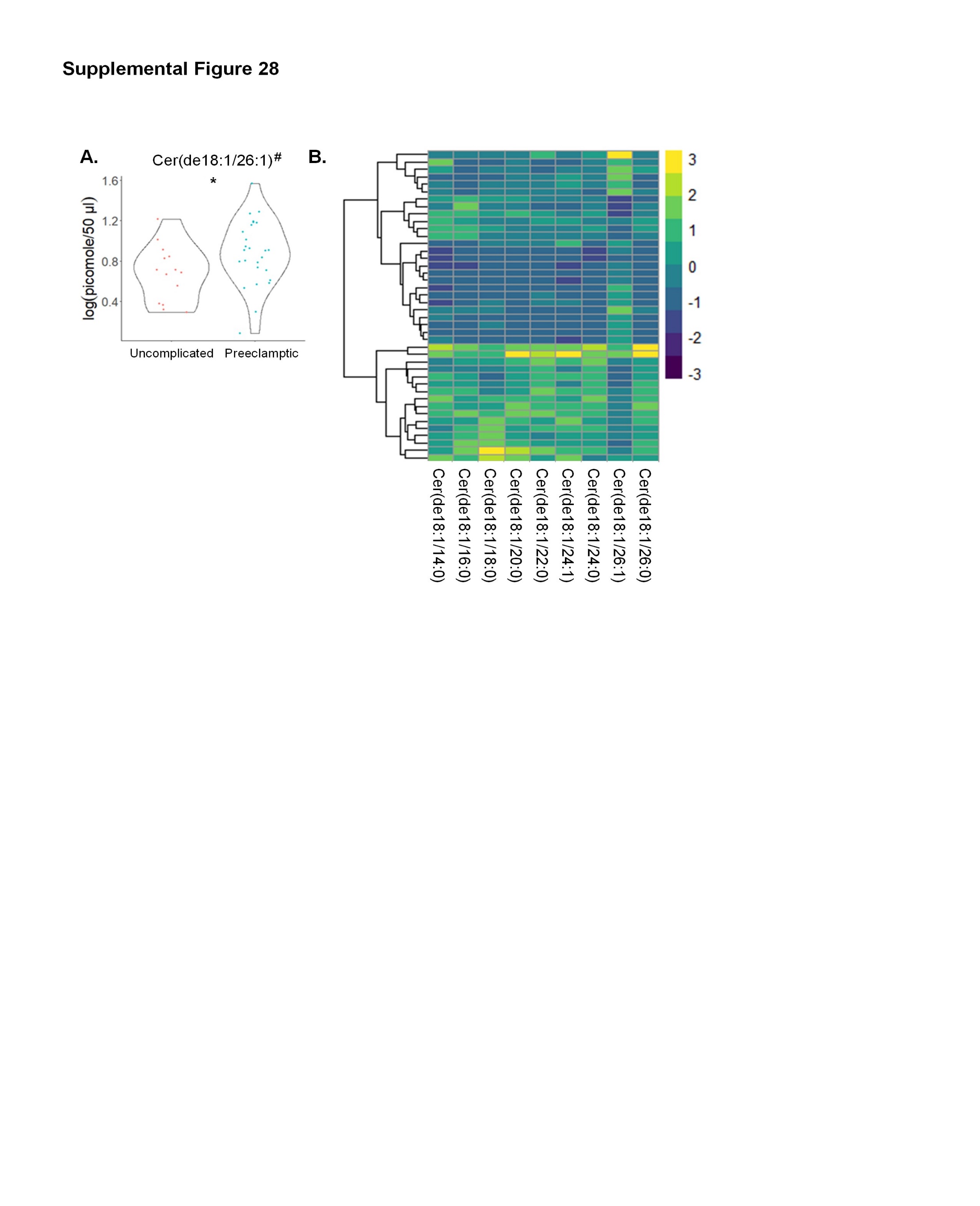


**Supplemental Figure 28. Ceramide species show significant differences in the plasma from uncomplicated term pregnant patients referred to High-Risk OB/GYN clinic vs. total PE pregnant patients. (A**) Ceramide species that occurred at significantly different levels when comparing plasma from uncomplicated term pregnant patients referred to High-Risk OB/GYN clinic vs. total PE pregnant patients using UPLC ESI-MS/MS as the detection method. Samples were analyzed by UPLC ESI-MS/MS within two weeks of acquisition. **(B)** Heatmap of all Ceramide species that were detected via UPLC ESI-MS/MS in plasma (depicted as fold change). Samples were compared using unpaired students t-test with Welch’s correction. Data shown are means + SD depicted as violin plots, *P< 0.05, **P< 0.01, ***P< 0.001, ****P< 0.0001. Non-transformed data were also analyzed by the Wilcoxon Sum Rank Test. Bioactive lipid mediators not found to be significantly different by the Wilcoxon Sum Rank Test are designated with a #.


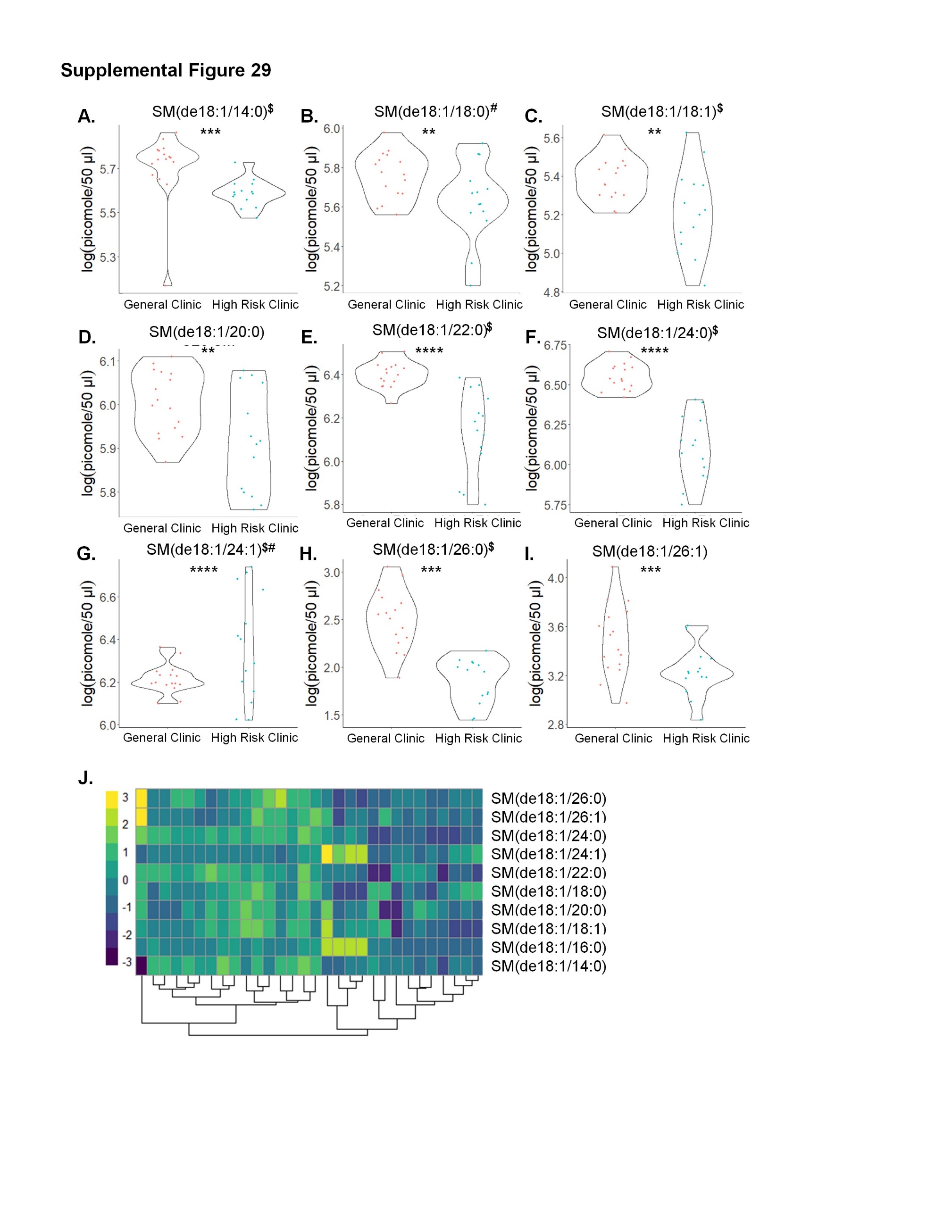


**Supplemental Figure 29. Sphingomyelin levels show significant differences in the plasma from uncomplicated term pregnant patients recruited from General OB/GYN clinic vs. uncomplicated term pregnant patients referred to High-Risk OB/GYN clinic. (A-I)** Sphingomyelin species that occurred at significantly different levels when comparing plasma from normal uncomplicated term pregnant patients recruited from General OB/GYN clinic vs. normal uncomplicated term pregnant patients referred to High-Risk OB/GYN clinic using UPLC ESI-MS/MS as the detection method. Samples were analyzed by UPLC ESI-MS/MS within two weeks of acquisition. **(J)** Heatmap of all sphingomyelin species that were detected via UPLC ESI-MS/MS in plasma (depicted as fold change). Samples were compared using unpaired students t-test with Welch’s correction. Data shown are means + SD depicted as violin plots, *P< 0.05, **P< 0.01, ***P< 0.001, ****P< 0.0001. The log transformed data failing the Shapiro-Wilk Test are designated with a $. Non-transformed data were also analyzed by the Wilcoxon Sum Rank Test. Bioactive lipid mediators not found to be significantly different by the Wilcoxon Sum Rank Test are designated with a #.


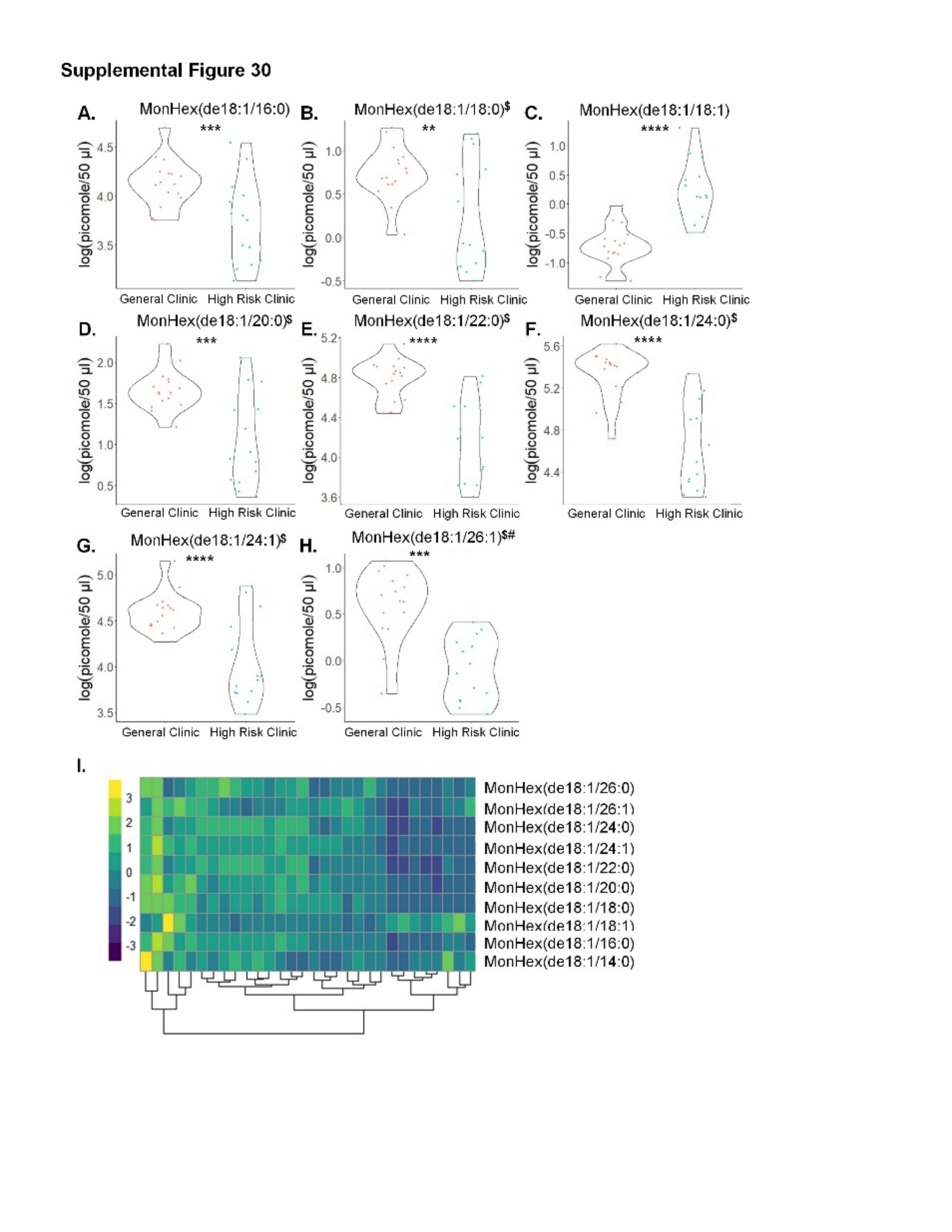


**Supplemental Figure 30. MonHex species show significant differences in the plasma from uncomplicated term pregnant patients recruited from General OB/GYN clinic vs. uncomplicated term pregnant patients referred to High-Risk OB/GYN clinic. (A-H**) MonHex species that occurred at significantly different levels when comparing plasma from normal uncomplicated term pregnant patients recruited from General OB/GYN clinic vs. normal uncomplicated term pregnant patients referred to High-Risk OB/GYN clinic using UPLC ESI-MS/MS as the detection method. Samples were analyzed by UPLC ESI-MS/MS within two weeks of acquisition. **(I)** Heatmap of all MonHex species that were detected via UPLC ESI-MS/MS in plasma (depicted as fold change). Samples were compared using unpaired students t-test with Welch’s correction. Data shown are means + SD depicted as violin plots, *P< 0.05, **P< 0.01, ***P< 0.001, ****P< 0.0001. The log transformed data failing the Shapiro-Wilk Test are designated with a $. Non-transformed data were also analyzed by the Wilcoxon Sum Rank Test. Bioactive lipid mediators not found to be significantly different by the Wilcoxon Sum Rank Test are designated with a #.


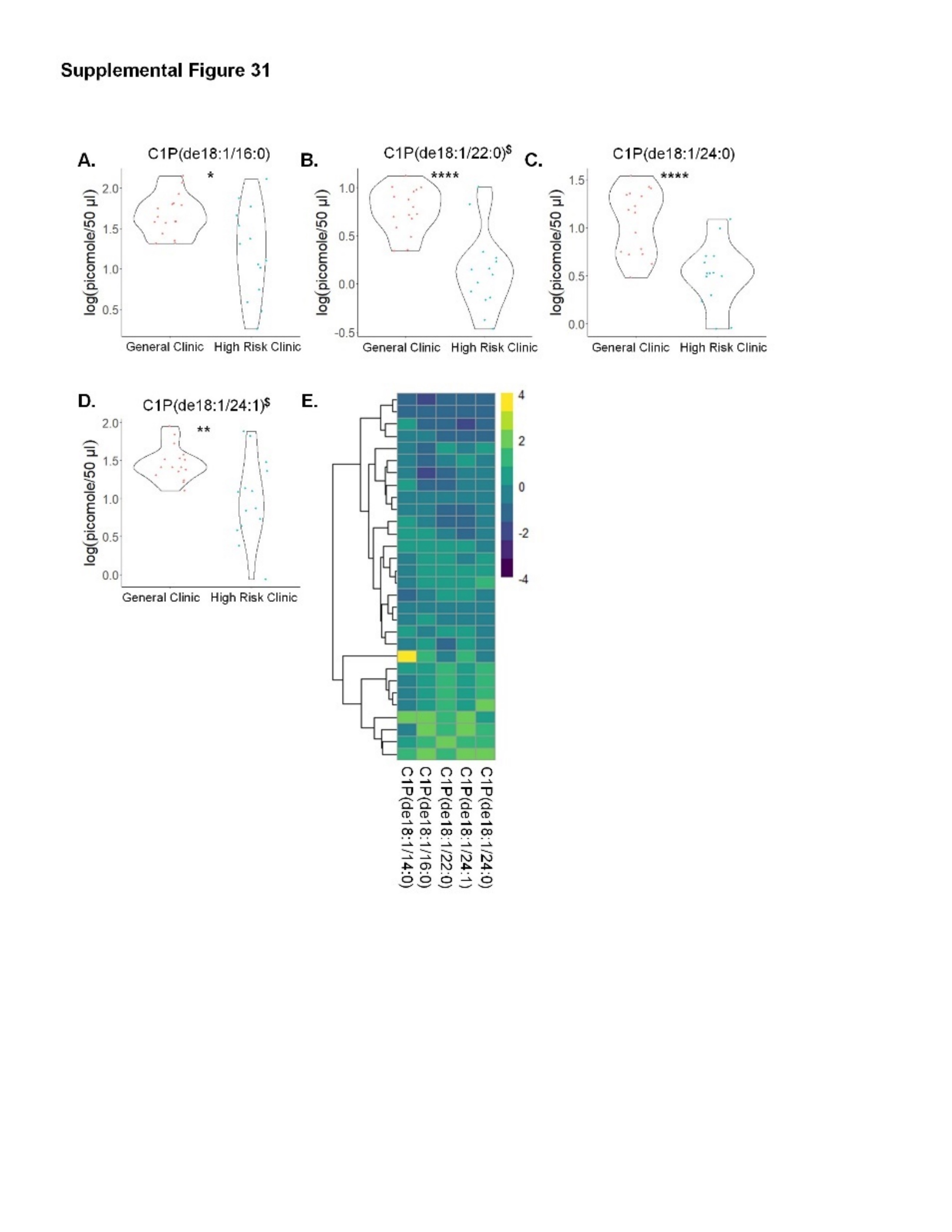


**Supplemental Figure 31. C1P levels show significant differences in the plasma from uncomplicated term pregnant patients recruited from General OB/GYN clinic vs. uncomplicated term pregnant patients referred to High-Risk OB/GYN clinic. (A-D**) C1P species that occurred at significantly different levels when comparing plasma from normal uncomplicated term pregnant patients recruited from General OB/GYN clinic vs. normal uncomplicated term pregnant patients referred to High-Risk OB/GYN clinic using UPLC ESI-MS/MS as the detection method. Samples were analyzed by UPLC ESI-MS/MS within two weeks of acquisition. **(E)** Heatmap of all C1P species that were detected via UPLC ESI-MS/MS in plasma (depicted as fold change). Samples were compared using unpaired students t-test with Welch’s correction. Data shown are means + SD depicted as violin plots, *P< 0.05, **P< 0.01, ***P< 0.001, ****P< 0.0001. The log transformed data failing the Shapiro-Wilk Test are designated with a $.


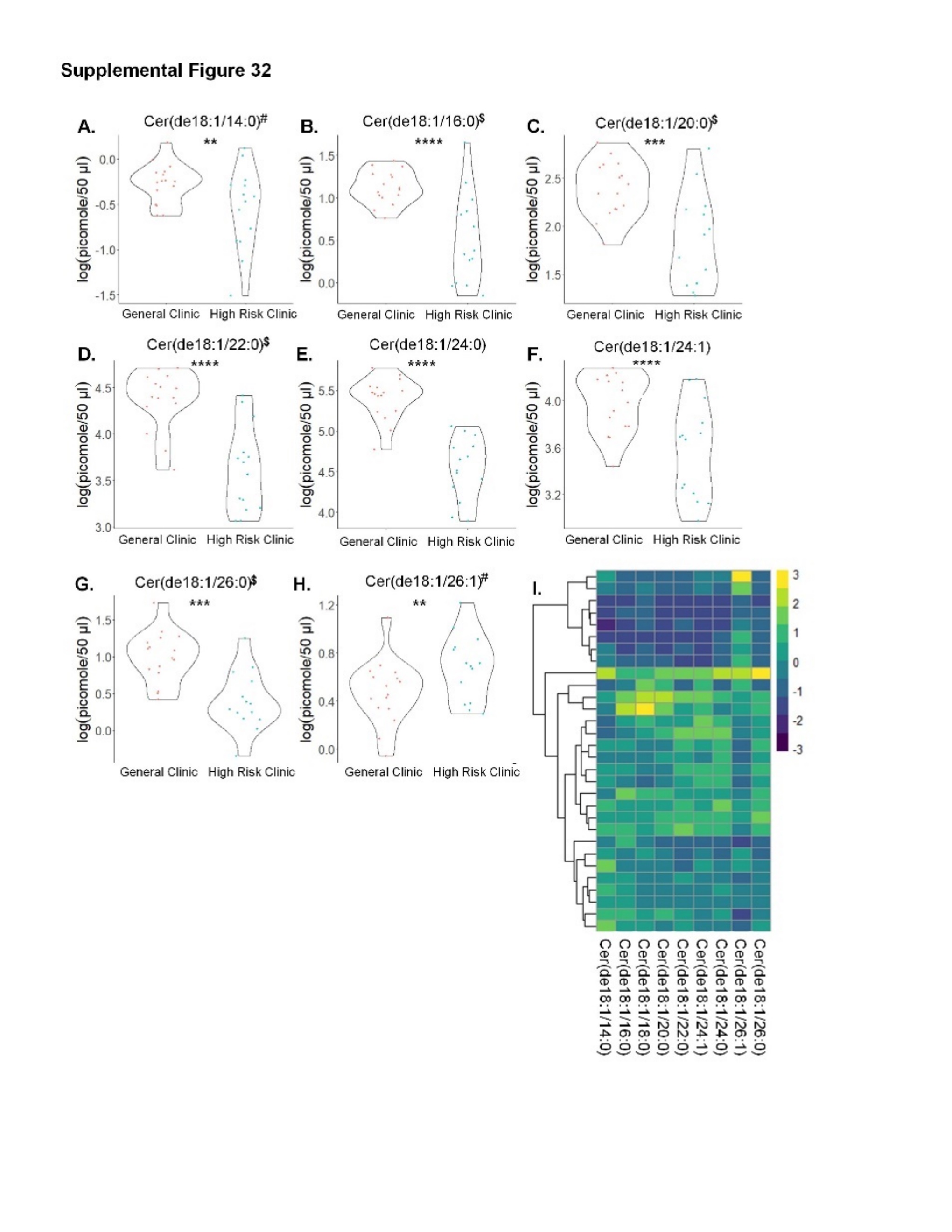


**Supplemental Figure 32. Ceramide species show significant differences in the plasma from uncomplicated term pregnant patients recruited from General OB/GYN clinic vs. uncomplicated term pregnant patients referred to High-Risk OB/GYN clinic. (A-H**) Ceramide species that occurred at significantly different levels when comparing plasma from normal uncomplicated term pregnant patients recruited from General OB/GYN clinic vs. normal uncomplicated term pregnant patients referred to High-Risk OB/GYN clinic using UPLC ESI-MS/MS as the detection method. Samples were analyzed by UPLC ESI-MS/MS within two weeks of acquisition. **(I)** Heatmap of all ceramide species that were detected via UPLC ESI-MS/MS in plasma (depicted as fold change). Samples were compared using unpaired students t-test with Welch’s correction. Data shown are means + SD depicted as violin plots, *P< 0.05, **P< 0.01, ***P< 0.001, ****P< 0.0001. The log transformed data failing the Shapiro-Wilk Test are designated with a $. Non-transformed data were also analyzed by the Wilcoxon Sum Rank Test. Bioactive lipid mediators not found to be significantlydifferent by the Wilcoxon Sum Rank Test are designated with a #.
